# The Effect of Extracorporeal Shockwave Therapy in Tendinopathy: A Systematic Review and Network Meta‐Analysis of Randomized Controlled Trials

**DOI:** 10.1111/os.70408

**Published:** 2026-09-13

**Authors:** Ning‐Yi Guo, Si‐Qi Wang, Wei Liu, Zi‐Mu Mao, Jian‐quan Wang, Bing‐bing Xu

**Affiliations:** ^1^ Department of Sports Medicine, Peking University Third Hospital Institute of Sports Medicine of Peking University Beijing China; ^2^ Beijing Key Laboratory of Research and Translation for Drugs and Medical Devices in Precision Diagnosis and Treatment of Sports Injuries Beijing China; ^3^ Engineering Research Center of Sports Trauma Treatment Technology and Devices Ministry of Education Beijing China; ^4^ Department of Sports Medicine and Exercise Rehabilitation Beijing Sport University Beijing China; ^5^ Department of Joint and Sports Medicine, Beijing Shijitan Hospital Capital Medical University Beijing China

**Keywords:** conservative treatment, extracorporeal shock wave therapy, tendinopathy

## Abstract

**Background:**

In recent years, numerous meta‐analyses have been published on the effectiveness of ESWT in treating various tendinopathies. However, due to limitations such as the small number of included studies, it remains unclear whether ESWT is definitively effective for all types of tendinopathies and what its comparative value is relative to other conservative treatments. The objective of this meta‐analysis is to compare ESWT with other conservative treatments to determine its effectiveness in alleviating pain and improving the severity of tendinopathies. Additionally, through network meta‐analysis, we aim to compare the efficacy of ESWT and other conservative treatments across different types of tendinopathies. This will help establish the value of ESWT in each type of tendinopathy, providing a theoretical basis for clinical decision‐making regarding ESWT treatment for various tendinopathies.

**Methods:**

In accordance with the Preferred Reporting Items for Systematic Reviews and Meta‐Analyses (PRISMA) guidelines, the PubMed, Embase, and Cochrane Library databases along with other databases were searched to identify relevant randomized controlled trials (RCTs). The quality of the selected studies was evaluated using risk of bias assessments, and the data were extracted. Network meta‐analysis was performed using random effects models to evaluate the effects of different treatment modalities on reducing pain and improving functional outcomes, reduction and functional improvement. The evidence of the included studies was evaluated using the Grading of Recommendations, Assessment, Development, and Evaluations (GRADE) framework.

**Results:**

This study included 65 publications from 2002 to 2024, with a total sample size of 3,921 cases. The included studies covered five types of tendinopathies: lateral epicondylitis, rotator cuff tendinopathy, Achilles tendinopathy, greater trochanteric pain syndrome, and patellar tendinopathy. Through pairwise subgroup meta‐analyses, we obtained the following representative results: for VAS in lateral epicondylitis, ESWT versus US, SMD −2.53 [95% CI −3.03 to −2.03], *I* ^2^ = 64.7%; ESWT versus PLACEBO, SMD −0.53 [95% CI −0.76 to −0.30], *I* ^2^ = 39.0%; for VAS in Achilles tendinopathy, ESWT versus PLACEBO, SMD −0.49 [95% CI −0.83 to −0.16], *I* ^2^ = 0; for VISA‐P in patellar tendinopathy, ESWT versus PLACEBO, SMD −0.15 [95% CI −0.42 to 0.12], *I* ^2^ = 0; for VAS in rotator cuff tendinopathy, ESWT versus PLACEBO, SMD −1.25 [95% CI −1.61 to −0.89], *I* ^2^ = 89.1%; for GTPS, ESWT versus EX, SMD −0.41 [95% CI −0.70 to −0.11], *I* ^2^ = 10.2%. In the network meta‐analysis, the following representative results were obtained: in LE, ESWT ranked second in SUCRA for improving VAS and PRTEE, with no significant difference from the first rank (ESWT vs. PDRN, MD 0.44 [95% CI −2.76 to 3.64]; ESWT vs. KT, MD 0.39 [95% CI −0.88 to 1.66]); in RCT, ESWT ranked second in SUCRA for improving SPADI, with no significant difference from the first rank (ESWT vs. EX, MD 0.15 [95% CI −0.24 to 0.55]); in AT, ESWT ranked second in SUCRA for improving VAS, with a significant difference from the first rank (ESWT vs. LT, MD 2.55 [95% CI 1.86–3.24]).

**Conclusions:**

Current limited evidence suggests that, compared to control groups, ESWT effectively improves pain and tendinopathy severity indicators in various tendinopathies except for patellar tendinopathy. However, its efficacy may vary across different tendinopathy types. Compared to other conservative treatments, ESWT holds a favorable position in treating lateral epicondylitis (LE), Achilles tendinopathy (AT), and rotator cuff tendinopathy (RCT). Notably, we found that ESWT does not show a significant therapeutic effect over placebo in the treatment of patellar tendinopathy.

**Abbreviations**
AT: Achilles tendinopathy
CSI: corticosteroid injection
ESWT: extracorporeal shock wave therapy
EX: exercise
GTPS: greater trochanteric pain syndrome
KT: kinesiotaping
LE: lateral epicondylitis
LT: laser therapy
ML: mobilization
NP: needle puncture
NT: neural therapy
PBMT: photobiomodulation therapy
PDRN: polydeoxyribonucleotide
PLT: prolotherapy
PRP: platelet‐rich plasma
PT: patellar tendinopathy
RCT: rotator cuff tendinopathy
RCTs: randomized controlled trials
US: ultrasound therapy
WS: wrist splint

## 1 Background

Tendinopathy (also known as tendinitis, tenosynovitis, and tendinosis) refers to pain and dysfunction in the tendons [1, 2]. It can be categorized into upper limb and lower limb tendinopathies, primarily including conditions such as lateral epicondylitis and rotator cuff tendinopathy for the upper limb, and greater trochanteric pain syndrome, patellar tendinopathy, and Achilles tendinopathy for the lower limb [3]. Currently, the prevalence of tendinopathy is increasing globally, with an incidence rate of 10.52 per 1000 person‐years for lower limb tendinopathies [4, 5]. Tendinopathy is even more common among athletes compared to the general population [6]. The associated pain and dysfunction from tendinopathy can significantly impact patients' quality of life and physical abilities, potentially resulting in long‐term or permanent functional impairments.

The pathological changes and mechanisms underlying tendinopathy remain incompletely understood [1]. A widely accepted model suggests that tendons are primarily composed of tenocytes, type I collagen, and proteoglycans [7]. When tendons are subjected to excessive load or overstretching, the typically orderly collagen fibers may fragment [8, 9], leading to tenocyte activation and proliferation. This cascade subsequently damages the collagen matrix and increases angiogenesis [10], ultimately reducing tendon performance and load‐bearing capacity [9], resulting in pain and functional impairment in patients. Due to the complexity of these pathological changes and the lack of a unified understanding of their mechanisms, treating tendinopathy remains challenging. Current therapeutic approaches targeting various potential mechanisms have yet to achieve complete cure rates [11], and the benefits of similar treatments can vary among different types of tendinopathies.

Extracorporeal shock wave therapy (ESWT) is a mechanical treatment that utilizes pulsed pressure waves to alter the chemical environment of the injury site [12]. This approach helps reduce local inflammation, reverse tissue damage, and promote tendon remodeling [13]. ESWT has become a widely accepted conservative alternative to surgery for treating tendinopathies in various areas such as the shoulder, elbow, hip, and ankle [14, 15]. It has been proven effective in relieving pain and enhancing motor function in patients with tendinopathy, with additional benefits including being noninvasive, low‐cost, and carrying relatively minimal treatment risk [15, 16]. In recent years, numerous meta‐analyses have examined ESWT's effectiveness in treating Achilles tendinopathy, lateral epicondylitis, patellar tendinopathy, and other types of tendinopathy [16]. However, due to factors like limited study numbers, inconsistent inclusion criteria, and a lack of comprehensive comparisons of ESWT's relative value across different types of tendinopathy, conclusions remain inconsistent. This leaves uncertainty regarding ESWT's efficacy across various tendinopathies and its comparative value as a conservative treatment option in different types of tendinopathy. Notably, no prior study has systematically compared the relative efficacy of ESWT against multiple conservative interventions across the full spectrum of common tendinopathies using network meta‐analysis (NMA), which precludes evidence‐based clinical decision‐making for ESWT use in daily practice.

The objective of this meta‐analysis is to systematically synthesize and evaluate evidence from randomized controlled trials (RCTs) to verify the therapeutic efficacy of ESWT in treating tendinopathy. Specifically, this study aims to achieve three goals: first, to compare ESWT with other conservative treatments in order to determine its effectiveness in alleviating pain and reducing the severity of tendinopathy; second, to evaluate and compare the efficacy of ESWT with other conservative treatments across different types of tendinopathies, thereby establishing the value of ESWT in each tendinopathy type; and finally, to thoroughly investigate potential sources of heterogeneity. Ultimately, our findings aim to provide theoretical support for the application of ESWT in tendinopathy treatment and offer a theoretical basis for clinical decision‐making on ESWT for various types of tendinopathy, potentially aiding further research on conservative treatments for tendinopathy. These findings will directly inform tiered conservative treatment recommendations for patients with different tendinopathy subtypes in clinical settings.

## 2 Method

### 2.1 Registration

This systematic review and network meta‐analysis were conducted following the Preferred Reporting Items for Systematic Reviews and Meta‐Analyses (PRISMA) guidelines [17]. The PRISMA NMA checklist is provided in Table S1. The study protocol was registered in the International Prospective Register of Systematic Reviews (PROSPERO) under ID: CRD42024583025.

### 2.2 Literature Search Strategy

The published and registered studies were retrieved from PubMed, Embase, Cochrane Library, Web of Science, and EBSCO databases. The registered but unpublished studies were obtained from the U.S. Clinical Trial registry. When conducting the search for published studies, MeSH and Entry terms were utilized. Detailed information regarding the search strategy implemented by each database is provided in Table S3. The search strategy is based on keywords associated with the PICO tool: (P) Population—individuals with tendinopathies; (I) Intervention—extracorporeal shock wave therapy (ESWT); (C) Comparator—sham treatments or other conservative therapy such as injection therapy, physical therapy or exercise; (O) Outcome measures—indicators of pain, functional or the severity of tendinopathies. Additionally, a manual search through references of selected articles and reviews was conducted to identify any relevant studies that may have been overlooked during electronic searches. Finally, all pertinent English‐language studies published before July 2024 were included.

### 2.3 Eligibility Criteria

Studies were eligible if they (1) were RCTs; (2) included patients with five types of tendinopathy (Lateral epicondylitis, Patellar tendinopathy, Achilles tendinopathy, Rotator Cuff Tendinosis, and Greater trochanter pain syndrome) without age restriction; the patients needed to be clinically diagnosed by a physiotherapist or medical doctor; the trials for network meta‐analysis needed to report follow‐up data for at least one of the outcome measures of interest (pain, patient‐reported physical function, or questionnaire to assess the severity of tendinopathies); (3) compared an experimental group receiving extracorporeal shock wave therapy for at least 3 weeks; (4) comparator: blank control, sham treatment or other conservative treatment such as injection therapy, physical therapy or exercise; (5) assessed the degree of pain, functional or the severity of tendinopathies before and after the intervention, with no restrictions on the measurement method.

Studies were excluded if they (1) were duplicate publications, were literature review papers, were letters to the editor, were case reports, had abstracts published in conference proceedings, reported acute effects of a single intervention session, and were animal model studies; or (2) were combination of extracorporeal shock wave therapy (ESWT) and other conservative treatments (e.g., ESWT combined with an exercise intervention, but there was no exercise intervention control group); (3) simply comparing different types of extracorporeal shock wave, for example, comparing radial extracorporeal shock wave (r‐ESWT) to focused extracorporeal shock wave (f‐ESWT); (4) have patients with previous tendon surgery.

Two researchers independently screened the articles based on inclusion and exclusion criteria. Multiple publications of the same trial were collated and the first or most complete report was used as a reference. If studies report men and women separately, the different genders are combined into one group.

### 2.4 Data Extraction

Two researchers independently extracted data from the included studies. Disagreements were resolved by consensus or by consulting a third author. The following data were extracted: first author, year of publication, subject characteristics (type of tendinopathy, number of experimental and control groups, sex, age, degree of pain, index of dysfunction, etc.), intervention information for ESWT (including modality [focused (f‐ESWT) or radial (r‐ESWT)], energy density, pulse number, duration, frequency, cycle, and whether supervised or unsupervised) as well as other conservative interventions (type of intervention, key implementation parameters), and the measurement method and unit of reported results. Given that ESWT efficacy is highly dependent on these technical parameters, the extracted ESWT‐related parameters were intended for subsequent subgroup analyses to explore potential sources of heterogeneity (e.g., comparing efficacy differences across different ESWT modalities or energy density ranges). However, due to inconsistent reporting of ESWT modality and parameter details across included studies, a formal subgroup analysis based on these parameters was not feasible, and this limitation is discussed in Section 4.6. If any data were missing, the authors of the included studies were contacted by email.

### 2.5 Risk of Bias and Certain of Evidence Assessments

Two investigators independently assessed the risk of bias (ROB) of the included studies using the Cochrane Risk of Bias Tool [18], which includes seven domains: (a) allocation generation, (b) concealment of allocation, (c) blinding of participants and personnel, (d) blinding of outcome assessment, (e) incomplete outcome data addressed, (f) freedom from selective reporting bias, and (g) other sources of bias. We subsequently rated the overall ROB of each study as follows: studies were classified as having an overall low ROB if none of the domains above were rated as having high ROB and three or fewer domains were rated as having an unclear risk; studies were classified as having an overall moderate ROB if one of the above domains was rated as having a high ROB or if four of the domains were rated as having an unclear risk of bias; otherwise, studies were classified as having an overall high ROB [19].

We assessed the certainty of evidence contributing to the network estimates of the outcome measures using the Grading of Recommendations Assessment, Development and Evaluation (GRADE) framework [20].

### 2.6 Data Synthesis and Statistical Analysis

The pre‐to‐post changes in the experimental and control groups were pooled to estimate the effects. Given the inherent heterogeneity of the interventions, pairwise meta‐analytic estimates are also reported alongside the network estimates to account for variability among studies, using STATA 15.1 software (StataCorp, College Station, TX, USA). Heterogeneity was analyzed through sensitivity analysis. A random effects model was used to obtain pooled estimates. Standardized mean differences (SMDs) with 95% confidence intervals (CIs) were calculated for patient‐reported pain, measured by the Visual Analogue Scale (VAS) and Numeric Rating Scale (NRS), as well as for tendinopathy severity, measured by the Victoria Institute of Sport—Patellar score (VISA‐P), Victoria Institute of Sport—Achilles score (VISA‐A), Shoulder Pain and Disability Index (SPADI) [21], and Patient‐Rated Tennis Elbow Evaluation (PRTEE) [22]. For multi‐arm trials included in both pairwise and network meta‐analyses, all eligible intervention arms were retained and analyzed as separate comparisons, with appropriate adjustment of the covariance structure to avoid double‐counting of shared control groups. The *I* ^2^ statistic and Cochran's *Q* test were used to quantify heterogeneity, with *I* ^2^ values above 50% or a *p* value of 0.10 or less for the *Q* test indicating substantial heterogeneity [23]. Publication bias was evaluated by inspecting funnel plots and performing Egger's test.

STATA 15.1 software (StataCorp, College Station, TX, USA) was used to conduct random effects multivariate network meta‐analysis (NMA) within a frequentist framework [24], following current PRISMA NMA guidelines [17]. Given that different tools and units were used to measure pain and dysfunction in the included studies, the mean difference (MD) was used to represent the effect size across outcome measures.

The relationships among ESWT and other conservative interventions are illustrated in a network diagram, where the lines connecting nodes represent direct head‐to‐head comparisons between interventions, and the size of each node and the thickness of each connecting line are proportional to the number of studies. A network contribution graph was created to calculate the contribution of each direct comparison.

The transitivity assumption was assessed by reviewing the inclusion criteria of individual studies, determining whether all participants in the network could have been randomly assigned to any intervention, and by applying consistency models [25]. Transitivity, a key assumption of NMA, indicates that indirect comparisons are valid estimates of unobserved direct comparisons [24] and that effect modifiers are homogeneously distributed across studies [26]. Inconsistency factors (IFs) with 95% CIs were calculated to assess the consistency of each closed loop, with consistency indicated if the 95% CI of the IF included 0 (i.e., no significant inconsistency across direct and indirect comparisons) [27]. The inconsistency model was used to test for global inconsistency, and the consistency model was applied for final effect size estimation when global inconsistency was nonsignificant (*p* > 0.05) [28]. Node‐splitting analysis was further conducted to check for local inconsistency between direct and indirect evidence for each intervention comparison; a *p* value > 0.05 was considered indicative of no significant local inconsistency, supporting the validity of combining direct and indirect evidence under the consistency model. Results of the transitivity assessment, IFs, and node‐splitting analysis will be explicitly summarized in the Results section (with detailed data provided in Supporting Information) to verify the robustness of NMA findings.

The surface under the cumulative ranking curve (SUCRA) was used to rank and compare the effectiveness of different conservative interventions [29]. SUCRA values range from 0 to 100, where 100 signifies the highest probability of being the most effective treatment and 0 signifies the lowest [30]. Notably, SUCRA represents a probability‐based relative ranking and does not by itself indicate statistically significant superiority between interventions; formal statistical comparisons with 95% confidence intervals are required to determine significant differences in efficacy. Thus, higher SUCRA values indicate higher probability of favorable outcomes for ESWT or other conservative interventions in treating tendinopathies. To examine NMA publication bias due to small‐scale studies, a network funnel plot was generated, and visual analysis of symmetry was performed.

## 3 Results

### 3.1 Result of Search

In the initial search, a total of 1374 articles were retrieved. After removing 576 duplicates and 340 studies that did not meet the inclusion criteria (such as reviews, systematic evaluations, and animal studies), 458 articles remained for title and abstract screening. During this process, 235 articles were excluded due to issues with study design or intervention methods. Subsequently, the remaining 223 articles were reviewed in full, and an additional 158 were excluded due to lack of access to full texts, data that did not meet study requirements, or incorrect study design. Ultimately, 65 randomized controlled trials (RCTs) on the effects of extracorporeal shock wave therapy (ESWT) for tendinopathy were included in this meta‐analysis [31, 32, 33, 34, 35, 36, 37, 38, 39, 40, 41, 42, 43, 44, 45, 46, 47, 48, 49, 50, 51, 52, 53, 54, 55, 56, 57, 58, 59, 60, 61, 62, 63, 64, 65, 66, 67, 68, 69, 70, 71, 72, 73, 74, 75, 76, 77, 78, 79, 80, 81, 82, 83, 84, 85, 86, 87, 88, 89, 90, 91, 92, 93, 94, 95]. The full list of included studies is provided in Table S4. The selection process of included studies is illustrated in the PRISMA flowchart (Figure 1).

**FIGURE 1:**
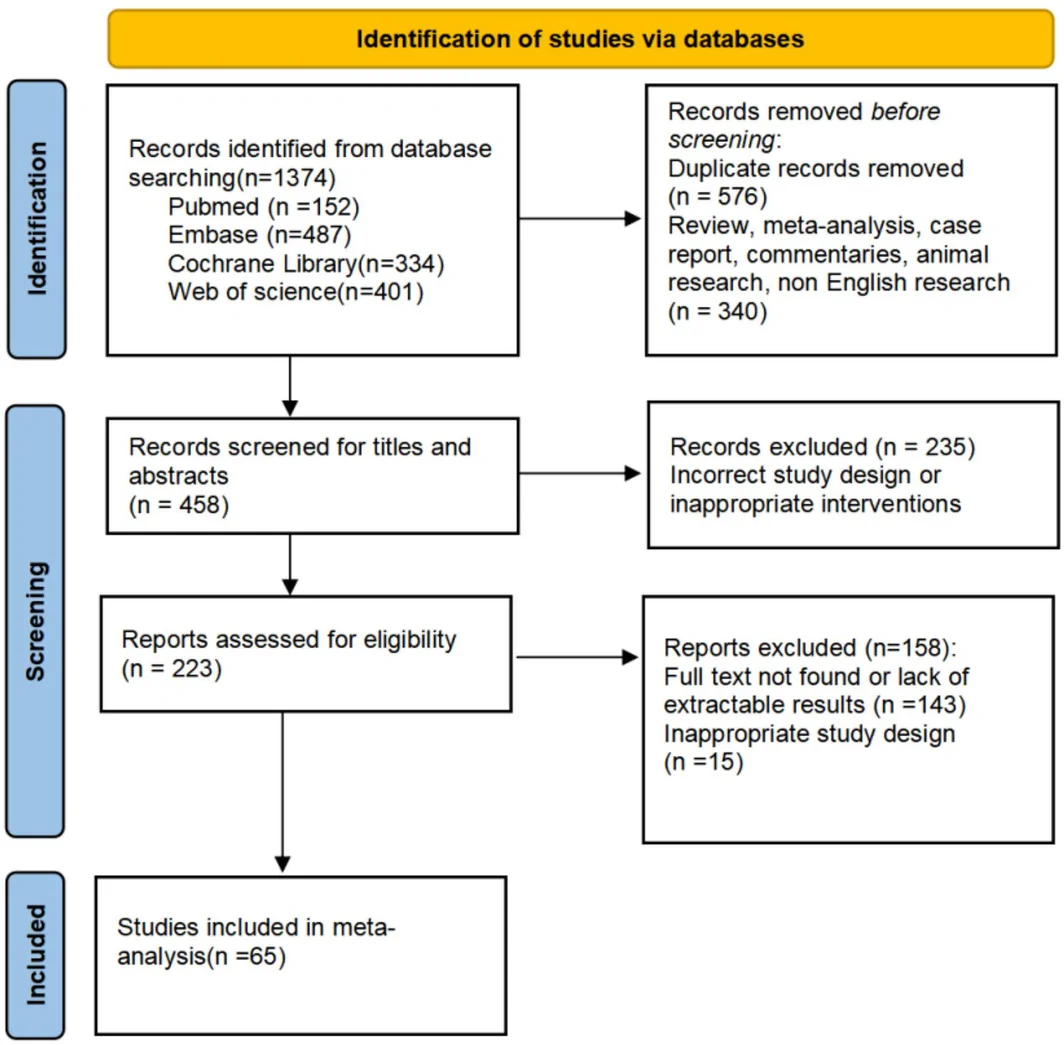
PRISMA flow diagram of the study selection process.

### 3.2 Characteristics of the Included Studies

This meta‐analysis included 65 studies published between 2002 and 2024, with a total sample size of 3921 participants (details on the gender ratio and mean age in each study can be found in the Table S2). The studies cover five types of tendinopathies: lateral epicondylitis (*n* = 27), rotator cuff tendinopathy (*n* = 12), Achilles tendinopathy (*n* = 11), greater trochanteric pain syndrome (GTPS, *n* = 7), and patellar tendinopathy (*n* = 8). Six outcome measures were included in this study: VAS, NRS, VISA‐P, VISA‐A, PRTEE, and SPADI.

In the included studies, the intervention for the experimental group was extracorporeal shock wave therapy (ESWT), while the control groups received one of 14 different conservative treatments: Laser Therapy (LT, *n* = 4), corticosteroid injection (CSI, *n* = 6), exercise (EX, *n* = 6), ultrasound therapy (US, *n* = 9), placebo (*n* = 32), needle puncture (NP, *n* = 1), kinesiotaping (KT, *n* = 3), platelet‐rich plasma (PRP, *n* = 3), polydeoxyribonucleotide (PDRN, *n* = 1), neural therapy (NT, *n* = 1), photobiomodulation therapy (PBMT, *n* = 1), prolotherapy (PLT, *n* = 1), mobilization (ML, *n* = 1), and wrist splint (WS, *n* = 1). The agreement between the two assessors was 84.4% for study selection and 86.1% for data extraction.

### 3.3 Quality Assessment of the Included Studies

The overall risk of bias (ROB) for the 65 included studies is illustrated in Figure 2. The risk of bias assessment for each individual study is presented in Figure S1. Most studies demonstrated a low risk of bias in the areas of random sequence generation, allocation concealment, and handling of incomplete outcome data, with 63, 49, and 56 studies, respectively, meeting these criteria (accounting for 96.9%, 75.4%, and 86.2%). Additionally, all studies had a low risk of bias for selective reporting. However, because many of the interventions required participants and researchers to have prior knowledge of the treatment protocols, 15 studies had an unclear risk of bias in participant blinding, and one study had a high risk. In terms of researcher blinding, 14 studies had an unclear risk, and 13 had a high risk of bias.

**FIGURE 2:**
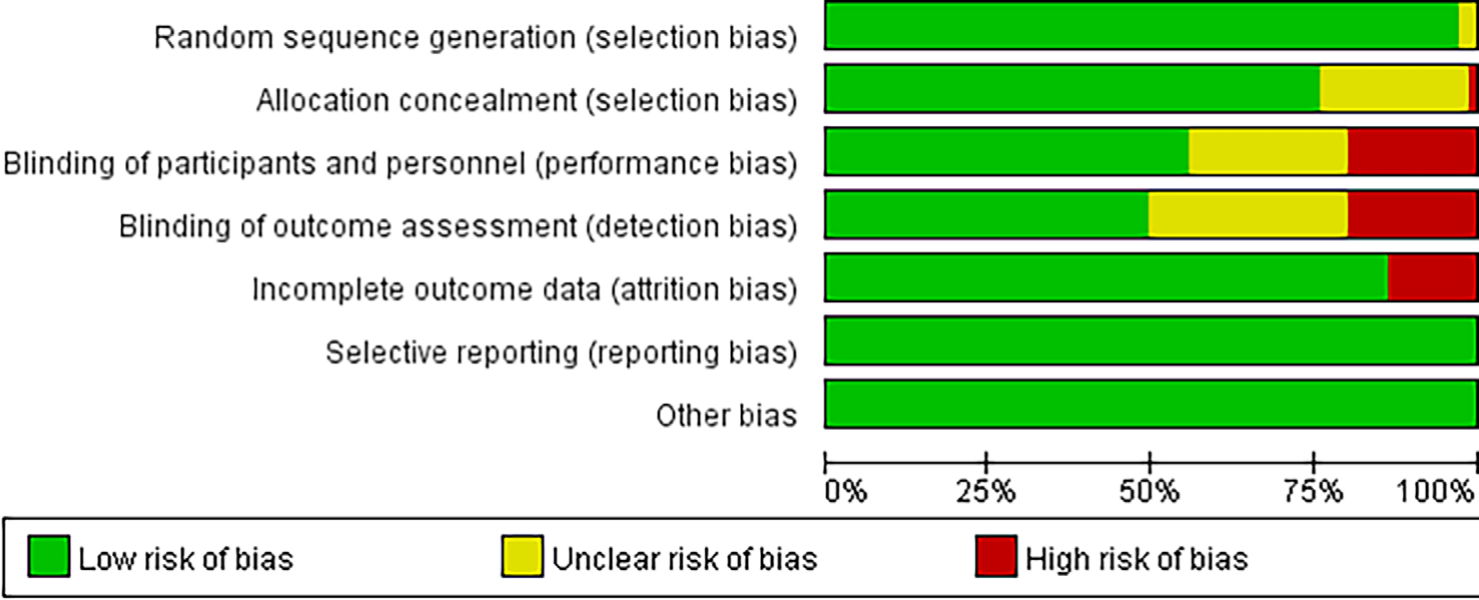
Overall risk of bias of the included studies.

In summary, 36 studies were judged to have a low ROB, 23 had a moderate ROB, and 6 had a high ROB.

### 3.4 Pairwise Meta‐Analysis

First, we assessed heterogeneity across all 65 included studies (all of which involved pain‐related measures, though not standardized to a single scale), and found significant heterogeneity (Figure S2). An initial subgroup analysis was conducted on 45 studies using the 0–10 VAS pain scale as the outcome measure, but heterogeneity remained high (Figure S3). Next, we grouped studies by control intervention and tendinopathy type to assess heterogeneity, but this also failed to significantly reduce heterogeneity (forest plots and publication bias tests are detailed in the Figure S4). Finally, we conducted a subgroup analysis based on specific outcome measures, tendinopathy types, and control interventions, with the results presented by tendinopathy type for each subgroup.

#### 3.4.1 Lateral Epicondylitis

*VAS*: We performed a subgroup analysis on 13 studies involving lateral epicondylitis with available VAS score data (Figure 3A), dividing them into three subgroups based on control interventions: ultrasound therapy (US), placebo control (PLACEBO), and kinesiotaping (KT). As shown in the forest plot, ESWT demonstrated a significant reduction in VAS scores when compared to US and placebo, with a marked decrease in heterogeneity (ESWT vs. US, SMD −2.53 [95% CI −3.03 to −2.03], *I* ^2^ = 64.7%, *p* for heterogeneity = 0.059; ESWT vs. PLACEBO, SMD −0.53 [95% CI −0.76 to −0.30], *I* ^2^ = 39.0%, *p* for heterogeneity = 0.132). However, when compared to KT, ESWT did not significantly impact VAS scores, and high heterogeneity was observed (ESWT vs. KT, SMD 0.64 [95% CI 0.14–1.13], *I* ^2^ = 96.5%, *p* for heterogeneity = 0.00).

**FIGURE 3:**
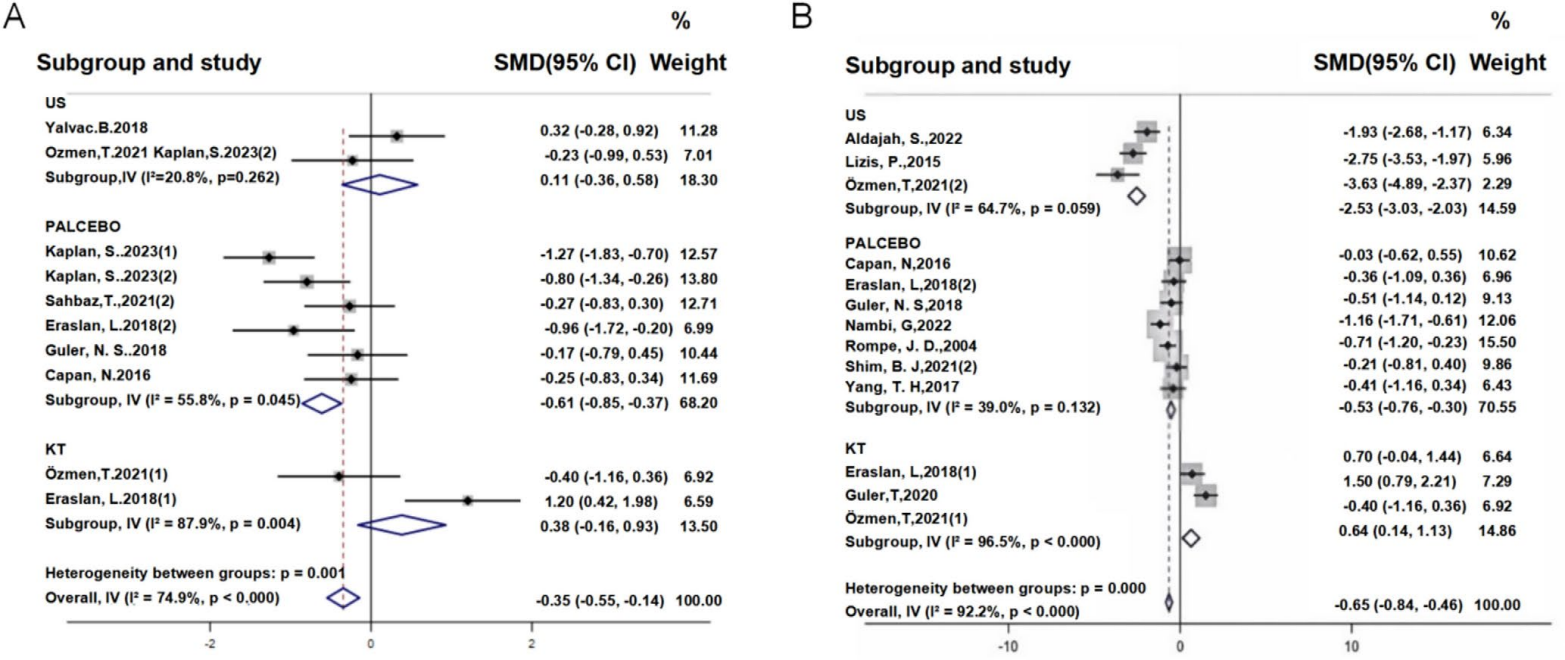
Heterogeneity analysis for VAS (A) and PRTEE (B) of lateral epicondylitis (VAS, visual analogue scale; PRTEE, patient‐rated tennis elbow evaluation; US, ultrasound therapy; KT, kinesiotaping).

*PRTEE*: We conducted a subgroup analysis on 10 studies involving lateral epicondylitis with PRTEE score data, using the same grouping method as described above (Figure 3B). As shown in the forest plot, ESWT significantly reduced PRTEE scores compared to placebo, with a notable reduction in heterogeneity (ESWT vs. PLACEBO, SMD −0.61 [95% CI −0.85 to −0.37], *I* ^2^ = 55.8%, *p* for heterogeneity = 0.045). However, ESWT did not significantly impact PRTEE scores when compared to US or KT (ESWT vs. US, SMD 0.11 [95% CI −0.36 to 0.58], *I* ^2^ = 20.6%, *p* for heterogeneity = 0.262; ESWT vs. KT, SMD 0.38 [95% CI −0.16 to 0.93], *I* ^2^ = 87.9%, *p* for heterogeneity = 0.004).

In summary, the meta‐analysis results indicate that for patients with lateral epicondylitis, ESWT significantly improves VAS and PRTEE scores compared to placebo. When compared to ultrasound therapy (US), ESWT shows a significant improvement in VAS scores but has no significant effect on PRTEE scores.

#### 3.4.2 Achilles Tendinopathy

*VAS*: We conducted a subgroup analysis on five studies involving Achilles tendinopathy with available VAS score data (Figure 4A), dividing them into two subgroups based on control interventions: exercise therapy (EX) and placebo or blank control (PLACEBO). As shown in the forest plot, ESWT significantly reduced VAS scores compared to placebo, with no heterogeneity observed (ESWT vs. PLACEBO, SMD −0.49 [95% CI −0.83 to −0.16], *I* ^2^ = 0, *p* for heterogeneity = 0.478). In contrast, ESWT did not significantly impact VAS scores when compared to exercise therapy, and heterogeneity remained absent (ESWT vs. EX, SMD −0.06 [95% CI −0.35 to 0.23], *I* ^2^ = 0, *p* for heterogeneity = 0.781).

**FIGURE 4:**
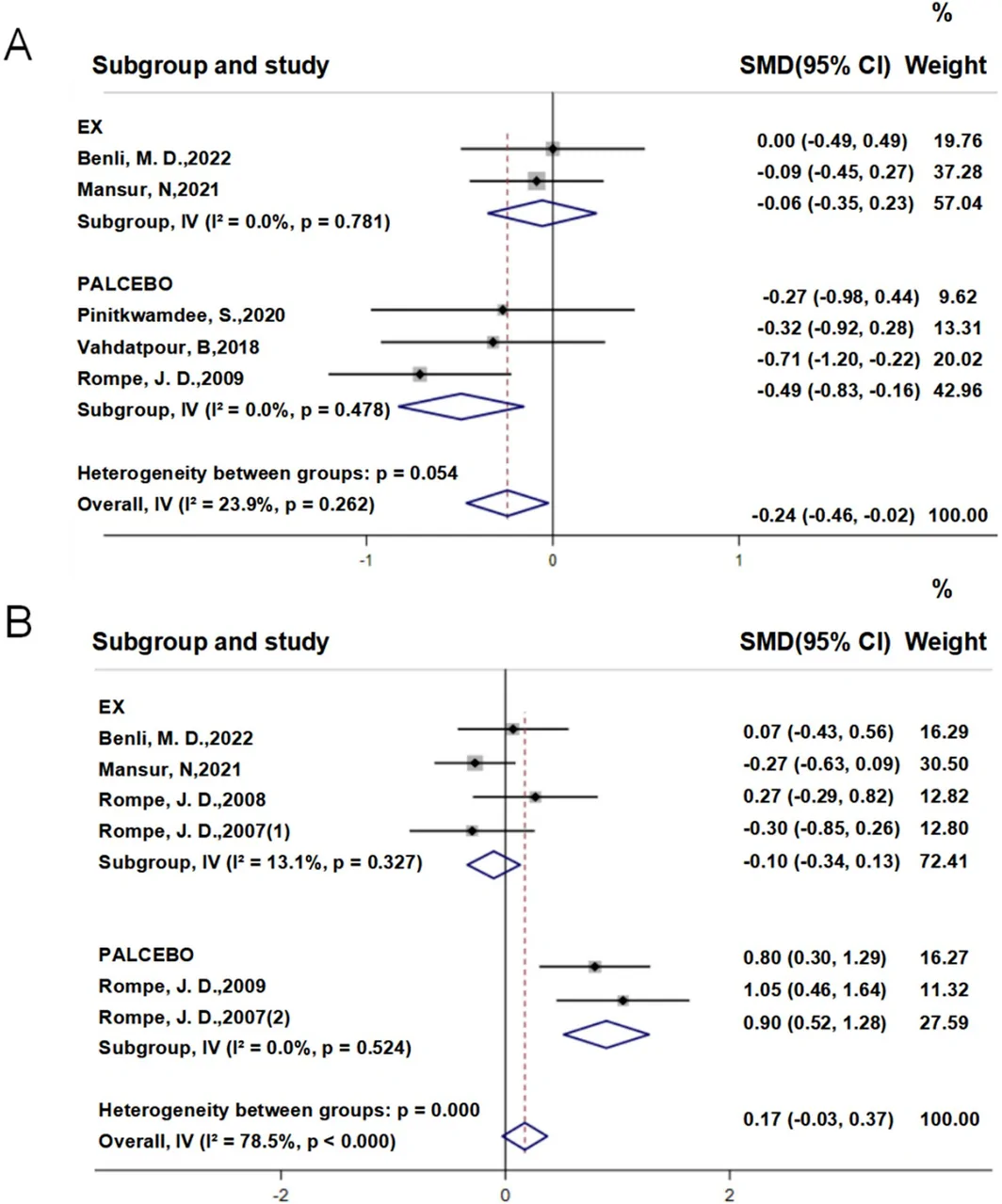
Heterogeneity analysis for VAS (A) and VISA‐A (B) of Achilles tendinopathy (VAS, visual analogue scale; VISA‐P, The Victorian Institute of Sport Assessment Scale for Patellar Tendinopathy; EX, exercise).

*VISA‐A*: We conducted a subgroup analysis on six studies involving Achilles tendinopathy with VISA‐A score data, using the same grouping method as described above (Figure 4B). As shown in the forest plot, ESWT significantly improved VISA‐A scores compared to placebo, with no notable heterogeneity (ESWT vs. PLACEBO, SMD 0.90 [95% CI 0.52–1.28], *I* ^2^ = 0, *p* for heterogeneity = 0.524). However, ESWT did not significantly affect VISA‐A scores compared to exercise therapy, and heterogeneity was minimal (ESWT vs. EX, SMD −0.10 [95% CI −0.34 to 0.13], *I* ^2^ = 13.1%, *p* for heterogeneity = 0.327).

In summary, the meta‐analysis results indicate that for patients with Achilles tendinopathy, ESWT demonstrates a significant improvement in both VAS and VISA‐A scores compared to placebo. However, there is no significant difference in therapeutic effect between ESWT and exercise therapy.

#### 3.4.3 Patellar Tendinopathy

*VAS*: A subgroup analysis was conducted on six studies concerning patellar tendinopathy that included VAS score data (Figure 5A). The studies were divided into two subgroups based on control measures: ultrasound therapy (US) and placebo or blank control (PLACEBO). The results indicate that ESWT significantly reduced VAS scores compared to both US and placebo in patients with patellar tendinopathy. Furthermore, the US subgroup showed no heterogeneity (ESWT vs. US, SMD −3.52 [95% CI −4.14 to −2.90], *I* ^2^ = 0, *p* for heterogeneity = 0.993; ESWT vs. PLACEBO, SMD −0.40 [95% CI −0.69 to −0.12], *I* ^2^ = 69.2%, *p* for heterogeneity = 0.021).

**FIGURE 5:**
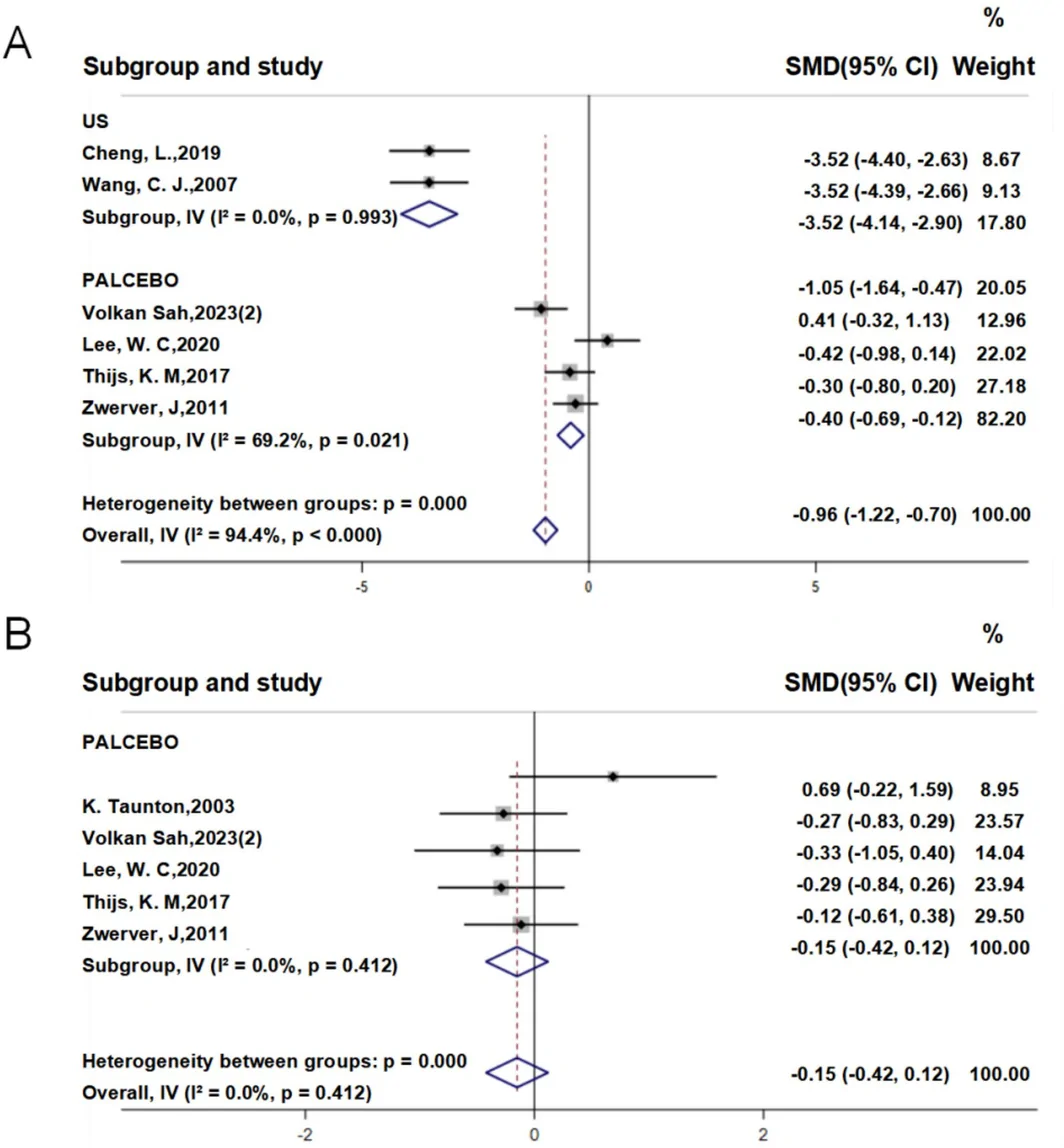
Heterogeneity analysis for VAS (A) and VISA‐P (B) of patellar tendinopathy (VAS, visual analogue scale; VISA‐P, The Victorian Institute of Sport Assessment Scale for Patellar Tendinopathy; US, ultrasound therapy).

*VISA‐P*: An analysis was performed on six studies concerning patellar tendinopathy with VISA‐P score data (Figure 5B), involving only one control measure: placebo or blank control (PLACEBO). The results indicated that ESWT did not have a significant effect on the VISA‐P scores of patients with patellar tendinopathy compared to placebo, and there was no heterogeneity in the results (ESWT vs. PLACEBO, SMD −0.15 [95% CI −0.42 to 0.12], *I* ^2^ = 0%, *p* for heterogeneity = 0.412).

In summary, the meta‐analysis on patellar tendinopathy indicates that ESWT has a significant effect on improving VAS scores when compared to both ultrasound therapy and placebo treatment. However, regarding the VISA‐P scores, there is no significant difference between ESWT and placebo.

#### 3.4.4 Rotator Cuff Tendinosis

*VAS*: A subgroup analysis was conducted on four studies on rotator cuff tendinopathy with VAS score data (Figure 6A). Based on the control measures, the studies were divided into two subgroups: exercise therapy (EX) and a blank control or placebo (PLACEBO). The results indicated that ESWT showed a significant effect in reducing VAS compared to PLACEBO, while there was no significant difference when compared to EX. However, both groups exhibited considerable heterogeneity (ESWT vs. EX, SMD −0.08 [95% CI −0.44 to 0.28], *I* ^2^ = 94.2%, *p* for heterogeneity = 0.00; ESWT vs. PLACEBO, SMD −1.25 [95% CI −1.61 to −0.89], *I* ^2^ = 89.1%, *p* for heterogeneity = 0.00) (Figure 6A,B).

**FIGURE 6:**
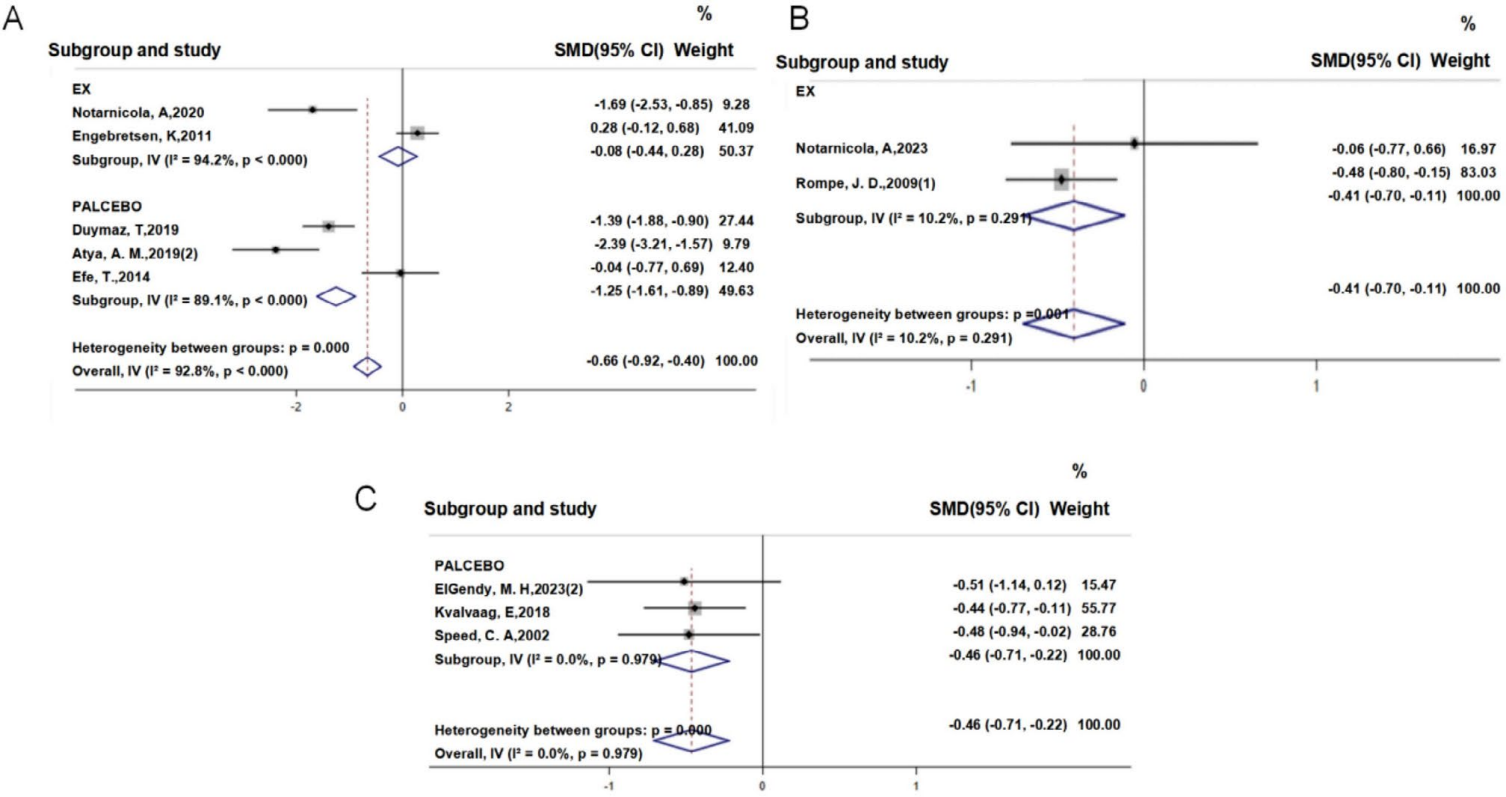
Heterogeneity analysis for VAS (A) and SPADI (B) of rotator cuff tendinopathy; heterogeneity analysis for NRS of GTPS (C) (VAS, visual analogue scale; SPADI, patient‐rated tennis elbow evaluation; NRS, numerical rating scale; EX, exercise).

*SPADI*: A subgroup analysis was conducted on three studies on rotator cuff tendinopathy with SPADI score data (Figure 6B), involving only one type of control measure: blank control or placebo (PLACEBO). The results indicated that ESWT had a significant effect in reducing SPADI scores compared to PLACEBO, with no heterogeneity in the results (ESWT vs. PLACEBO, SMD −0.46 [95% CI −0.71 to −0.22], *I* ^2^ = 0, *p* for heterogeneity = 0.979) (Figure 6C).

In summary, the meta‐analysis of rotator cuff tendinopathy showed that ESWT had a significant effect in improving SPADI scores compared to placebo treatment, with no heterogeneity in the results.

#### 3.4.5 Greater Trochanter Pain Syndrome (GTPS)

*NRS*: A subgroup analysis was conducted on two studies on greater trochanteric pain syndrome (GTPS) with NRS score data (Figure 6C), involving only one type of control measure: exercise therapy (EX). The results indicated that ESWT had a significant effect in reducing NRS scores in GTPS patients compared to EX, with no significant heterogeneity in the results (ESWT vs. EX, SMD −0.41 [95% CI −0.70 to −0.11], *I* ^2^ = 10.2%, *p* for heterogeneity = 0.291).

### 3.5 Network Meta‐Analysis (NMA)

Global inconsistency tests showed no significant inconsistency for any disease‐specific network (all *p* > 0.05). Node‐splitting analyses indicated no significant local inconsistency for the majority of comparisons (*p* > 0.05); detailed results are provided in the Figure S6. We first conducted a network meta‐analysis on all studies with 0–10 scale VAS score data. Subsequently, based on differentiated outcome measurement scales, network meta‐analyses were performed separately for five types of tendinopathy. The results were presented in multiple forms, including network evidence plots, SUCRA (Surface Under the Cumulative Ranking) scores, and ranking tables based on the area under the SUCRA curve.

#### 3.5.1 All Types of Tendinopathy

*VAS*: As an exploratory analysis, we conducted a network meta‐analysis on 42 studies (88 arms) containing VAS score data across all tendinopathy types (Figure 7), including Extracorporeal Shock Wave Therapy (ESWT, *n* = 44) and 14 other conservative treatment methods: Laser Therapy (LT, *n* = 2), corticosteroid injection (CSI, *n* = 1), Exercise (EX, *n* = 6), Ultrasound Therapy (US, *n* = 4), PLACEBO (*n* = 19), Needle Puncture (NP, *n* = 1), kinesiotaping (KT, *n* = 3), Platelet‐rich plasma (PRP, *n* = 2), Polydeoxyribonucleotide (PDRN, *n* = 1), Neural Therapy (NT, *n* = 1), Photobiomodulation Therapy (PBMT, *n* = 1), Prolotherapy (PLT, *n* = 1), mobilization (ML, *n* = 1), and Wrist Splint (WS, *n* = 1). The results of the network meta‐analysis, as shown in the Figure 7, indicated that among the 15 conservative treatment methods, Laser Therapy ranked first in terms of the area under the SUCRA curve (SUCRA 82.7%), while ESWT ranked fourth (65.5%). Additionally, we generated a comparison‐adjusted funnel plot, contribution plot (Figure S9), network forest plot, and funnel plot for risk of bias assessment for this network meta‐analysis, as detailed in the Figure S7.

**FIGURE 7:**
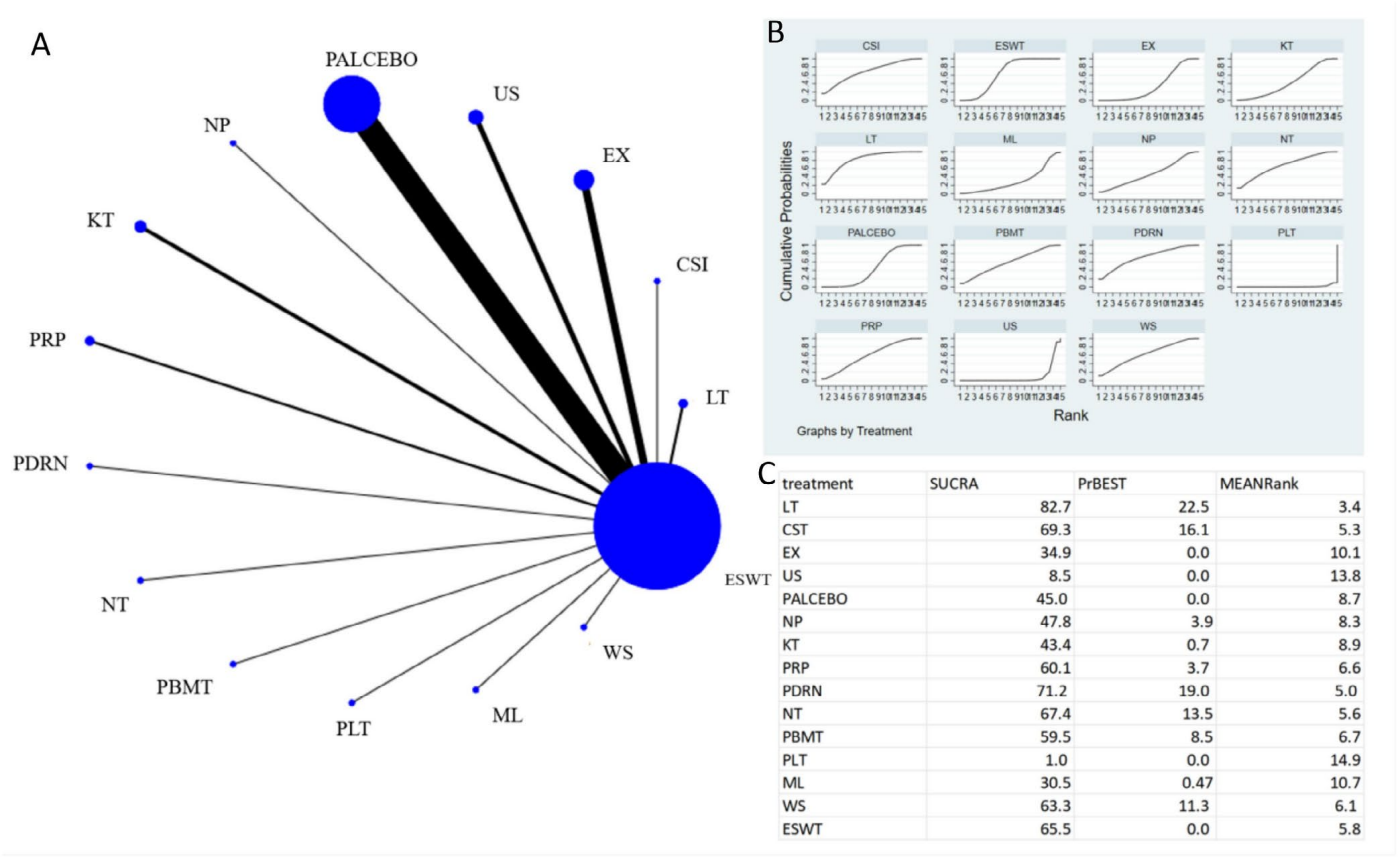
Network diagrams (A), cumulative probability graphs (B), and efficacy ranking table (treatment‐relative ranking) (C) for the VAS in all types of tendinopathy. In the network diagrams (A), node size is proportional to the total number of included participants, and edge thickness is proportional to the number of direct head‐to‐head comparisons between interventions.

#### 3.5.2 Lateral Epicondylitis

*VAS*: A network meta‐analysis was conducted on 21 studies (42 arms) containing VAS score data (Figure 8A–C), including Extracorporeal Shock Wave Therapy (ESWT, *n* = 21) and 11 other conservative treatment methods: Laser Therapy (LT, *n* = 1), Exercise (EX, *n* = 1), Ultrasound Therapy (US, *n* = 3), PLACEBO (*n* = 7), kinesiotaping (KT, *n* = 3), Platelet‐rich plasma (PRP, *n* = 1), Polydeoxyribonucleotide (PDRN, *n* = 1), Neural Therapy (NT, *n* = 1), Photobiomodulation Therapy (PBMT, *n* = 1), Prolotherapy (PLT, *n* = 1), and Wrist Splint (WS, *n* = 1). The results of the network meta‐analysis, as shown in the figure, indicated that among the 12 conservative treatment methods, Polydeoxyribonucleotide (PDRN) ranked first in terms of the area under the SUCRA curve (SUCRA 71.0%), while ESWT ranked second. Although ranked second, this did not reach statistical significance compared to the first‐ranked intervention. There was no significant difference between the two treatments (SUCRA 67.8%; ESWT vs. PDRN, MD 0.44 [95% CI −2.76 to 3.64]).

**FIGURE 8:**
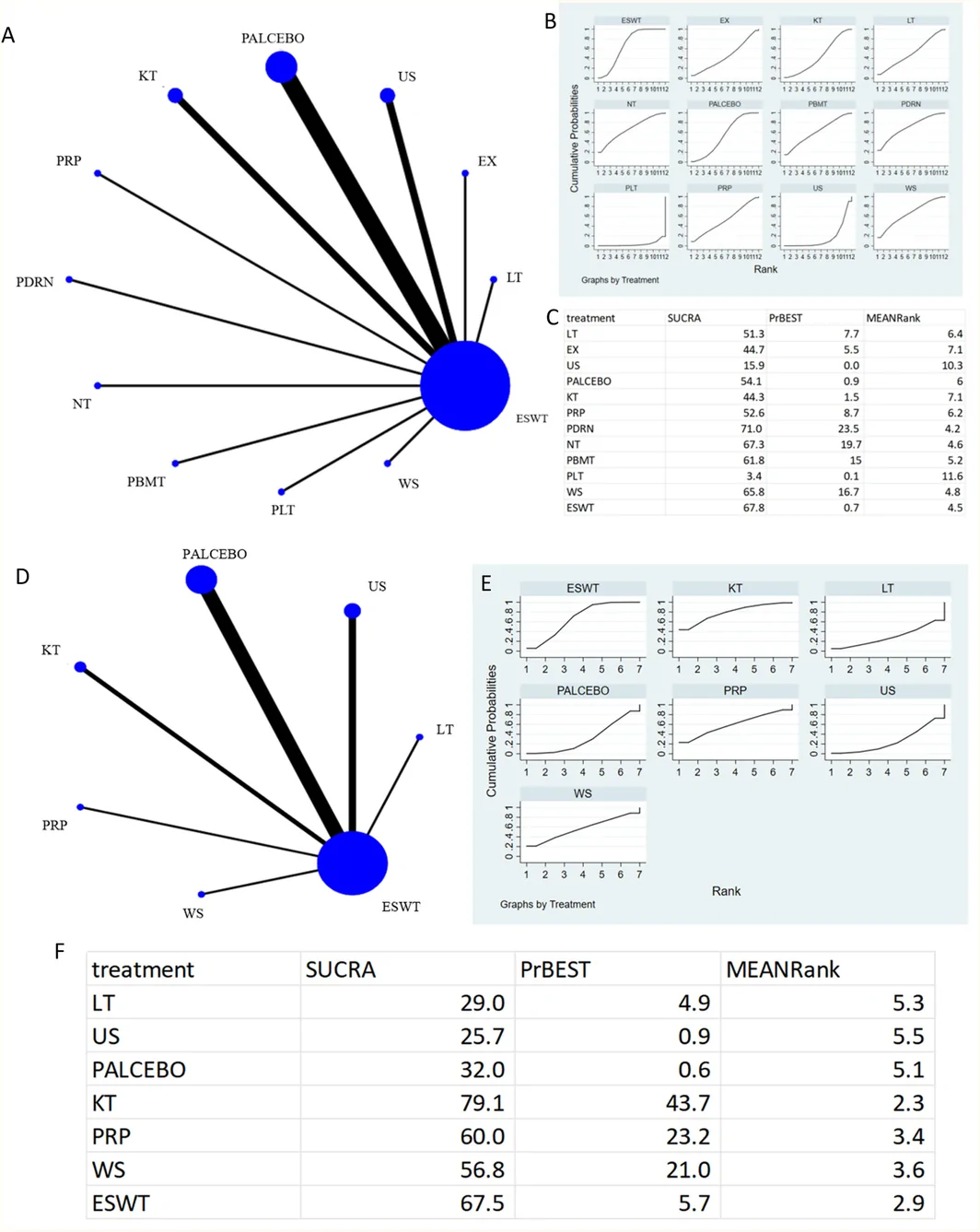
Network diagrams (A), cumulative probability graphs (B), and efficacy ranking table (treatment‐relative ranking) (C) for the VAS in lateral epicondylitis; network diagrams (D), cumulative probability graphs (E), and efficacy ranking table (treatment‐relative ranking) (F) for the PRTEE in lateral epicondylitis. In the network diagrams (A), node size is proportional to the total number of included participants, and edge thickness is proportional to the number of direct head‐to‐head comparisons between interventions.

*PRTEE*: A network meta‐analysis was conducted on 14 studies (28 arms) containing PRTEE score data (Figure 8D–F), including Extracorporeal Shock Wave Therapy (ESWT, *n* = 14) and six other conservative treatment methods: Laser Therapy (LT, *n* = 1), Ultrasound Therapy (US, *n* = 3), PLACEBO (*n* = 6), kinesiotaping (KT, *n* = 2), Platelet‐rich plasma (PRP, *n* = 1), and Wrist Splint (WS, *n* = 1). As shown in the results figure, among the seven conservative treatments, kinesiotaping (KT) ranked first in terms of the area under the SUCRA curve (SUCRA 79.1%), while ESWT ranked second, with no significant difference between the two treatments (SUCRA 67.5%; ESWT vs. KT, MD 0.39 [95% CI −0.88 to 1.66]). Additionally, we generated a comparison‐adjusted funnel plot, contribution plot, network forest plot, and funnel plot for risk of bias assessment for this network meta‐analysis, as detailed in the Supporting Information.

In summary, the network meta‐analysis for patients with lateral epicondylitis demonstrated that Extracorporeal Shock Wave Therapy showed prominent effects in improving both VAS and PRTEE scores, ranking second in the SUCRA curve area among the included conservative treatment methods.

#### 3.5.3 Patellar Tendinopathy

*VAS*: A network meta‐analysis was conducted on eight studies (16 arms) containing VAS score data (Figure 9A–C), including Extracorporeal Shock Wave Therapy (ESWT, *n* = 8) and four other conservative treatments: Exercise (EX, *n* = 1), Ultrasound Therapy (US, *n* = 1), Placebo (*n* = 5), and Platelet‐Rich Plasma (PRP, *n* = 1). As shown in the network meta‐analysis results figure, among the five conservative treatments, Platelet‐Rich Plasma (PRP) ranked first in the SUCRA curve area (SUCRA 88.5%), with ESWT ranking second. Although ranked second, this did not reach statistical significance compared to the first‐ranked intervention. There was no significant difference between the two (SUCRA 75.3%; ESWT vs. PRP, MD 0.37 [95% CI −0.87 to 1.61]).

**FIGURE 9:**
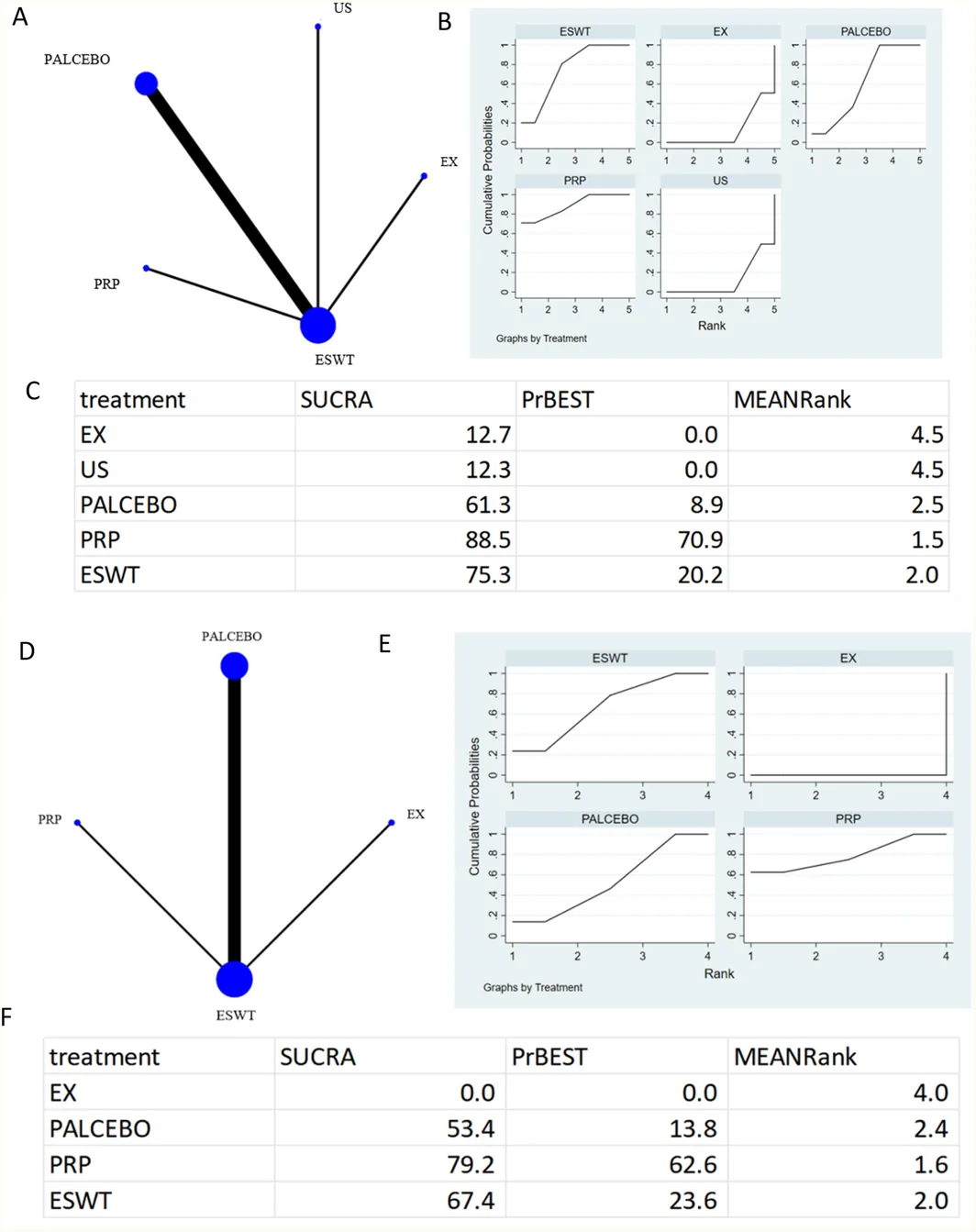
Network diagrams (A), cumulative probability graphs (B), and efficacy ranking table (treatment‐relative ranking) (C) for the VAS in lateral epicondylitis; network diagrams (D), cumulative probability graphs (E), and efficacy ranking table (treatment‐relative ranking) (F) for VISA‐P in patellar tendinopathy. In the network diagrams (A), node size is proportional to the total number of included participants, and edge thickness is proportional to the number of direct head‐to‐head comparisons between interventions.

*VISA‐P*: A network meta‐analysis was conducted on eight studies (16 arms) containing VISA‐P score data (Figure 9D–F), including ESWT (*n* = 8) and three other conservative treatments: Exercise (EX, *n* = 1), Placebo (*n* = 6), and Platelet‐Rich Plasma (PRP, *n* = 1). As shown in the network meta‐analysis results figure, among the four conservative treatments, Platelet‐Rich Plasma (PRP) ranked first in the SUCRA curve area (SUCRA 79.2%), with ESWT ranking second. There was no significant difference between the two (SUCRA 67.4%; ESWT vs. PRP, MD −0.27 [95% CI −1.46 to 0.92]). Additionally, we generated a comparison‐adjusted funnel plot, contribution plot, network forest plot, and funnel plot for bias assessment for this network meta‐analysis, as detailed in the Supporting Information.

In summary, in the network meta‐analysis for patients with patellar tendinopathy, we found that Extracorporeal Shock Wave Therapy demonstrated a significant effect in improving both VAS and VISA‐P scores, ranking second in the SUCRA curve area among the included conservative treatments, only behind PRP therapy.

#### 3.5.4 Achilles Tendinopathy

*VAS*: A network meta‐analysis was conducted on six studies (12 arms) containing VAS score data (Figure 10), including Extracorporeal Shock Wave Therapy (ESWT, *n* = 6) and three other conservative treatments: Laser Therapy (LT, *n* = 1), Exercise (EX, *n* = 2), and Placebo (*n* = 3). As shown in the network meta‐analysis results, among the four conservative treatments, Laser Therapy (LT) ranked first in the surface under the cumulative ranking curve (SUCRA) values (SUCRA 100.0%), while ESWT ranked second (SUCRA 75.3%). Importantly, SUCRA reflects only the relative ranking probability of treatment efficacy, and a statistically significant difference in pain reduction was observed between LT and ESWT (ESWT vs. LT, MD 2.55 [95% CI 1.86–3.24]), indicating that LT demonstrated superior efficacy compared to ESWT for VAS outcomes in AT. Additionally, we generated a comparison‐adjusted funnel plot, contribution plot, network forest plot, and funnel plot for bias assessment for this network meta‐analysis, as detailed in the Supporting Information.

**FIGURE 10:**
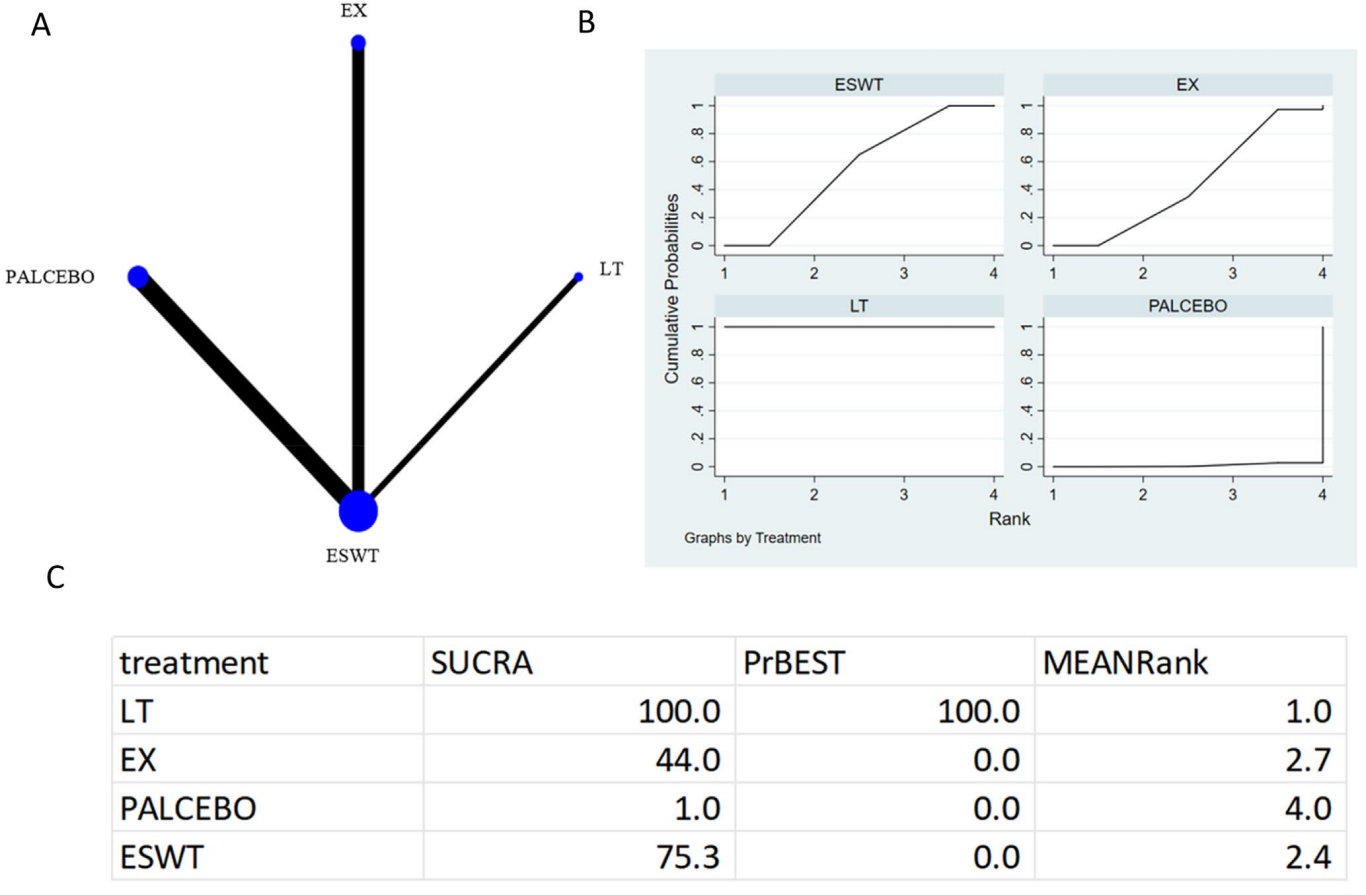
Network diagrams (A), cumulative probability graphs (B), and efficacy ranking table (treatment‐relative ranking) (C) for VAS in Achilles tendinopathy. In the network diagrams (A), node size is proportional to the total number of included participants, and edge thickness is proportional to the number of direct head‐to‐head comparisons between interventions.

#### 3.5.5 Rotator Cuff Tendinosis

*VAS*: A network meta‐analysis was conducted on seven studies (14 arms) containing VAS score data (Figure 11A–C), including Extracorporeal Shock Wave Therapy (ESWT, *n* = 7) and four other conservative treatments: Exercise (EX, *n* = 2), Placebo (*n* = 3), Needle Puncture (NP, *n* = 1), and Mobilization (ML, *n* = 1). As shown in the network meta‐analysis results figure, among the five conservative treatments, ESWT ranked first in the SUCRA curve area (SUCRA 82.3%).

**FIGURE 11:**
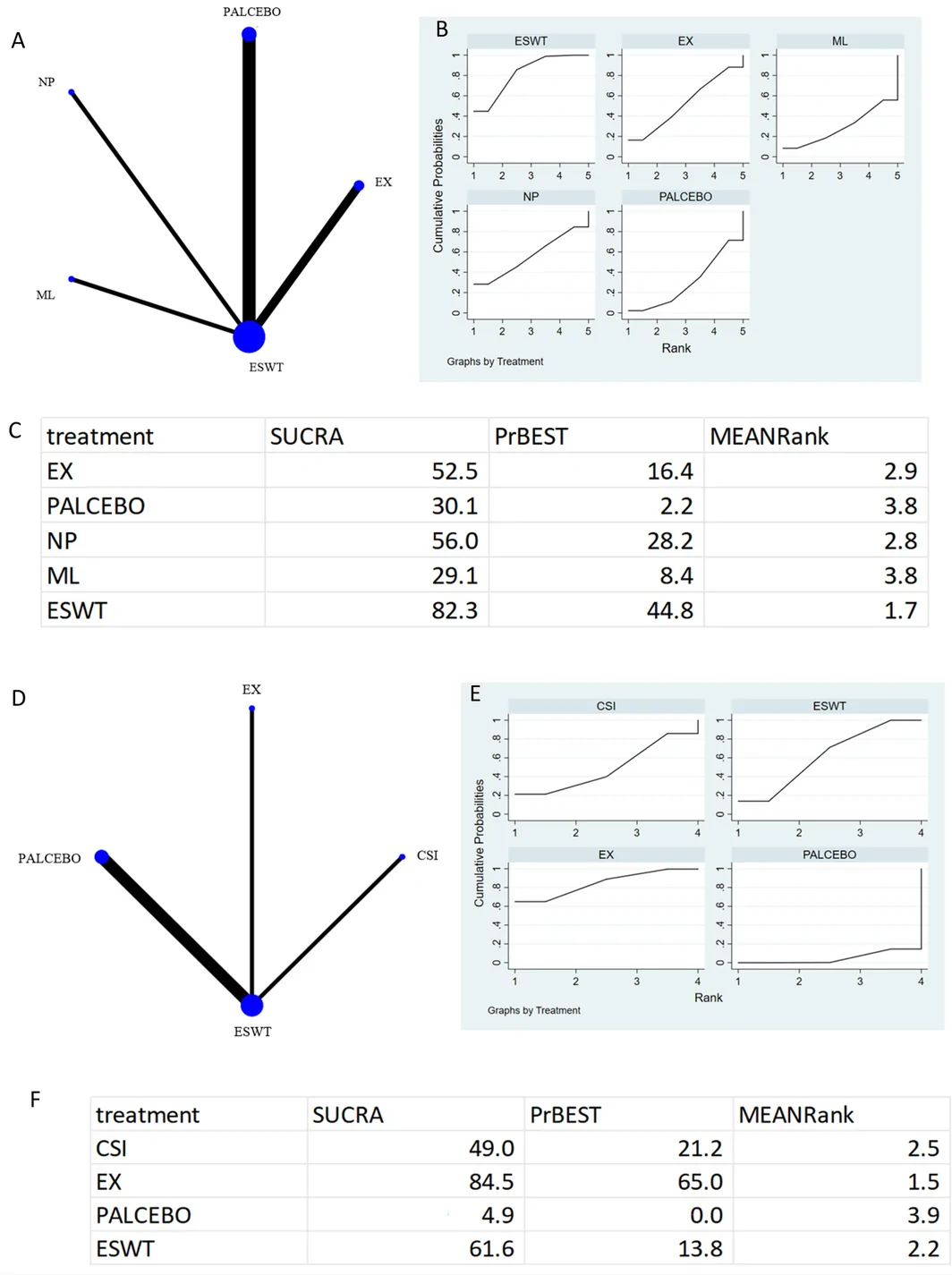
Network diagrams (A), cumulative probability graphs (B), and efficacy ranking table (treatment‐relative ranking) (C) for the VAS in rotator cuff tendinopathy; network diagrams (D), cumulative probability graphs (E), and efficacy ranking table (treatment‐relative ranking) (F) for the SPADI in rotator cuff tendinopathy. In the network diagrams (A), node size is proportional to the total number of included participants, and edge thickness is proportional to the number of direct head‐to‐head comparisons between interventions.

*SPADI*: A network meta‐analysis was conducted on five studies (10 arms) containing SPADI score data (Figure 11D–F), including Extracorporeal Shock Wave Therapy (ESWT, *n* = 5) and three other conservative treatments: corticosteroid injection (CSI, *n* = 1), Exercise (EX, *n* = 1), and Placebo (*n* = 3). As shown in the network meta‐analysis results figure, among the four conservative treatments, ESWT ranked second in the SUCRA curve area (SUCRA 61.6%), following Exercise (SUCRA 84.5%), with no significant difference between them (ESWT vs. EX, MD 0.15 [95% CI −0.24 to 0.55]). Although ranked second, this did not reach statistical significance compared to the first‐ranked intervention.

In summary, in the network meta‐analysis for rotator cuff tendinopathy patients, we found that extracorporeal shock wave therapy showed excellent efficacy in improving VAS scores, ranking first in SUCRA curve area among the included conservative treatments. For improvement in SPADI scores, it ranked second, following exercise therapy.

#### 3.5.6 Greater Trochanter Pain Syndrome (GTPS)

*VAS*: A network meta‐analysis was conducted on two studies (4 arms) containing VAS score data (Figure 12), including Extracorporeal Shock Wave Therapy (ESWT, *n* = 2) and two other conservative treatments: corticosteroid injection (CSI, *n* = 1) and placebo (*n* = 1). As shown in the network meta‐analysis results figure, among the three conservative treatments, ESWT ranked second in SUCRA curve area (SUCRA 54.4%), following CSI (SUCRA 95.6%), with no significant difference between them (ESWT vs. CSI, MD 0.36 [95% CI −0.16 to 0.87]). Although ranked second, this did not reach statistical significance compared to the first‐ranked intervention. Additionally, we generated a comparison‐adjusted funnel plot, contribution plot, network forest plot, and funnel plot for bias assessment for this network meta‐analysis, as detailed in the Supporting Information.

**FIGURE 12:**
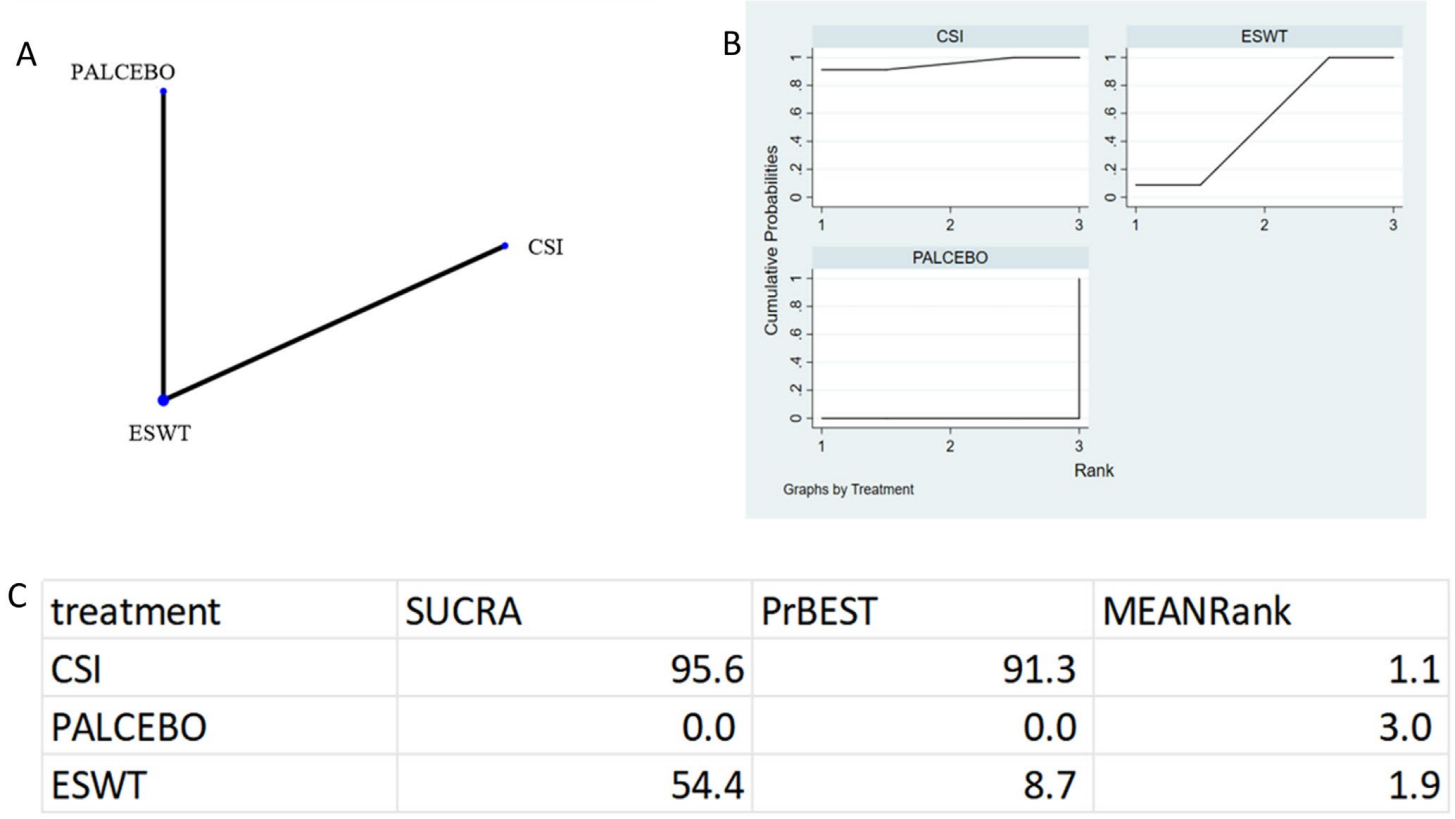
Network diagrams (A), cumulative probability graphs (B), and efficacy ranking table (treatment‐relative ranking) (C) for NES in GTPS. In the network diagrams (A), node size is proportional to the total number of included participants, and edge thickness is proportional to the number of direct head‐to‐head comparisons between interventions.

#### 3.5.7 Publication Bias

For the network meta‐analyses across all groups, we conducted Egger's test and generated funnel plots (in Figures S5 and S8). The results indicated that no significant publication bias was present in any of the network meta‐analyses.

#### 3.5.8 GRADE Evidence Quality Assessment

The certainty of evidence for key NMA comparisons was assessed using the GRADE framework. Overall, the evidence for ESWT versus placebo comparisons across tendinopathy types was rated as moderate quality, with downgrading primarily due to risk of performance bias from lack of blinding in some trials and residual heterogeneity. Evidence for head‐to‐head comparisons between ESWT and other active interventions was generally rated as low to moderate quality, limited by small sample sizes and wide confidence intervals for many comparisons. The complete GRADE evidence summary table is presented in Table S5.

## 4 Discussion

This study is the first to comprehensively analyze the efficacy of Extracorporeal Shock Wave Therapy (ESWT) across various common types of tendinopathies using both pairwise meta‐analysis and network meta‐analysis. It compares the therapeutic effects of ESWT with other conservative treatments within each tendinopathy type. Our findings indicate that ESWT not only provides pain relief for multiple tendinopathy types but also contributes to improving the severity of tendinopathy. Tendinopathy efficacy can be assessed from multiple perspectives, including pain, joint mobility, and imaging studies [1, 96, 97, 98, 99]. Previous meta‐analyses on tendinopathy have predominantly focused on pain indicators, primarily due to challenges in quantifying other indicators or a lack of sufficient RCTs. However, conclusions from such meta‐analyses carry considerable bias and may lack clinical significance. Therefore, this study also includes various scores assessing tendinopathy severity as outcome indicators. These scores provide a multidimensional evaluation of patients, covering aspects such as pain, disability, impact on daily activities, and mental health, as seen with the SPADI score for rotator cuff tendinopathy [26] and the PRTEE score for lateral epicondylitis.

Currently, ESWT is increasingly applied in sports medicine for treating various musculoskeletal injuries, including tendinopathy. Although the precise mechanism of action remains unclear, it is widely believed that ESWT's efficacy may vary across different tendinopathy types. In line with this theoretical background, our study observed substantial heterogeneity when performing an overall pairwise meta‐analysis on all 65 included studies or subgroup analyses based solely on outcome measure scales, driven by mixed tendinopathy subtypes, inconsistent ESWT protocols, and variable control intervention designs across studies. Therefore, the primary analysis method in this study involves subgroup analyses that account for both tendinopathy type and outcome measure scale, comparing ESWT with different control interventions to minimize heterogeneity as much as possible. Another critical source of residual heterogeneity was variation in ESWT treatment parameters, including modality (focused (f‐ESWT) vs. radial (r‐ESWT)), energy flux density, pulse number, and treatment frequency. Focused and radial ESWT differ substantially in wave propagation depth, mechanical stress distribution, and biological effects on tendon tissue, and energy level is a well‐established determinant of ESWT clinical efficacy; these differences likely drive divergent treatment responses across included studies. These two modalities differ in wave propagation depth, mechanical stress distribution, and biological effects on tendon tissue, which may drive divergent treatment responses across included studies. Of note, the cross‐disease pooled NMA presented in Section 3.5.1 is intended for exploratory hypothesis generation only. Given the substantial differences in pathophysiology, natural history, and treatment response across the five included tendinopathy types, this pooled ranking should not be used to guide clinical decisions across different tendinopathy entities. Clinical decision‐making should be based on the disease‐specific NMA results presented in Sections 3.5.2, 3.5.6, which provide more clinically relevant, within‐disease comparative efficacy evidence. Our findings provide actionable, evidence‐based guidance for clinicians to stratify conservative treatment options, prioritize ESWT in appropriate tendinopathy subtypes, and avoid ineffective use in patient populations where it offers no benefit over placebo.

### 4.1 Lateral Epicondylitis (LE)

In the subgroup analysis for lateral epicondylitis (LE), our main conclusion is that ESWT is effective in improving both pain and PRTEE scores in patients compared to placebo, with heterogeneity at 55.8% and 39.0%, respectively. This represents a notable reduction in heterogeneity relative to pre‐subgroup analysis, though some residual heterogeneity remains, likely driven by variable ESWT treatment parameters (frequency, intensity, cycles) and differences in patient baseline disease duration, age, and BMI. This finding aligns with the randomized controlled trial (RCTs) by Rompe et al. [92], which demonstrated that ESWT improved upper limb function and significantly alleviated pain in LE patients. In contrast, the ultrasound and kinesiotaping subgroups showed higher heterogeneity, driven by inconsistent control intervention protocols, limited RCTs numbers, and divergent ESWT regimens across studies.

In the network meta‐analysis for LE, ESWT ranked second in SUCRA values for both pain (VAS) and function (PRTEE) outcomes. Critically, SUCRA only reflects the relative ranking probability of treatment efficacy, not definitive clinical superiority, and no statistically significant difference was found between ESWT and the top‐ranked interventions. However, the therapeutic effect of ESWT in LE remains controversial in published research. Some studies have asserted that ESWT is either ineffective for LE or inferior to other conservative treatments. For instance, Crowther's 2002 prospective clinical study suggested that corticosteroid injections were more effective than ESWT [100], and a 2005 Cochrane review concluded that ESWT had little to no benefit in terms of pain and functional outcomes for LE patients [101]. This discrepancy is largely explained by our inclusion of 18 years of subsequent high‐quality RCTs, standardized subgroup analyses to minimize heterogeneity, and network meta‐analysis of multiple active comparators, all of which were unavailable in the early Cochrane review. We noted that these studies were published relatively early (2005 or earlier), when fewer high‐quality RCTs were available, and lacked a consistent treatment protocol across studies. Additionally, Crowther's study only involved short‐term follow‐up, without considering the long‐term limitations of corticosteroid injections, which weakens the argument against ESWT. As highlighted by Rompe's study [92], high‐quality research on ESWT efficacy for LE requires standardized LE diagnostic criteria (such as MRI), the avoidance of local anesthesia before ESWT, and consistent treatment protocols (targeting the most tender area of the patient). This RCT's findings, showing ESWT's effectiveness surpassing that of placebo, directly contradicted earlier studies. Subsequent high‐quality studies [19, 102] also confirmed that ESWT is effective for LE patients, with advantages over injections in terms of pain relief and grip strength recovery.

Synthesizing these findings, we conclude that ESWT, as a safe non‐invasive treatment, holds clear therapeutic value for LE. Based on its favorable SUCRA ranking and consistent efficacy versus placebo, ESWT may be considered a favorable conservative option in clinical practice, while its head‐to‐head efficacy versus other active interventions requires further validation. Our viewpoint differs from the focus of Angela et al.'s review [2, 103], which described LE as the tendinopathy subtype with the least effective response to ESWT. We argue that a comparison of treatment modalities within the same condition is more meaningful, and therefore, we do not dismiss the therapeutic value of ESWT in LE based on the conclusions of previous studies. Moreover, further research is needed to establish the optimal frequency, energy level, treatment duration, and cycles of ESWT specifically for LE.

### 4.2 Achilles Tendinopathy (AT)

Subgroup analysis revealed that ESWT significantly improved both pain and VISA‐A scores in patients with Achilles tendinopathy (AT) compared to placebo, with no heterogeneity observed in either outcome. However, compared to exercise therapy, ESWT showed no significant advantage in either measure, which aligns with the 2008 clinical study by Rompe et al. [86]. In the network meta‐analysis, ESWT ranked second in SUCRA values for pain outcomes in AT. Consistent with the statistical interpretation above, this relative ranking does not confirm clinical advantage, as a significant efficacy difference was observed between ESWT and the top‐ranked laser therapy. These results are generally consistent with published studies [77, 104], supporting the therapeutic value of ESWT in treating Achilles tendinopathy.

Achilles tendinopathy can be classified anatomically into insertional and non‐insertional types [105], each with distinct characteristics [106]. Non‐insertional AT patients typically experience a more diffuse area of tendon pain, whereas insertional AT patients have a more concentrated pain point [107]. Consequently, during ESWT, insertional AT patients may benefit from more targeted treatment as the shock waves can be directed precisely at the most painful area. Additionally, non‐insertional AT is often associated with more severe ischemia in the mid‐portion of the tendon [107], while ESWT has a relatively modest effect on promoting neovascularization. Based on these theoretical considerations and our meta‐analysis findings, we conclude that ESWT has a confirmed therapeutic value for AT, with more pronounced efficacy for insertional AT. Due to the limited number of RCTs included in this study, we were unable to perform a subgroup analysis based on insertional versus non‐insertional AT. Therefore, the precise therapeutic value of ESWT in AT requires further validation through high‐quality RCTs and meta‐analyses.

### 4.3 Patellar Tendinopathy (PT)

In subgroup analysis, we found that ESWT provided pain relief for patellar tendinopathy (PT) when compared to placebo or ultrasound therapy (with heterogeneity at 0.00% and 69.2%, respectively). The moderate heterogeneity in the placebo subgroup was likely due to variable ESWT dosing and mixed athlete/non‐athlete patient populations across trials. However, for improvements in VISA‐P scores, ESWT showed no significant difference compared to placebo (with 0.00% heterogeneity). In the network meta‐analysis, ESWT ranked second in SUCRA values for both pain and function outcomes in PT. Importantly, this relative ranking cannot be interpreted as clinical benefit, as no significant difference was detected between ESWT and placebo, nor between ESWT and top‐ranked PRP. Our meta‐analysis results indicate that ESWT has no significant therapeutic advantage over placebo for PT, which aligns with current consensus. This conclusion is consistent with the high‐quality systematic review by Korakakis et al. [108], which stated that “there is no short‐ or mid‐term efficacy difference between ESWT and placebo.” While our core findings align, our study extends this work by incorporating network meta‐analysis to compare ESWT against multiple concurrent conservative treatments and further explores patient‐level factors that may modify ESWT response, which were not addressed in the prior review.

Supporting this view, a randomized controlled trial by Karin et al. [73] showed that adding ESWT to eccentric exercise did not significantly enhance efficacy for PT patients. We believe this is associated with the distinct characteristics of PT, or “jumper's knee,” which differs from other tendinopathies. The symptoms of PT are generally not due to acute inflammation but are instead caused by long‐term degenerative changes resulting from repetitive stress, such as jumping, with a pathogenesis of chronic repetitive tendon overload. Consequently, eccentric exercise, which targets this mechanism, is currently recognized as an effective treatment for PT. Nonetheless, we believe that there are still aspects worth exploring regarding the use of ESWT in PT. For example, a study by Williams et al. [109] suggested that MRI should be used to accurately assess intratendinous lesions in PT patients. They found that ESWT is effective for patients without fat pad involvement, while those with fat pad involvement might benefit more from arthroscopic debridement, with ESWT providing no significant effect. Additionally, a review by Molly et al. [96] noted that current placebo treatments often struggle to achieve both effective blinding and complete isolation from the effects of shockwave therapy, which may mean that most placebo treatments do not achieve the expected placebo effect.

In summary, our meta‐analysis supports the conclusion that ESWT has no significant difference in efficacy compared to placebo in PT patients. However, whether ESWT has potential therapeutic value for PT requires further investigation, particularly with studies focusing on specific PT subtypes (e.g., distinguishing athletes from the general population, disease duration, and fat pad involvement) and on the development of precise ESWT treatment protocols to reach definitive conclusions.

### 4.4 Rotator Cuff Tendinopathy (RCT)

In subgroup analysis, we found that ESWT significantly improved SPADI scores and VAS pain scores in patients compared to placebo. Although the subgroup analysis for VAS scores showed high heterogeneity, this cannot be attributed solely to limited RCTs numbers; key drivers include mixed calcific/non‐calcific tendinopathy subtypes and inconsistent ESWT dosage across trials. In the network meta‐analysis, ESWT ranked first in SUCRA for pain outcomes and second for SPADI function scores in RCT, with no significant difference between ESWT and top‐ranked exercise therapy for SPADI. Previously, Xue et al. [110] published the first meta‐analysis and systematic review on the efficacy of ESWT in RCTs, concluding similarly that ESWT had therapeutic effects on shoulder pain and function in RCT patients. However, due to insufficient studies, this research was also unable to conduct subgroup analyses based on shockwave dosage and specific types of RCT.

ESWT can alleviate shoulder pain by stimulating local vasodilation and reducing inflammation through mechanical vibration, as well as releasing adhesions in the affected area [111]. When combined with appropriate exercise training, it may significantly aid in the recovery of shoulder joint mobility, which aligns with our network meta‐analysis results on SPADI scores for RCT patients. Both exercise and ESWT ranked in the top two for SUCRA values, with no significant efficacy difference between them. Although ranked second, this did not reach statistical significance compared to the first‐ranked intervention. We therefore hypothesize that combining ESWT with exercise may yield better pain and functional outcomes for RCT patients, which requires further prospective validation.

Furthermore, the efficacy of ESWT for non‐calcific rotator cuff tendinopathy (NCST) remains controversial. Some studies have reported that ESWT is ineffective for NCST regardless of the energy dosage (EFD) used, without providing a clear explanation for this mechanism [112, 113]. However, a double‐blind randomized controlled trial by Galasso et al. [114] included NCST patients who had failed conservative treatment for at least 4 months and met the clinical criterion of at least 6 months of shoulder pain. Although their study only demonstrated short‐term symptomatic improvement with ESWT, it still holds potential clinical significance. Therefore, although ESWT is more widely accepted for calcific rotator cuff tendinopathy, we believe that, due to its mechanisms of promoting local angiogenesis and stimulating the release of growth factors, ESWT may also provide therapeutic value in NCST [115]. Further high‐quality experimental studies are needed to determine whether the specific dosage for NCST should differ from that used for calcific tendinopathy.

### 4.5 Greater Trochanteric Pain Syndrome (GTPS)

In subgroup and network meta‐analyses, ESWT significantly reduced pain severity in GTPS patients versus exercise training. In network meta‐analysis, CSI ranked first and ESWT second in SUCRA values for pain, with no significant difference between the two treatments, precluding conclusions of superior efficacy. Although ranked second, this did not reach statistical significance compared to the first‐ranked intervention. Consistent with our findings, existing research has shown that isolated gluteal tendon exercise training may be ineffective for GTPS patients [116, 117]. Regarding the comparative efficacy of CSI and ESWT, a previously published meta‐analysis [116, 117] indicated that CSI provides superior short‐term pain relief and functional improvement compared to ESWT. However, CSI may not have an advantage in pain relief and functional improvement over a 2–4 months period. This could be due to corticosteroids further weakening tendon strength in already damaged tendons, leading to reduced collagen synthesis and decreased mechanical properties in tendon cells [116, 117, 118, 119]. However, due to the limited number of RCTs on the application of ESWT in GTPS, with mostly inconsistent outcome measures, only one related meta‐analysis has been published [116], which also showed considerable heterogeneity in its findings. Therefore, more RCTs data are needed to refine the conclusions of this meta‐analysis and provide meaningful guidance for the clinical application of ESWT in GTPS.

### 4.6 Limitation

First, due to the limited number of included studies, our research lacks detailed differentiation and subgroup analyses of specific types of tendinopathies, such as insertional versus non‐insertional Achilles tendinopathy, and calcific versus non‐calcific rotator cuff tendinopathy. Since the effectiveness of ESWT may vary across these tendinopathy types, this limitation may introduce bias into our findings. Additionally, the exploratory cross‐disease NMA pools heterogeneous tendinopathy entities, and its results should be interpreted with extreme caution as noted above. Second, this study did not conduct formal subgroup analyses based on ESWT modality (focused vs. radial) or energy level parameters. A formal subgroup analysis was not feasible due to inconsistent and incomplete reporting of ESWT technical parameters across included studies, with many trials failing to report energy flux density, modality, or complete treatment protocols. Given the inherent differences in physical properties and biological effects between f‐ESWT and r‐ESWT, as well as the established dose–response relationship of ESWT energy level, this omission is likely a key driver of residual heterogeneity across subgroups and could introduce potential bias in the pooled analysis conclusions. Third, although we reduced within‐group heterogeneity through subgroup analysis, a certain degree of heterogeneity remained in each subgroup due to the lack of standardized control measures, as well as the absence of subgroup analyses based on other important variables, such as disease duration, age, BMI, and the presence of other musculoskeletal disorders. Fourth, due to the nature of ESWT, placebo treatments, and exercise therapy, it was challenging to implement blinding for patients, which introduced a certain degree of bias. Fifth, this study included only studies published in English, resulting in some degree of information bias. Lastly, we did not discuss the combined use of multiple conservative treatments, which is common in clinical practice; this omission introduces bias and creates a gap between our analysis conclusions and practical clinical application.

## 5 Conclusion

In this study, pain reduction (assessed by VAS/NRS) was considered the primary outcome measure, while functional improvement (assessed by VISA‐P/A, SPADI, PRTEE) served as the key secondary outcome. Current limited evidence suggests that ESWT is generally effective for pain relief and functional improvement in most common tendinopathies, with variable efficacy across different subtypes. Although ESWT holds a favorable position compared to other conservative treatments across various types of tendinopathies, its therapeutic efficacy may vary depending on the specific type of tendinopathy. Notably, we found that ESWT did not show a significant effect compared to placebo in the treatment of patellar tendinopathy. Through this systematic review, we analyzed the conclusions and limitations of currently published studies, providing insights for future research on the efficacy of ESWT in tendinopathy. Researchers should conduct in‐depth studies on the efficacy differences among various types and doses of shock waves, refine the differentiation of patient‐specific indicators, such as tendinopathy type and disease duration, and investigate the therapeutic value of ESWT in under‐researched types of tendinopathy (e.g., non‐calcific rotator cuff tendinopathy and GTPS). Additionally, exploring the combined use of ESWT with other conservative treatments could help provide personalized ESWT treatment plans for patients in clinical practice, as well as guide decisions regarding the need for combined therapy.

## Author Contributions

**Ning‐Yi Guo:** writing – original draft, visualization, formal analysis, conceptualization. **Wei Liu:** writing – original draft, visualization. **Jian‐quan Wang:** resources, writing – review and editing. **Bing‐bing Xu:** resources, writing – review and editing. **Zi‐Mu Mao:** resources, writing – review and editing. **Si‐Qi Wang:** writing – original draft, conceptualization, visualization, formal analysis.

## Funding

This work was supported by Peking University Third Hospital clinical subject talent project (Y92519‐03); National Natural Science Foundation of China, Grant Nos. 82272571.

## Ethics Statement

The authors have nothing to report.

## Consent

Written informed consent for publication was obtained from all participants.

## Conflicts of Interest

The authors declare no conflicts of interest.

## Supporting information


**Table S1:** PRISMA NMA checklist of items to include when reporting a systematic review involving a network meta‐analysis.
**Table S2:** Characteristics of the included studies.
**Table S3:** Literature search strategies for PubMed, Web of Science, Cochrane Library, and EMBASE.
**Table S4:** List of included studies.
**Table S5:** The GRADE evidence classification of outcomes of tendinopathy in patients treated with ESWT.
**Figure S1:** Risk of bias for each included study.
**Figure S2:** Forest plot for pairwise meta‐analysis of all 65 included studies.
**Figure S3:** Forest plot for pairwise meta‐analysis of 45 studies without come measures on the 0–10 point VAS pain scale.
**Figure S4:** (A) Forest plot for pairwise meta‐analysis of studies using the 0–10 point VAS scale, grouped by control intervention; (B) forest plot for pairwise meta‐analysis of studies using the 0–10 point VAS scale, grouped by tendinopathy type (GTPS, greater trochanteric pain syndrome; PT, patellar tendinopathy; AT, Achilles tendinopathy; LE, lateral epicondylitis; RCT, rotator cuff tendinopathy).
**Figure S5:** Bias assessment for the pairwise meta‐analysis of all 65 included studies: (A) Egger's test, (C) Funnel plot; Bias assessment for the pairwise meta‐analysis of the 45 studies using the 0–10 point VAS pain scale: (B) Egger's test, (D) Funnel plot.
**Figure S6:** Forest plots of eligible comparisons of tendinopathy.
**Figure S6:‐1:** VAS in all types of tendinopathy.
**Figure S6:‐2:** VAS (A) and SPADI (B) in rotator cuff tendinopathy.
**Figure S6:‐3:** VAS (A) and PRTEE (B) in patellar tendinopathy; VAS in Achilles tendinopathy (C); NRS in GTPS (D).
**Figure S6:‐4:** VAS in lateral epicondylitis.
**Figure S6:‐5:** PRTEE in lateral epicondylitis.
**Figure S7:** League table of eligible comparisons of VAS in all kinds of tendinopathy (A), rotator cuff tendinopathy (B), Lateral Epicondylitis (D), Achilles tendinopathy (F), patella tendinopathy (G), GTPS (I); SPADI in rotator cuff tendinopathy (C); PRTEE in Lateral Epicondylitis (E); VISA‐P in patellar tendinopathy (H).
**Figure S8:** Funnel plot of network meta‐analysis for VAS in all kinds of tendinopathy (A), rotator cuff tendinopathy (B), Lateral Epicondylitis (D), Achilles tendinopathy (F), patella tendinopathy (G), GTPS (I); SPADI in rotator cuff tendinopathy (C); PRTEE in Lateral Epicondylitis (E); VISA‐P in patellar tendinopathy (H).
**Figure S9:** Contributions of direct and indirect comparisons to NMA and the number of studies of each direct comparison of VAS in all kinds of tendinopathy (A), rotator cuff tendinopathy (B), Lateral Epicondylitis (D), Achilles tendinopathy (F), patella tendinopathy (G), GTPS (I); SPADI in rotator cuff tendinopathy (C); PRTEE in Lateral Epicondylitis (E); VISA‐P in patellar tendinopathy (H).

## Data Availability

Data sharing not applicable to this article as no datasets were generated or analyzed during the current study.
