# Supplementary material for: The Effect of Extracorporeal Shockwave Therapy in Tendinopathy: A Systematic Review and Network Meta‐Analysis of Randomized Controlled Trials
Source: Orthop Surg. 2026 Sep 13;18(10):2060–82. doi: 10.1111/os.70408 (PMC13572833; doi:10.1111/os.70408)
Supplement: Supplementary file 1 [file OS-18-2060-s001.pdf]

Supplemental Table 1. PRISMA NMA checklist of items to include when reporting a systematic review involving a network meta-analysis.

Section/Topic	Item #	Checklist Item	Reported on Page #
**TITLE**			
Title	1	The effect of extracorporeal shockwave therapy in tendinopathy: A systematic review and network meta-analysis of randomized controlled trials	1
**ABSTRACT**			
Structured summary	2	Provide a structured summary including, as applicable:<br>**Background:** main objectives<br>**Methods:** data sources; study eligibility criteria, participants, and interventions; study appraisal; and *synthesis methods, such as network meta-analysis.*<br>**Results:** number of studies and participants identified; summary estimates with corresponding confidence/credible intervals; *treatment rankings may also be discussed. Authors may choose to summarize pairwise comparisons against a chosen treatment included in their analysesfor brevity.*<br>**Discussion/Conclusions:** limitations; conclusions and implications of findings.<br>**Other:** primary source of funding; systematic review registration number with registry name.	1
**INTRODUCTION**			
Rationale	3	Describe the rationale for the review in the context of what is already known*, including mention of why a network meta-analysis has been conducted.*	1-2
Objectives	4	Provide an explicit statement of questions being addressed, with reference to participants, interventions, comparisons, outcomes, and study design (PICOS).	2
**METHODS**			
Protocol and registration	5	Indicate whether a review protocol exists and if and where it can be accessed (e.g., Web address); and, if available, provide registration information, including registration number.	3
Eligibility criteria	6	Specify study characteristics (e.g., PICOS, length of follow-up) and report characteristics (e.g., years considered, language, publication status) used as criteria for eligibility, giving rationale. *Clearly describe eligible treatments included in the treatment network, and note whether any have been clustered or merged into the same node (with justification).*	3

Information sources	7	Describe all information sources (e.g., databases with dates of coverage, contact with study authors to identify additional studies) in the search and date last searched.	3
Search	8	Present full electronic search strategy for at least one database, including any limits used, such that it could be repeated.	4
Study selection	9	State the process for selecting studies (i.e., screening, eligibility, included in systematic review, and, if applicable, included in the meta-analysis).	4
Data collection process	10	Describe method of data extraction from reports (e.g., piloted forms, independently, in duplicate) and any processes for obtaining and confirming data from investigators.	4
Data items	11	List and define all variables for which data were sought (e.g., PICOS, funding sources) and any assumptions and simplifications made.	4
Geometry of the network	S1	Describe methods used to explore the geometry of the treatment network under study and potential biases related to it. This should include how the evidence base has been graphically summarized for presentation, and what characteristics were compiled and used to describe the evidence base to readers.	4
Risk of bias within individual studies	12	Describe methods used for assessing risk of bias of individual studies (including specification of whether this was done at the study or outcome level), and how this information is to be used in any data synthesis.	4
Summary measures	13	State the principal summary measures (e.g., risk ratio, difference in means). Also describe the use of additional summary measures assessed, such as treatment rankings and surface under the cumulative ranking curve (SUCRA) values, as well as modified approaches used to present summary findings from meta-analyses.	4
Planned methods of analysis	14	Describe the methods of handling data and combining results of studies for each network meta-analysis. This should include, but not be limited to:<br>• Handling of multi-arm trials;<br>• Selection of variance structure;<br>• Selection of prior distributions in Bayesian analyses; and<br>• Assessment of model fit.	4-5
Assessment of Inconsistency	S2	Describe the statistical methods used to evaluate the agreement of direct and indirect evidence in the treatment network(s) studied. Describe efforts taken to address its presence when found.	5

Risk of bias across studies	15	Specify any assessment of risk of bias that may affect the cumulative evidence (e.g., publication bias, selective reporting within studies).	5
Additional analyses	16	Describe methods of additional analyses if done, indicating which were pre-specified. This may include, but not be limited to, the following:<br>• Sensitivity or subgroup analyses;<br>• Meta-regression analyses;<br>• *Alternative formulations of the treatment network; and*<br>• *Use of alternative prior distributions for Bayesian analyses (if applicable).*	5
**RESULTS**			
Study selection	17	Give numbers of studies screened, assessed for eligibility, and included in the review, with reasons for exclusions at each stage, ideally with a flow diagram.	5
Presentation of network structure	S3	Provide a network graph of the included studies to enable visualization of the geometry of the treatment network.	5
Summary of network geometry	S4	Provide a brief overview of characteristics of the treatment network. This may include commentary on the abundance of trials and randomized patients for the different interventions and pairwise comparisons in the network, gaps of evidence in the treatment network, and potential biases reflected by the network structure.	5
Study characteristics	18	For each study, present characteristics for which data were extracted (e.g., study size, PICOS, follow-up period) and provide the citations.	5
Risk of bias within studies	19	Present data on risk of bias of each study and, if available, any outcome level assessment	6
Results of individual studies	20	For all outcomes considered (benefits or harms), present, for each study: 1) simple summary data for each intervention group, and 2) effect estimates and confidence intervals. *Modified approaches may be needed to deal with information from larger networks.*	6-18
Synthesis of results	21	Present results of each meta-analysis done, including confidence/credible intervals. *In larger networks, authors may focus on comparisons versus a particular comparator (e.g., placebo or standard care), with full findings presented in an appendix. League tables and forest plots may be considered to summarize pairwise*	6-18

		comparisons. If additional summary measures were explored (such as treatment rankings), these should also be presented.	
Exploration for inconsistency	S5	Describe results from investigations of inconsistency. This may include such information as measures of model fit to compare consistency and inconsistency models, *P* values from statistical tests, or summary of inconsistency estimates from different parts of the treatment network.	6-18
Risk of bias across studies	22	Present results of any assessment of risk of bias across studies for the evidence base being studied.	18
Results of additional analyses	23	Give results of additional analyses, if done (e.g., sensitivity or subgroup analyses, meta-regression analyses*, alternative network geometries studied, alternative choice of prior distributions for Bayesian analyses,* and so forth).	18
**DISCUSSION**			
Summary of evidence	24	Summarize the main findings, and the mechanisms by which various intervention measures achieve therapeutic efficacy are extensively discussed, alongside unresolved issues in current research.	18-22
Limitations	25	Discuss limitations at study and outcome level (e.g., risk of bias), and at review level (e.g., incomplete retrieval of identified research, reporting bias). *Comment on the validity of the assumptions, such as transitivity and consistency. Comment on any concerns regarding network geometry (e.g., avoidance of certain comparisons).*	22
Conclusions	26	Provide a general interpretation of the results in the context of other evidence, and implications for future research.	22

PICOS = population, intervention, comparators, outcomes, study design.

* Text in italics indicate wording specific to reporting of network meta-analyses that has been added to guidance from the PRISMA statement.

† Authors may wish to plan for use of appendices to present all relevant information in full detail for items in this section.

Supplemental Table 2 Characteristics of the included studies

Author	Year	Disease	Numner of samples Total(I/C)	Sex(M/F)	Age	Intervention	Control	Outcome Indicators	Follow up
Sen SB	2024	LE	50(25/25)	I(9/16);C(12/13)	I(47.08 ± 6.05)；C(45.40 ± 8.54)	ESWT 2000 impulses at 2 bar at 12Hz 3 sessions (weekly interval)	output power:15 W frequency:15 Hz energy density:39 J/cm2 15 consecutive sessions over the course of 3 weeks	Q-DASH	baseline、3w、12w
Wajida Perveen	2024	LE	74(35/39)	I(11/24);C(11/28)	I(47.83 ± 9.10)；C(47.36 ± 7.02)	ESWT 2000 impulses at 2.0mJ/mm2 at 2 bar at 1Hz (Each session lasted for 5 min, a total of 7 sessions with a 4-day interval)	US:at a rate of 2.5w/sec2 for 3–5 min,three times per week for a total of seven weeks. Deep friction massage:2.3 kg/cm2	PRTEE	baseline、3w、7w
Heaver, C	2023	GTPS	95(48/47)	I(6/42);C(4/43)	I(63.73±11.87)；C(60.31±12.74)	f-ESWT 2500 impulses at 0.15–0.35mJ/mm2 3 sessions (weekly interval)	80mg of Depo Medrone with 3.5ml 0.5% bupivic aine and 3.5ml 1% lignocaine were combined in a single syringe and injected using a long 21G (green) needle under ultrasound guidance	VAS、HHS、SF-36	baseline、3m、12m
Yagci, H. C	2023	GTPS	60(32/28)	I(5/27);C(7/21)	I(50.0±9.1)；C(51.7±7.7)	r-ESWT 2000 impulses at 2 bar at 12Hz 3 sessions (weekly interval)	1 mL of 40 mg triamcinolone acetonide and 2 mL of 2% prilocaine hydrochloride (20 mg/mL), 5-cm-long 23-gauge needle	VAS、SF-36、WOMAC	baseline、3w、3m
Notarnicola, A	2023	GTPS	30(15/15)	I(3/12);C(2/13)	I(59.1±9.6)；C(59.5±7.7)	f-ESWT 2000 impulses at 0.03–0.17mJ/mm2 at 4 Hz 3 sessions (weekly interval)	Eccentric therapeutic exercise: Piriformis muscle stretching;gluteal muscle stretching; wall squat with a ball; leg lift;iliotibial band stretching (5 days a week and a total of 4 weeks of treatment)	NRS、RMS	baseline、2m、4m、6m
Stania M	2023	AT	26(13/13)	I(11/2);C(9/4)	I(42±11.42)；C(36.69±11.57)	r-ESWT 2000 impulses at 0.03–0.17mJ/mm2 at 10Hz at 3 bar 3 sessions (weekly interval)	ultrasound frequency 3 MHz; ultrasound power density 1.0 W/cm2; pulse ratio 50% 10 treatments during 2 weeks.	VAS	1w、6w

Volkan Sah	2023	PT	78(26/28/24)	I1(11/15);I2(13/15);C(10/14)	I1(26.22±5.57)；I2(24.76±4.58)C(26.55±5.10)	I1：<br>f-ESWT:part 1 (5 Hz, 1.6 Bar, 500 pulses, 0.02-0.60 mJ/mm2, 100 seconds) + part 2 (8 Hz, 1.8 Bar, 1800 pulses, 0.02-0.60 mJ/mm2,225 seconds)<br>I2：<br>r-ESWT:part 1 (5 Hz, 1.4 Bar, 1400 pulses, 0.168 mJ/mm2, 280 seconds) + part 2 (8 Hz, 1.5 Bar, 1800 pulses, 0.180 mJ/mm2, 225 seconds).<br>(3 sessions, weekly interval)	palcebo of ESWT	VAS、VISA-P	baseline、3w、7w、15w
Kaplan, S.	2023	LE	87(30/29/28)	I1(14/16);I2(14/15);C(13/15)	I1(49.3±14.0)；I2(44.2±13.3)C(46.0±16.9)	I1：<br>f-ESWT:part 1 (4Hz, 1.5 Bar, 500 pulses, 0.02-0.60 mJ/mm2, 125 seconds) + part 2 (8 Hz, 1.7 Bar, 500 pulses, 0.144 mJ/mm2,225 seconds)<br>I2：<br>r-ESWT:part 1 (4Hz, 1.2 Bar, 500 pulses, 0.144 mJ/mm2, 125 seconds) + part 2 (8 Hz, 1.5 Bar, 1800 pulses, 0.180 mJ/mm2, 225 seconds).<br>(3 sessions, 2-4d interval)	palcebo of ESWT	PRTEE	baseline、5w、13w
Kim, J.	2023	RCT	26(13/13)	I(5/8);C(3/9)	I(52.31±7.25)；C(53.00±9.52)	f-ESWT 2000 impulses at 0.142mJ/mm2<br>3 sessions (weekly interval)	triamcinolone acetonide; 40 mg/m/V, 25-G needle	VAS	1m、3m
Ebadi, Safoora	2023	RCT	33(17/16)	I(7/10);C(5/11)	I(53.29±10.71)；C(55.94±5.29)	r-ESWT 2000 impulses at 5Hz at 4 bar<br>3 sessions (weekly interval)	1cc of triamcinolone 40 mg per ml with 2cc lidocaine 1% (3cc in total) ,23-gauge needle	VAS、Q-DASH	baseline、2w、12w
ElGendy, M. H	2023	RCT	60(20/20/20)	/	I(31.94±4.66)；C1(30.47±3.69)；C2(32.72±4.38)	ESWT 2000 impulses at 6Hz at 0.2-0.3mJ/mm2<br>3 sessions (weekly interval)	Contorl1： local corticosteroid injection of 40 mg triamcinolone acetonide mixed with 1% xylocaine<br>Control2：palcebo	SPADI	baseline、4w、12w
Wheeler, P. C	2022	GTPS	120(57/63)	I(8/49);C(15/48)	I(61.7±12.3)；C(60.0±10.8)	r-ESWT 2000 impulses at 20 Hz<br>3 sessions (weekly interval)	palcebo of ESWT	NRS、RMS	baseline、6w、3m、6m

Benli, M. D.	2022	AT	63(31/32)	I(9/22);C(14/18)	I(40.0±10.5)；<br>C(34.8±13.3)	r-ESWT 1500 impulses at 12 Hz at 2 bar(midportion Achilles tendon)+r-ESWT 2000 impulses at 15 Hz at 1.8 bar(gastrocnemius-soleus muscle)<br>4 sessions (weekly interval)	Eccentric exercises：3 sets x 15 repetitions, twice per day for three months at home	VAS、VISA-A	baseline、3m、2y
Fatima, A.	2022	RCT	40(20/20)	/	I(48.7±6.74)；<br>C(49.8±7.54)	r-ESWT 2000 impulses at 120 Hz at 0.03-0.32mJ/mm2,<br>15-20 minutes each session,twice a week for six weeks	palcebo	NRS、CMS	baseline、6w、12w
Aldajah, S.	2022	LE	40(20/20)	I(11/9);C(10/10)	I(42.0±7.30)；<br>C(42.37±6.69)	ESWT 2000 impulses at 16 Hz at 1.6 bar<br>3 sessions (weekly interval)	Physical therap(1 min friction massage, followed by 3 min of continuous therapeutic ultrasound with a frequency of 1.5 Hz)	VAS、DASH	baseline、post-treatment
Nambi, G	2022	LE	60(30/30)	/	I(47.12±3.2)；<br>C(46.56±4.1)	ESWT 2000 impulses at 8Hz at 2.5 bar<br>4 sessions (weekly interval)	palcebo of ESWT	VAS、PRTEE	baseline、4w、8w、6m
Kuo, Y. C.	2022	RCT	41(20/21)	I(8/12);C(8/13)	I(57.6±9.4)；<br>C(58.2±7.9)	r-ESWT 2000 impulses at 2Hz at 0.26mJ/mm2,<br>3 sessions (weekly interval)	ultrasound guided needle puncture(USNP)：<br>3.8-cm-long 22-gauge needle attached to a 5-mL syringe<br>was used for puncturing	VAS、SF-36	baseline、1.5m、3m
Mansur, N	2021	AT	119(58/61)	I(31/27);C(30/31)	I(52.2±10.9)；<br>C(53.5±11.4)	r-ESWT 2000-3000 impulses at 7-10Hz at 1.5-2.5 bar, 3 sessions (2 weeks interval)	Eccentric exercise: twice a day, 7 days per week, for 3 consecutive months	VAS、VISA-A	baseline、2w、4w、6w、12w、24w
Abdelkader, N. A	2021	AT	50(25/25)	/	I(29.9±7.0)；<br>C(28.3±6.8)	r-ESWT 2000 impulses at 8Hz at 3 bar at 0.26mJ/mm2,<br>4 sessions (weekly interval)	palcebo of ESWT	VISA-A	baseline、post-treatment(1m)、16m
Özmen, T	2021	LE	40(14/13/13)	I(4/10);C1(5/8);C2(7/6)	I(48.36±11.51)；<br>C1(47.15±9.87):C2<br>(49.62±10.20)	r-ESWT 1500 impulses at 4Hz at 1.4 bar at 0.22mJ/mm2,<br>3 sessions for 2 weeks	C1:KT once every 2 days for 2 weeks<br>using muscle and fascia correction techniques<br>C2：US frequency of 1 MHz, intensity of 1 W/cm2 for 3 min , 5 days a week for 2 weeks	VAS、PRTEE	baseline、2w、8w

Sahbaz, T.	2021	LE	74(25/25/24)	I(4/21);C1(5/20);C2(5/19)	I(49.2±6.4)；C1(46.3±6.8):C2(48.0±9.1)	r-ESWT 2000 impulses at 10Hz at 1.8 bar<br>3 sessions (weekly interval)	C1：PRP (20 mL venous blood, 3 mL PRP was injected)<br>C2;palcebo	DASH、PRTEE	baseline、1m、2m、3m、6m
Shim, B. J	2021	LE	63(21/21/21)	I(9/12);C1(13/8);C2(11/10)	I(48.7±7.1)；C1(52.2±8.2):C2(52.3±10.2)	ESWT 500 impulses at 4Hz at 1.5 bar	C1：PDRN 5.625 mg/3 mL<br>C2：palcebo	VAS	baseline、12w
Moretti, L	2021	LE	30(15/15)	/	/	ESWT 1600 impulses at 4Hz at 0.01-010mJ/mm2,<br>3 sessions (weekly interval)	PRP：<br>2 ml CB-PRP along with 1 ml of 2% lidocaine	VAS、Q-DASH	baseline、3m、6m
Ramon, S	2020	GTPS	103(53/50)	I(9/42);C(18/32)	I(57.1±12.9)；C(55.6±11)	f-ESWT 2000 impulses at 0.20mJ/mm2 at 5Hz<br>3 sessions (weekly interval)	palcebo of ESWT	VAS、HHS	baseline、1m、2m、3m、6m
Pinitkwamdee, S.	2020	AT	31(16/15)	I(2/14);C(5/10)	I(61.4±5.9)；C(56.5±7.9)	r-ESWT 2000 impulses at 8-12Hz at 2.5-3.5 bar at 0.12-0.16 mJ/mm2<br>4 sessions (weekly interval)	palcebo of ESWT	VAS	baseline、2w、3w、4w、6w、12w、24w
Lee, W. C	2020	PT	30(16/14)	I(16/0);C(14/0)	I(21.1±2.2)；C(24.1±4.6)	r-ESWT 1500 impulses at 8-4Hz at 0.08 mJ/mm2<br>6 sessions (weekly interval)	palcebo of ESWT	VAS、VISA-P	baseline、1w
Turgay, T	2020	LE	52(26/26)	I(14/12);C(10/16)	I(48±10)；C(48±11)	r-ESWT 2000 impulses<br>5 sessions (weekly interval)	LLLT：<br>905 nm wavelength；5 min over the six trigger points of<br>the lateral extensor group；dose：0.25-1,2；<br>15 sessions on consecutive days	PRTEE、DASH、SF-36	baseline、post-treatment
Guler, T	2020	LE	40(20/20)	I(6/14);C(7/13)	I(44.8±8.7)；C(40.5±7.9)	ESWT<br>3 sessions (weekly interval)	KT:3-week treatment,<br>5 days a week	VAS、Q-DASH、RMS	baseline、4w、8w

Notarnicola, A	2020	RCT	30(15/15)	/	/	ESWT 2000 impulses at 0.05 mJ/mm2<br>3 sessions (weekly interval)	12 weeks of therapeutic exercise for strengthening the rotator cuff and shoulder mobility	VAS	baseline、3m
Atalay, S. G.	2020	LE	76(38/38)	I(13/25);C(13/25)	I(45,8±7,60)；<br>C(43,8±11,1)	r-ESWT 2000 impulses at 0.08-0.28 mJ/mm2 at 10 Hz at 1.9 bar<br>3 sessions (weekly interval)	Neural therapy：<br>16 mL of local anesthetic (5 mg/mL lidocaine HCL) was injected in each session；<br>from C4 to T8 for each spinous process and 0.5–2 cm laterally on the affected side (a total of 26 injection areas)	VAS	baseline、post-treatment、12w
Carlisi, E.	2019	GTPS	50(26/24)	I(5/21);C(2/22)	I(61±9.18)；<br>C(61.5±9.52)	ESWT 1800 impulses at 3 bar at 0.15mJ/mm2<br>3 sessions (weekly interval)	1.5W/cm2 in sessions of<br>10minutes each<br>daily for 10 consecutive days	NRS	baseline、2m、6m
Cheng, L.	2019	PT	51(26/25)	I(12/14);C(13/12)	I(22.7±5.3)；<br>C(21.9±4.6)	r-ESWT 2000 impulses at 12Hz at 1.5-3.0 bar<br>16 sessions (weekly interval)	acupuncture (acupuncture in pain spot), ultrasonic wave therapy (Chatt2776 USA; treatment time, 8 min; intensity, ~0.8–1.2 W/cm2), and microwave therapy (Fysiomed, Edegem, Belgium; treatment time, 20 min; strength, ~90–100 W).<br>16 sessions (weekly interval)	VAS	baseline、16w
Celik, D	2019	LE	43(20/23)	I(5/15);C(6/17)	I(48.0±9.9)；<br>C(48.2±9.4)	f-ESWT 2000 impulses at 0.09mJ/mm2<br>4 sessions (weekly interval)	photobiomodulation therapy (PBMT):<br>wavelength mode was set at 904 nm, the frequency level at 50 Hz, the powerintensity on the skin at 40 mW, the spot<br>size at 0.5 cm2<br>, 50% was the cycle of the duty, and the energy density at 2.4 J/cm2.<br>three times a week for 4 weeks	VAS、DASH	baseline、post-treatment、12w
Ahadi T	2019	LE	33(16/17)	I(4/12);C(6/11)	I(47.25)；C(46.65)	ESWT 2000 impulses at 1.5 bar at 10Hz<br>3 sessions in 1 week	prolotherapy：<br>2cc of lidocaine 2%, under aseptic conditions and using a 25-gauge 1.5-inch needle, 3cc of dextrose 20% was injected deeply, with the needle touching bone	VAS、Q-DASH	baseline、4w、8w

Duymaz, T	2019	RCT	80(40/40)	/	I(54.33±9.88)；<br>C(51.31±8.86)	r-ESWT 1500 impulses at 0.28 mJ/mm2<br>4 sessions (weekly interval)	palcebo	VAS、Q-DASH	baseline、<br>post-treatment
Atya, A. M.	2019	RCT	60(20/20/20)	/	I(45.8±7.4)；<br>C1(41.5±5.8)；<br>C2(45.2±7.3)	r-ESWT 1500 impulses at 0.11 mJ/mm2 at 10 Hz at 2.5-4.0 bar<br>4 sessions (weekly interval)	C1：mobilization<br>C2：palcebo	VAS	baseline、<br>post-treatment
Vahdatpour, B	2018	AT	43(22/21)	I(4/18);C(4/17)	I(54.9±11.3)；<br>C(54.3±12.4)	f-ESWT 1500 impulses at 0.25-0.40 mJ/mm2 at 2.3 Hz<br>+r-ESWT 3000 impulses at 1.8-2.6 mJ/mm2 at 2.21 Hz , 4 sessions (weekly interval)	palcebo of ESWT	VAS	baseline、<br>post-treatment、<br>4w、16w
Yalvaç, B	2018	LE	44(20/24)	I(5/15);C(8/16)	I(46.04±9.24)；<br>C(43.75±4.52)	ESWT 2000 impulses at 10-15 Hz at 1.5-2.5 bar<br>3 sessions (weekly interval)	therapeutic US：<br>1 cm2 application area, at 1.5 W/cm2 , 1 MHz frequency, continuous mode in painful area, 5 min once a day, 5 days a week,for 10 sessions in total	PRTEE、SF-36	baseline、<br>post-treatment、<br>1m
Eraslan, L	2018	LE	45(15/15/15)	/	I(48±5.7)；<br>C1(48.5±5.6)；<br>C2(47.2±8.5)	ESWT 2000 impulses at 0.06-0.12 mJ/mm2<br>3 sessions (weekly interval)	C1：taped 5 days a week for 3 weeks using muscle and fascia correction techniques<br>C2：palcebo	VAS、PRTEE	baseline、<br>post-treatment
Aydin, A.	2018	LE	67(32/35)	I(15/17);C(19/16)	I(38.84±6.77)；<br>C(37.94±6.45)	ESWT 2000 impulses at 10-12 Hz at 1.6-1.8 bar<br>4 sessions in 1 week	wrist splint,<br>holding the wrist at 30°–45° extension for 4 weeks.	VAS、PRTEE、<br>SF-36	baseline、4w、<br>12w、24w
Guler, N. S.	2018	LE	40(20/20)	I(6/14);C(6/14)	I(46.3±8.09)；<br>C(45.8±10.8)	ESWT 1500 impulses at 21 Hz at 1.8 bar	palcebo of ESWT	VAS、PRTEE	baseline、<br>post-treatment

Kvalvaag, E	2018	RCT	143(69/74)	I(37/32);C(33/41)	I(46.0±10.9)；<br>C(47.6±9.9)	r-ESWT 2000 impulses at 1.5-3 bar at 0.01-0.35 mJ/mm2<br>4 sessions (weekly interval)	palcebo of ESWT	NRS、SPADI	baseline、1y
Thijs, K. M	2017	PT	52(22/30)	I(8/14);C(6/24)	I(30.5±8.0)；<br>C(27.3±5.2)	f-ESWT 1000 impulses at 0.2 mJ/mm2 at 4Hz<br>3 sessions (weekly interval)	palcebo of ESWT	NRS、VISA-P	baseline、6w、12w、24w
Yang, T. H	2017	LE	28(15/13)	I(8/7);C(4/9)	I(50.93±8.40)；<br>C(51.08±9.52)	r-ESWT 2000 impulses at 10Hz<br>3 sessions (weekly interval)	palcebo of ESWT	VAS、DASH	baseline、6w、12w、24w
Capan, N	2016	LE	45(23/22)	I(7/16);C(3/19)	I(48.4±9.0)；<br>C(46.2±7.4)	r-ESWT 2000 impulses at 10Hz at 1.8 bar<br>3 sessions (weekly interval)	palcebo of ESWT	VAS、PRTEE	baseline、1m、3m
Lizis, P.	2015	LE	50(25/25)	/	I(47.9±4.4)；<br>C(49.0±4.5)	ESWT 1000/1500/2000 impulses at 8Hz at 2.5 bar at 0.4 mJ/mm2<br>5 sessions (weekly interval)	ntensity, 0.8 W/cm2; 100% fill; carrier frequency, 1 MHz.<br>10 treatments 3 times per week	VAS	baseline、post-treatment、3m
Notarnicola, A.	2014	AT	60(30/30)	/	I(59.5 ± 11.9)；<br>C(57.5 ± 14.6)	ESWT 1600 impulses at 0.05-0.07 mJ/mm2<br>3 sessions (3-4 days interval)	CHELT：<br>ten daily sessions of 1,200 J and 12 W of laser therapy (wavelength of 1,084, 810 and 980 nm) added to a flow of cold air at −30 °C.	VAS、RMS	baseline、post-treatment、2m、6m
Devrimsel, G.	2014	LE	60(30/30)	I(8/22);C(10/20)	I(37.76±8.52)；<br>C(40.30±10.00)	r-ESWT 2000 impulses at 16Hz at 1.6 bar<br>3 sessions (weekly interval)	low dose-regimen laser therapy with 3.6 joule intensity, 500 Hz fre quency, and 850 nm wavelength, which was applied for 40 sec onds in each session	VAS	baseline、4w、12w

Efe, T.	2014	RCT	29(15/14)	/	/	r-ESWT 2000 impulses at 0.11 mJ/mm2<br>3 sessions (weekly interval)	palcebo of ESWT	VAS、DASH	10y
Vetrano, M	2013	PT	46(23/23)	I(17/6);C(20/3)	I(26.8±8.5)；<br>C(26.9±9.1)	f-ESWT 2400 impulses at 0.17-0.25 mJ/mm2<br>3 sessions at 48- to 72-hour intervals	PRP：<br>1 injection per week for 2 weeks	VAS、VISA-P	baseline、2m、6m、12m
Sarkar, B	2013	LE	30(15/15)	I(7/8);C(5/10)	I(41.9±4.5)；<br>C(42.9±5.3)	ESWT 2000 impulses at 0.06 mJ/mm2<br>3 sessions (weekly interval)	a supervised exercise protocol three times a week.	VAS、DASH	baseline、post-treatment
Zwerver, J	2011	PT	62(31/31)	I(20/21);C(21/10)	I(24.2±5.2)；<br>C(25.7±4.5)	ESWT 2000 impulses at 0.58 mJ/mm2 at 4 Hz<br>3 sessions (weekly interval)	palcebo of ESWT	VAS、VISA-P	baseline、1w、12w、22w
Engebretsen, K	2011	RCT	97(48/49)	/	/	r-ESWT 2000 impulses at 2.5-4.0 bar at 8-12 Hz<br>4-6 sessions (weekly interval)	two 45-minute sessions weekly for a maximum of 12 weeks	VAS、SPADI	baseline、1y
Rompe, J. D.	2009	GTPS	229(75/76/78)	I(21/54);C1(23/53);C2(23/55)	I(50)；C1(46)；<br>C2(47)	ESWT 2000 impulses at 3 bar at 0.12mJ/mm2<br>3 sessions (weekly interval)	C1：exercise<br>C2：CSI	NRS	baseline、1m、4m、15m
Rompe, J. D.	2009	AT	68(34/34)	I(16/18);C1(14/20);C2(23/55)	I(53.1±9.6)；<br>C(46.2±10.2)	r-ESWT 2000 impulses at 3 bar at 0.1mJ/mm2 at 8 Hz, 3 sessions (weekly interval)	palcebo	NRS、VISA-A	baseline、4m
Rompe, J. D	2008	AT	50(25/25)	I(9/16);C(11/14)	I(39.2±10.7)；<br>C(40.4±11.3)	r-ESWT 2000 impulses at 2.5 bar at 0.12mJ/mm2 at 8 Hz<br>3 sessions (weekly interval)	exercise	NRS、VISA-A	baseline、4m

Staples, M. P.	2008	LE	63(33/30)	I(19/14);C(18/12)	I(49.8±7.4)；C(49.1±8.8)	ESWT at 0.56mJ/mm2<br>3 sessions (weekly interval)	palcebo of ESWT	VAS、SF-36	baseline、6w、3m、6m
Rompe, J. D	2007	AT	75(25/25/25)	I(11/14);C1(9/16);C2(9/16)	I(48.1±9.9)；C1(51.2±10.3)；C2(46.4±11.4)	r-ESWT 2000 impulses at 3 bar at 0.1mJ/mm2 at 8 Hz<br>3 sessions (weekly interval)	C1：exercise<br>C2：palcebo	NRS、VISA-A	baseline、4m
Wang, C. J.	2007	PT	54(30/24)	I(14/13);C(13/10)	I(29.4±10.5)；C(30.2±10.4)	r-ESWT 1500 impulses at 0.18mJ/mm2<br>1 session	nonsteroidal anti-inflammatory drugs, physiotherapy, exercise program, and the use of a knee strap	VAS、VISA-P	baseline、post-treatment
Costa, M. L	2005	AT	49(22/27)	I(9/13);C(12/15)	I(58.7±10.8)；C(47.7±13.5)	f-ESWT 1500 impulses at 0.2mJ/mm2<br>3 sessions (monthly interval)	palcebo of ESWT	VAS	baseline、1y
Pettrone, F. A.	2005	LE	114(56/58)	/	/	f-ESWT 2000 impulses at 0.06mJ/mm2	palcebo of ESWT	VAS	baseline、12w
Rompe, J. D.	2004	LE	70(34/36)	I(18/16);C(16/18)	I(45.9±12.3)；C(46.2±11.2)	ESWT 2000 impulses<br>3 sessions (weekly interval)	palcebo of ESWT	VAS、RMS	baseline、3m、12m
K. Taunton	2003	PT	20(10/10)	I(5/5);C(5/5)	/	ESWT 2000 impulses at 0.17mJ/mm2<br>3-5 sessions (monthly interval)	palcebo of ESWT	VISA-P	baseline、5w、12w
Speed, C. A	2002	LE	75(40/35)	I(19/21);C(14/21)	I(46.5)；C(48.2)	ESWT 1500 impulses at 0.18mJ/mm2<br>3 sessions (monthly interval)	palcebo of ESWT	VAS	baseline、1m、2m、3m
Speed, C. A	2002	RCT	74(37/37)	/	/	ESWT 1500 impulses at 0.12mJ/mm2<br>3 sessions (monthly interval)	palcebo of ESWT	VAS、SPADI	baseline、1m、3m

Supplemental Table 3 Literature Search Strategies for PubMed, Web of Science, Cochrane Library, and EMBASE

No.	Pubmed 152 results (MESH Terms)	Embase 422 results (Emtree Terms)	Cochrane 128 results (MESH Terms)	Web of science 434 results
1	(((((((((("Tendinopathy"[Mesh]) OR (Tendinopathies[Title/Abstract])) OR (Tendonopathy[Title/Abstract])) OR (Tendonopathies[Title/Abstract])) OR (Tendinitis[Title/Abstract])) OR (Tendinitides[Title/Abstract])) OR (Tendonitis[Title/Abstract])) OR (Tendonitides[Title/Abstract])) OR (Tendinosis[Title/Abstract])) OR (Tendinoses[Title/Abstract])) OR (Tendonosis[Title/Abstract])) OR (Tendonoses[Title/Abstract])	'tendinitis'/exp<br>'hypertrophic infiltrative tendinitis':ab,ti OR 'nodular tendinitis':ab,ti OR 'tendinopathy':ab,ti OR 'tendinosis':ab,ti OR 'tendonitis':ab,ti OR 'tendonopathy':ab,ti OR 'tenonitis':ab,ti OR 'tenontitis':ab,ti OR 'tenositis':ab,ti OR 'tendinitis':ab,ti	MeSH descriptor: [Tendinopathy] explode all trees<br>(Tendinoses):ti,ab,kw OR (Tendonoses):ti,ab,kw OR (Tendonosis):ti,ab,kw OR (Tendinosis):ti,ab,kw OR (Tendonopathies):ti,ab,kw OR (Tendonopathy):ti,ab,kw OR (Tendinopathies):ti,ab,kw OR (Tendonitis):ti,ab,kw OR (Tendinitis):ti,ab,kw OR (Tendonitides):ti,ab,kw OR (Tendinitides):ti,ab,kw	TS=(Tendinopathy OR Tendinoses OR Tendonoses OR Tendonosis OR Tendinosis OR Tendonopathies OR Tendonopathy OR Tendinopathies OR Tendonitis OR Tendinitis OR Tendonitides OR Tendinitides OR Electrotherapy)
2	((((((((((((((((("Rotator Cuff Injuries"[Mesh]) OR (Cuff Injury, Rotator[Title/Abstract])) OR (Injury, Rotator Cuff[Title/Abstract])) OR (Rotator Cuff Injury[Title/Abstract])) OR (Rotator Cuff Tears[Title/Abstract])) OR (Rotator Cuff Tear[Title/Abstract])) OR (Tear, Rotator Cuff[Title/Abstract])) OR (Tears, Rotator Cuff[Title/Abstract])) OR (Rotator Cuff Tendinitis[Title/Abstract])) OR (Rotator Cuff Tendinitides[Title/Abstract])) OR (Tendinitis, Rotator Cuff[Title/Abstract])) OR (Rotator Cuff Tendinosis[Title/Abstract])) OR (Rotator Cuff Tendinoses[Title/Abstract])) OR (Tendinoses, Rotator Cuff[Title/Abstract])) OR (Tendinosis, Rotator Cuff[Title/Abstract])) OR (Glenoid Labral Tears[Title/Abstract])) OR (Glenoid Labral Tear[Title/Abstract])) OR (Labral Tear, Glenoid[Title/Abstract])) OR (Labral Tears, Glenoid[Title/Abstract])) OR (Tear, Glenoid Labral[Title/Abstract])	'rotator cuff injury'/exp<br>'cuff injury':ab,ti OR 'cuff lesion':ab,ti OR 'rotator cuff disease':ab,ti OR 'rotator cuff disorder':ab,ti OR 'rotator cuff disorders':ab,ti OR 'rotator cuff injuries':ab,ti OR 'rotator cuff lesion':ab,ti OR 'rotator cuff lesions':ab,ti OR 'rotator cuff pathology':ab,ti OR 'rotator cuff tendinopathies':ab,ti OR 'rotator cuff tendinopathy':ab,ti OR 'shoulder rotator cuff degeneration':ab,ti OR 'shoulder rotator cuff lesion':ab,ti OR 'rotator cuff injury':ab,ti	MeSH descriptor: [Rotator Cuff Injuries] explode all trees<br>(Cuff Injury, Rotator):ti,ab,kw OR (Injury, Rotator Cuff):ti,ab,kw OR (Rotator Cuff Injury):ti,ab,kw OR (Rotator Cuff Tears):ti,ab,kw OR (Rotator Cuff Tear):ti,ab,kw OR (Tear, Rotator Cuff):ti,ab,kw OR (Tears, Rotator Cuff):ti,ab,kw OR<br>(Rotator Cuff Tendinitis):ti,ab,kw OR (Rotator Cuff Tendinitides):ti,ab,kw OR (Tendinitis, Rotator Cuff):ti,ab,kw OR (Rotator Cuff Tendinosis):ti,ab,kw OR (Rotator Cuff Tendinoses):ti,ab,kw OR (Tendinoses, Rotator Cuff):ti,ab,kw OR (Tendinosis, Rotator Cuff):ti,ab,kw OR (Glenoid Labral Tears):ti,ab,kw OR (Glenoid Labral Tear):ti,ab,kw OR (Labral Tear, Glenoid):ti,ab,kw OR (Labral Tears, Glenoid):ti,ab,kw OR (Tear, Glenoid Labral):ti,ab,kw	TS=(Rotator Cuff Injuries OR Cuff Injury, Rotator OR Injury, Rotator Cuff OR Rotator Cuff Injury OR Rotator Cuff Tears OR Rotator Cuff Tear OR Tear, Rotator Cuff OR Tears, Rotator Cuff OR Rotator Cuff Tendinitis OR Rotator Cuff Tendinitides OR Tendinitis, Rotator Cuff OR Rotator Cuff Tendinosis OR Rotator Cuff Tendinoses OR Tendinoses, Rotator Cuff OR Tendinosis, Rotator Cuff OR Glenoid Labral Tears OR Glenoid Labral Tear OR Labral Tear, Glenoid OR Labral Tears, Glenoid OR Tear, Glenoid Labral)<br>TS=(Elbow Tendinopathy OR Elbow Tendinopathies OR Tendinopathies, Elbow OR Tendinopathy, Elbow OR Medial Epicondylitis OR Epicondylitis, Medial OR Medial Epicondylitides OR Golfer's Elbow OR Golfers Elbow)
3	(((((((("Elbow Tendinopathy"[Mesh]) OR (Elbow Tendinopathies[Title/Abstract])) OR (Tendinopathies, Elbow[Title/Abstract])) OR (Tendinopathy, Elbow[Title/Abstract])) OR (Medial Epicondylitis[Title/Abstract])) OR (Epicondylitis, Medial[Title/Abstract])) OR (Medial Epicondylitides[Title/Abstract])) OR (Golfer's Elbow[Title/Abstract])) OR (Golfers Elbow[Title/Abstract])	'epicondylitis'/exp<br>'elbow tendinopathy':ab,ti OR 'elbow tendonopathy':ab,ti OR 'epicondylalgia':ab,ti OR 'epicondylitis':ab,ti	MeSH descriptor: [Elbow Tendinopathy] explode all trees<br>(Elbow Tendinopathies):ti,ab,kw OR (Tendinopathies, Elbow):ti,ab,kw OR (Tendinopathy, Elbow):ti,ab,kw OR (Medial Epicondylitis):ti,ab,kw OR (Epicondylitis, Medial):ti,ab,kw OR (Medial Epicondylitides):ti,ab,kw OR (Golfer's Elbow):ti,ab,kw OR (Golfers Elbow):ti,ab,kw	TS=(Tennis Elbow OR Elbow, Tennis OR Tennis Elbows OR Lateral Epicondylitis OR Epicondylitides, Lateral OR Epicondylitis, Lateral OR Lateral Epicondylitides OR Epicondylitis, Lateral Humeral OR Epicondylitides, Lateral Humeral OR Humeral Epicondylitides, Lateral OR Humeral Epicondylitis, Lateral OR Lateral Humeral Epicondylitides OR Lateral Humeral Epicondylitis)

4	((((((((((("Tennis Elbow"[Mesh]) OR (Elbow, Tennis[Title/Abstract])) OR (Tennis Elbows[Title/Abstract])) OR (Lateral Epicondylitis[Title/Abstract])) OR (Epicondylitides, Lateral[Title/Abstract])) OR (Epicondylitis, Lateral[Title/Abstract])) OR (Lateral Epicondylitides[Title/Abstract])) OR (Epicondylitis, Lateral Humeral[Title/Abstract])) OR (Epicondylitides, Lateral Humeral[Title/Abstract])) OR (Humeral Epicondylitides, Lateral[Title/Abstract])) OR (Humeral Epicondylitis, Lateral[Title/Abstract])) OR (Lateral Humeral Epicondylitides[Title/Abstract])) OR (Lateral Humeral Epicondylitis[Title/Abstract])	'tennis elbow'/exp<br>'bursitis, radiohumeral':ab,ti OR 'elbow, tennis':ab,ti OR 'epicondylatgia humeri':ab,ti OR 'epicondylitis humeri':ab,ti OR 'epicondylitis lateralis':ab,ti OR 'epicondylitis, external humeral':ab,ti OR 'epicondylitis, radiohumeral':ab,ti OR 'epycondylitis humeri':ab,ti OR 'external humeral epicondylitis':ab,ti OR 'humeral epicondylitis':ab,ti OR 'humerus epicondylitis':ab,ti OR 'lateral elbow tendinopathy':ab,ti OR 'lateral elbow tendinosis':ab,ti OR 'lateral elbow tendonopathy':ab,ti OR 'lateral epicondylitis':ab,ti OR 'lateral humeral epicondylitis':ab,ti OR 'radiohumeral bursitis':ab,ti OR 'radiohumeral epicondylitis':ab,ti OR 'tennis arm':ab,ti OR 'tennis elbow':ab,ti	MeSH descriptor: [Tennis Elbow] explode all trees<br>(Elbow, Tennis):ti,ab,kw OR<br>(Tennis Elbows):ti,ab,kw OR<br>(Lateral Epicondylitis):ti,ab,kw OR<br>(Epicondylitides, Lateral):ti,ab,kw OR<br>(Epicondylitis, Lateral):ti,ab,kw OR<br>(Lateral Epicondylitides):ti,ab,kw OR<br>(Epicondylitis, Lateral Humeral):ti,ab,kw OR<br>(Epicondylitides, Lateral Humeral):ti,ab,kw OR<br>(Humeral Epicondylitides, Lateral):ti,ab,kw OR<br>(Humeral Epicondylitis, Lateral):ti,ab,kw OR<br>(Lateral Humeral Epicondylitides):ti,ab,kw OR<br>(Lateral Humeral Epicondylitis):ti,ab,kw	TS=(patellar tendinopathy OR jumper’s knee OR patellar tendonitis OR patellar tendinitis OR patellar tendinosis)
5	((((patellar tendinopathy[Title/Abstract]) OR (jumper’s knee[Title/Abstract])) OR (patellar tendinosis[Title/Abstract])) OR (patellar tendinitis[Title/Abstract])) OR (patellar tendonitis[Title/Abstract])	'achilles tendinitis'/exp<br>'achilles tendinopathy':ab,ti OR 'achilles tendinosis':ab,ti OR 'achilles tendonopathy':ab,ti OR 'achilles tendinitis':ab,ti	(patellar tendinopathy):ti,ab,kw OR (jumper’s knee):ti,ab,kw OR (patellar tendonitis):ti,ab,kw OR (patellar tendinitis):ti,ab,kw OR (patellar tendinosis):ti,ab,kw	TS=(Achilles tendinopathy OR Insertional Achilles tendinopathy OR Mid-portion Achilles tendinopathy)
6	((Achilles tendinopathy[Title/Abstract]) OR (Mid-portion Achilles tendinopathy[Title/Abstract])) OR (Insertional Achilles tendinopathy[Title/Abstract])	'greater trochanter pain syndrome'/exp<br>'gtps':ab,ti OR 'trochanteric bursitis':ab,ti OR 'gluteal tendinopathy':ab,ti OR 'gluteus medius tendinopathy':ab,ti OR 'gluteus minimus tendinopathy':ab,ti OR 'greater trochanteric pain syndrome':ab,ti	(Achilles tendinopathy):ti,ab,kw OR (Insertional Achilles tendinopathy):ti,ab,kw OR (Mid-portion Achilles tendinopathy):ti,ab,kw	TS=(greater trochanter pain syndrome OR GTPS OR Trochanteric bursitis OR Gluteal tendinopathy OR Gluteus medius tendinopathy OR Gluteus minimus tendinopathy OR greater trochanteric pain syndrome)
7	((((((greater trochanter pain syndrome[Title/Abstract]) OR (GTPS[Title/Abstract])) OR (Trochanteric bursitis[Title/Abstract])) OR (Gluteal tendinopathy[Title/Abstract])) OR (Gluteus medius tendinopathy[Title/Abstract])) OR (Gluteus minimus tendinopathy[Title/Abstract])) OR (greater trochanteric pain syndrome[Title/Abstract])	'patellar tendinopathy':ab,ti OR 'jumpers knee':ab,ti OR 'patellar tendonitis':ab,ti OR 'patellar tendinitis':ab,ti OR 'patellar tendinosis':ab,ti	(greater trochanter pain syndrome):ti,ab,kw OR (GTPS):ti,ab,kw OR (Trochanteric bursitis):ti,ab,kw OR (Gluteal tendinopathy):ti,ab,kw OR (Gluteus medius tendinopathy):ti,ab,kw OR (Gluteus minimus tendinopathy):ti,ab,kw OR (greater trochanteric pain syndrome):ti,ab,kw	TS=(Extracorporeal Shockwave Therapy OR Extracorporeal Shockwave Therapies OR Shockwave Therapies, Extracorporeal OR Shockwave Therapy, Extracorporeal OR Therapy, Extracorporeal Shockwave OR Shock Wave Therapy OR Shock Wave Therapies OR Therapy, Shock Wave OR Extracorporeal Shock Wave Therapy OR Extracorporeal High-Intensity Focused Ultrasound Therapy OR Extracorporeal High Intensity Focused Ultrasound Therapy OR HIFU Therapy OR HIFU Therapies OR Therapy, HIFU OR High-Intensity Focused Ultrasound Therapy OR High Intensity Focused Ultrasound Therapy)

8	(((((((((((((("Extracorporeal Shockwave Therapy"[Mesh]) OR (Extracorporeal Shockwave Therapies[Title/Abstract])) OR (Shockwave Therapies, Extracorporeal[Title/Abstract])) OR (Shockwave Therapy, Extracorporeal[Title/Abstract])) OR (Therapy, Extracorporeal Shockwave[Title/Abstract])) OR (Extracorporeal Shock Wave Therapy[Title/Abstract])) OR (Shock Wave Therapy[Title/Abstract])) OR (Shock Wave Therapies[Title/Abstract])) OR (Therapy, Shock Wave[Title/Abstract])) OR (Extracorporeal High-Intensity Focused Ultrasound Therapy[Title/Abstract])) OR (Extracorporeal High Intensity Focused Ultrasound Therapy[Title/Abstract])) OR (High-Intensity Focused Ultrasound Therapy[Title/Abstract])) OR (High Intensity Focused Ultrasound Therapy[Title/Abstract])) OR (HIFU Therapy[Title/Abstract])) OR (HIFU Therapies[Title/Abstract])) OR (Therapy, HIFU[Title/Abstract])	'shock wave therapy'/exp<br>'extracorporeal shock wave therapy':ab,ti OR 'extracorporeal shockwave therapy':ab,ti OR 'shock wave treatment':ab,ti OR 'shockwave therapy':ab,ti OR 'shockwave treatment':ab,ti OR 'shock wave therapy':ab,ti	MeSH descriptor: [Extracorporeal Shockwave Therapy] explode all trees<br>(Extracorporeal Shockwave Therapies):ti,ab,kw OR (Shockwave Therapies, Extracorporeal):ti,ab,kw OR (Shockwave Therapy, Extracorporeal):ti,ab,kw OR (Therapy, Extracorporeal Shockwave):ti,ab,kw OR (Shock Wave Therapy):ti,ab,kw OR (Shock Wave Therapies):ti,ab,kw OR (Therapy, Shock Wave):ti,ab,kw OR (Extracorporeal Shock Wave Therapy):ti,ab,kw OR (Extracorporeal High-Intensity Focused Ultrasound Therapy):ti,ab,kw OR (Extracorporeal High Intensity Focused Ultrasound Therapy):ti,ab,kw OR (HIFU Therapy):ti,ab,kw OR (HIFU Therapies):ti,ab,kw OR (Therapy, HIFU):ti,ab,kw OR<br>(High-Intensity Focused Ultrasound Therapy):ti,ab,kw OR (High Intensity Focused Ultrasound Therapy):ti,ab,kw	TS=(Randomized Controlled Trial OR Clinical Trials, Randomized OR Trials, Randomized Clinical OR Controlled Clinical Trials, Randomized)
9	((("Randomized Controlled Trials as Topic"[Mesh]) OR (Clinical Trials, Randomized)) OR (Trials, Randomized Clinical)) OR (Controlled Clinical Trials, Randomized)	'randomized controlled trial' OR 'clinical trials, randomized' OR 'trials, randomized clinical' OR 'controlled clinical trials, randomized'	#1 OR #2 OR #3 OR #4 OR #5OR #6OR #7	#1 OR #2 OR #3 OR #4 OR #5 OR #6 OR #7
10	((("Randomized Controlled Trials as Topic"[Mesh]) OR (Clinical Trials, Randomized)) OR (Trials, Randomized Clinical)) OR (Controlled Clinical Trials, Randomized)	#1 OR #2 OR #3 OR #4 OR #5OR #6OR #7		
	**(#1 OR #2 OR #3 OR #4 OR #5OR #6OR #7)AND #8 AND #10**	**#8 AND #9 AND #10**	**#8 AND #9**	**#8 AND #9 AND #10**

Supplemental Table 4 List of included studies.

1	Sen S B, Kosehasanogullari M, Yilmaz N O, et al. Comparative analysis of the therapeutic effects of extracorporeal shock wave therapy and high-intensity laser therapy in lateral epicondylitis: a randomised clinical trial[J]. 2024,44(4):593-602.
2	Perveen W, Anwar S, Hashmi R, et al. Effects of extracorporeal shockwave therapy versus ultrasonic therapy and deep friction massage in the management of lateral epicondylitis: a randomized clinical trial[J]. Sci Rep,2024,14(1):16535.
3	Heaver C, Pinches M, Kuiper J H, et al. Greater trochanteric pain syndrome: focused shockwave therapy versus an ultrasound guided injection: a randomised control trial[J]. 2023,33(3):490-499.
4	Yagci H C, Yagci I, Bagcier F. Comparison of shock wave therapy and corticosteroid injection in the treatment of greater trochanteric pain syndrome: a single-blind, randomized study[J]. 2023,69(2):180-187.
5	Notarnicola A, Ladisa I, Lanzilotta P, et al. Shock Waves and Therapeutic Exercise in Greater Trochanteric Pain Syndrome: a Prospective Randomized Clinical Trial with Cross-Over[J]. 2023,13(6).
6	Stania M, Juras G, Marszalek W, et al. Analysis of pain intensity and postural control for assessing the efficacy of shock wave therapy and sonotherapy in Achilles tendinopathy - A randomized controlled trial[J]. 2023,101:105830.
7	Sah V, Delen V. The Efficacy of Large-Focused and Controlled-Unfocused (Radial) Extracorporeal Shock Wave Therapies in the treatment of Patellar Tendinopathy: a randomized sham-controlled single-blind trial[J]. 2023,3(1):38-44.
8	Kaplan S, Sah V, Ozkan S, et al. Comparative Effects of Focused and Radial Extracorporeal Shock Wave Therapies on Lateral Epicondylitis: a Randomised Sham-controlled Trial[J]. 2023,33(5):554-559.
9	Kim J, Lee H W, Cho Y T, et al. COMPARISON OF EXTRACORPOREAL SHOCK WAVE THERAPY AND ULTRASOUND GUIDED SHOULDER INJECTION THERAPY TO TREAT PATIENTS WITH SUPRASPINATUS TENDINITS[J]. 2023,7(5):1690.
10	Ebadi S, Karimzad Y, Aflakian N, et al. Extracorporeal shockwaves therapy versus corticosteroid injection for the treatment of non-calcific rotator cuff tendinopathies: a randomized trial[J]. 2023,34(4):185-190.
11	Elgendy M H, Mazen M M, Saied A M, et al. Extracorporeal Shock Wave Therapy vs. Corticosteroid Local Injection in Shoulder Impingement Syndrome : A Three-Arm Randomized Controlled Trial[J]. American journal of physical medicine & rehabilitation,2023,102(6):533-540.
12	Wheeler P C, Dudson C, Calver R, et al. Three Sessions of Radial Extracorporeal Shockwave Therapy Gives No Additional Benefit Over "Minimal-Dose" Radial Extracorporeal Shockwave Therapy for Patients With Chronic Greater Trochanteric Pain Syndrome: a Double-Blinded, Randomized, Controlled Trial[J]. 2022,32(1):e7-e18.
13	Benli M D, Tatari H, Balci A, et al. A comparison between the efficacy of eccentric exercise and extracorporeal shock wave therapy on tendon thickness, vascularity, and elasticity in Achilles tendinopathy: a randomized controlled trial[J]. 2022,68(3):372-380.
14	Fatima A, Ahmad A, Gilani S A, et al. Effects of High-Energy Extracorporeal Shockwave Therapy on Pain, Functional Disability, Quality of Life, and Ultrasonographic Changes in Patients with Calcified Rotator Cuff Tendinopathy[J]. 2022,2022:1230857.
15	Aldajah S, Alashram A R, Annino G, et al. Analgesic Effect of Extracorporeal Shock-Wave Therapy in Individuals with Lateral Epicondylitis: A Randomized Controlled Trial[J]. 2022,7(1).
16	Nambi G, Alghadier M, Ebrahim E E, et al. MRI and Ultrasound Analysis of Corticosteroid Injection Combined with Extracorporeal Shockwave Therapy in Lateral Epicondylitis—A Prospective, Randomized, Double-Blinded, Placebo-Controlled Trial[J]. 2022,12(11).
17	Kuo Y C, Hsu W C, Lin Y J, et al. Comparison of the effects of ultrasound- guided needle puncture, radial shock wave therapy, and combined treatments on calcific tendinitis of the shoulder: a single-blind randomized controlled trial[J]. 2022,35(5):1065-1074.
18	Versus Isolated Eccentric Exercises for Achilles Insertional Tendinopathy: a Double-Blinded Randomized Clinical Trial[J]. 2021,103(14):1295-1302.
19	Abdelkader N A, Helmy M, Fayaz N A, et al. Short- and Intermediate-Term Results of Extracorporeal Shockwave Therapy for Noninsertional Achilles Tendinopathy[J]. 2021,42(6):788-797.
20	özmen T, Koparal S S, Karataş Ö, et al. Comparison of the clinical and sonographic effects of ultrasound therapy, extracorporeal shock wave therapy, and Kinesio taping in lateral epicondylitis[J]. 2021,51(1):76-83.
21	Sahbaz T, Ceylan C M, Karacay B C, et al. Comparison of platelet-rich plasma and extracorporeal shock wave therapy in patients with chronic lateral epicondylitis: a prospective, randomized-controlled study[J]. 2021,67(4):490-501.
22	Shim B J, Seo E M, Hwang J T, et al. Comparison of the effectiveness of extensor muscle strengthening exercise by itself, exercise with polydeoxyribonucleotide injection, and exercise with extracorporeal shockwave therapy in lateral epicondylitis: a randomized controlled trial[J]. 2021,24(4):231-238.
23	Moretti L, Notarnicola A, Ostuni A, et al. Umbilical cord blood platelet-rich plasma injections for epicondylitis treatment: A prospective clinical study[J]. Journal of Biological Regulators and Homeostatic Agents,2021,35(4):1337-1342.
24	Ramon S, Russo S, Santoboni F, et al. Focused Shockwave Treatment for Greater Trochanteric Pain Syndrome: a Multicenter, Randomized, Controlled Clinical Trial[J]. 2020,102(15):1305-1311.
25	Pinitkwamdee S, Laohajaroensombat S, Orapin J, et al. Effectiveness of Extracorporeal Shockwave Therapy in the Treatment of Chronic Insertional Achilles Tendinopathy[J]. 2020,41(4):403-410.
26	Lee W C, Ng G Y, Zhang Z J, et al. Changes on Tendon Stiffness and Clinical Outcomes in Athletes Are Associated With Patellar Tendinopathy After Eccentric Exercise[J]. 2020,30(1):25-32.
27	Turgay T, Gunel Karadeniz P, Sever G B. Comparison of low level laser therapy and extracorporeal shock wave in treatment of chronic lateral epicondylitis[J]. 2020,54(6):591-595.

28	Guler T, Yildirim P. Comparison of the efficacy of kinesiotaping and extracorporeal shock wave therapy in patients with newly diagnosed lateral epicondylitis: a prospective randomized trial[J]. 2020,23(5):704-710.
29	Notarnicola A, Maccagnano G, Maresca L, et al. Is extracorporeal shockwave therapy effective even in the treatment of partial rotator cuff tear?[J]. 2020,34(2):709-714.
30	Atalay S G, Gezginaslan O. Effect of neural therapy versus extracorporeal shock wave therapy for the treatment of lateral epicondylitis: a randomized-controlled trial[J]. 2020,39.
31	Carlisi E, Cecini M, Di Natali G, et al. Focused extracorporeal shock wave therapy for greater trochanteric pain syndrome with gluteal tendinopathy: a randomized controlled trial[J]. 2019,33(4):670-680.
32	Cheng L, Chang S, Qian L, et al. Extracorporeal shock wave therapy for isokinetic muscle strength around the knee joint in athletes with patellar tendinopathy[J]. 2019,59(5):822-827.
33	Celik D, Anaforoglu Kulunkoglu B. Photobiomodulation Therapy Versus Extracorporeal Shock Wave Therapy in the Treatment of Lateral Epicondylitis[J]. 2019,37(5):269-275.
34	Ahadi T, Esmaeili Jamkarani M, Raissi G R, et al. Prolotherapy vs Radial Extracorporeal Shock Wave Therapy in the Short-term Treatment of Lateral Epicondylosis: a Randomized Clinical Trial[J]. 2019,20(9):1745-1749.
35	Duymaz T, Sindel D. Comparison of radial extracorporeal shock wave therapy and traditional physiotherapy in rotator cuff calcific tendinitis treatment[J]. 2019,34(3):281-287.
36	Atya A M, Nasser M M. Does Extracorporeal Shockwave Therapy Replace Joint Mobilization for the treatment of Subacromial Impingement[J]. BIOSCIENCE RESEARCH,2019,16(1):409-417.
37	Vahdatpour B, Forouzan H, Momeni F, et al. Effectiveness of extracorporeal shockwave therapy for chronic achilles tendinopathy: a randomized clinical trial[J]. 2018,23(4):37.
38	Yalvaç B, Mesci N, Geler Külcü D, et al. Comparison of ultrasound and extracorporeal shock wave therapy in lateral epicondylosis[J]. 2018,52(5):357-362.
39	Eraslan L, Yuce D, Erbilici A, et al. Does Kinesiotaping improve pain and functionality in patients with newly diagnosed lateral epicondylitis?[J]. 2018,26(3):938-945.
40	Aydin A, Atic R. Comparison of extracorporeal shock-wave therapy and wrist-extensor splint application in the treatment of lateral epicondylitis: a prospective randomized controlled study[J]. 2018,11:1459-1467.
41	Guler N S, Sargin S, Sahin N. Efficacy of extracorporeal shockwave therapy in patients with lateral epicondylitis: A randomized, placebo-controlled, double-blind clinical trial[J]. 2018,5(4):314-318.
42	Kvalvaag E, Roe C, Engebretsen K B, et al. One year results of a randomized controlled trial on radial Extracorporeal Shock Wave Treatment, with predictors of pain, disability and return to work in patients with subacromial pain syndrome[J]. European journal of physical and rehabilitation medicine,2018,54(3):341-350.
43	Thijs K M, Zwerver J, Backx F J, et al. Effectiveness of Shockwave Treatment Combined With Eccentric Training for Patellar Tendinopathy: a Double-Blinded Randomized Study[J]. 2017,27(2):89-96.
44	Yang T H, Huang Y C, Lau Y C, et al. Efficacy of Radial Extracorporeal Shock Wave Therapy on Lateral Epicondylosis, and Changes in the Common Extensor Tendon Stiffness with Pretherapy and Posttherapy in Real-Time Sonoelastography: a Randomized Controlled Study[J]. 2017,96(2):93-100.
45	Capan N, Esmaeilzadeh S, Oral A, et al. Radial Extracorporeal Shock Wave Therapy Is Not More Effective Than Placebo in the Management of Lateral Epicondylitis: a Double-Blind, Randomized, Placebo-Controlled Trial[J]. 2016,95(7):495-506.
46	Lizis P. Analgesic effect of extracorporeal shock wave therapy versus ultrasound therapy in chronic tennis elbow[J]. JOURNAL OF PHYSICAL THERAPY SCIENCE,2015,27(8):2563-2567.
47	Notarnicola A, Maccagnano G, Tafuri S, et al. CHELT therapy in the treatment of chronic insertional Achilles tendinopathy[J]. 2014,29(3):1217-1225.
48	Devrimsel G, Kucukali Turkyilmaz A, Yildirim M, et al. A comparison of laser and extracorporeal shock wave therapies in treatment of lateral epicondylitis[J]. 2014,60(3):194-198.
49	Efe T, Felgentreff M, Heyse T J, et al. Extracorporeal shock wave therapy for non-calcific supraspinatus tendinitis - 10-year follow-up of a randomized placebo-controlled trial[J]. 2014,59(5):431-437.
50	Vetrano M, Castorina A, Vulpiani M C, et al. Platelet-rich plasma versus focused shock waves in the treatment of jumper's knee in athletes[J]. 2013,41(4):795-803.
51	Sarkar B, Das P G, Equebal A, et al. Efficacy of low-energy extracorporeal shockwave therapy and a supervised clinical exercise protocol for the treatment of chronic lateral epicondylitis: a randomised controlled study[J]. 2013,31(1):19-24.
52	Zwerver J, Hartgens F, Verhagen E, et al. No effect of extracorporeal shockwave therapy on patellar tendinopathy in jumping athletes during the competitive season: a randomized clinical trial[J]. 2011,39(6):1191-1199.
53	Engebretsen K, Grotle M, Bautz-Holter E, et al. Supervised exercises compared with radial extracorporeal shock-wave therapy for subacromial shoulder pain: 1-year results of a single-blind randomized controlled trial.[J]. Physical therapy,2011,91(1):37-47.
54	Rompe J D, Segal N A, Cacchio A, et al. Home training, local corticosteroid injection, or radial shock wave therapy for greater trochanter pain syndrome[J]. 2009,37(10):1981-1990.
55	Rompe J D, Furia J, Maffulli N. Eccentric loading versus eccentric loading plus shock-wave treatment for midportion achilles tendinopathy: a randomized controlled trial[J]. 2009,37(3):463-470.

56	Rompe J D, Furia J, Maffulli N. Eccentric loading compared with shock wave treatment for chronic insertional achilles tendinopathy. A randomized, controlled trial[J]. 2008,90(1):52-61.
57	Staples M P, Forbes A, Ptasznik R, et al. A randomized controlled trial of extracorporeal shock wave therapy for lateral epicondylitis (tennis elbow)[J]. 2008,35(10):2038-2046.
58	Rompe J D, Nafe B, Furia J P, et al. Eccentric loading, shock-wave treatment, or a wait-and-see policy for tendinopathy of the main body of tendo Achillis: a randomized controlled trial[J]. 2007,35(3):374-383.
59	Wang C J, Ko J Y, Chan Y S, et al. Extracorporeal shockwave for chronic patellar tendinopathy[J]. 2007,35(6):972-978.
60	Costa M L, Shepstone L, Donell S T, et al. Shock wave therapy for chronic Achilles tendon pain: a randomized placebo-controlled trial[J]. 2005,440:199-204.
61	Pettrone F A, Mccall B R. Extracorporeal shock wave therapy without local anesthesia for chronic lateral epicondylitis[J]. 2005,87(6):1297-1304.
62	Rompe J D, Decking J, Schoellner C, et al. Repetitive low-energy shock wave treatment for chronic lateral epicondylitis in tennis players[J]. 2004,32(3):734-743.
63	K. Taunton J T K K. Treatment of patellar tendinopathy with extracorporeal shock wave therapy[J]. British Columbia Medical Journal,2003.
64	Speed C A, Nichols D, Richards C, et al. Extracorporeal shock wave therapy for lateral epicondylitis--a double blind randomised controlled trial[J]. 2002,20(5):895-898.
65	Speed C A, Richards C, Nichols D, et al. Extracorporeal shock-wave therapy for tendonitis of the rotator cuff[J]. Journal of Bone and Joint Surgery - Series B,2002,84(4):509-512.

Supplement Table 5. The GRADE evidence calssification of outcomes of tendinopathy in patients treated with ESWT.

	Research type	Number of studies	risk of bias	inconsistency	indirectness	imprecision	publication bias	Quality of evidence
Pain	RCT	57	NO	NO	NO	NO	NO	B
Function	RCT	33	YES	YES	NO	NO	NO	C

Supplemental Figure 1 Risk of bias for each included study.

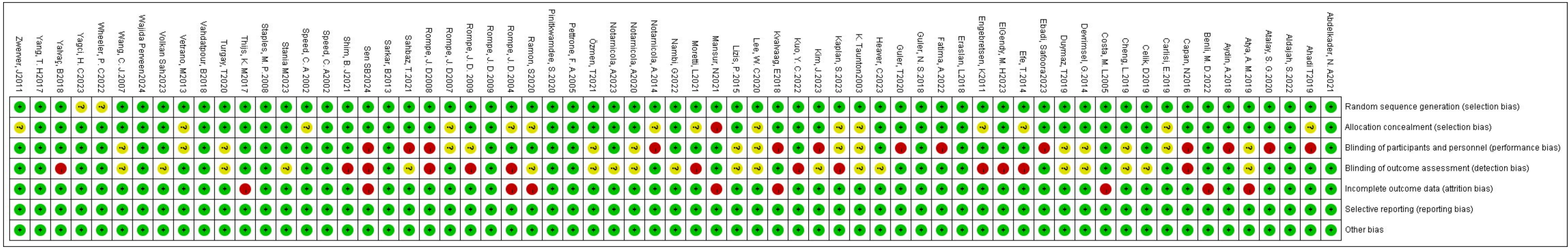

Supplemental Figure 2 Forest plot for pairwise meta-analysis of all 65 included studies.

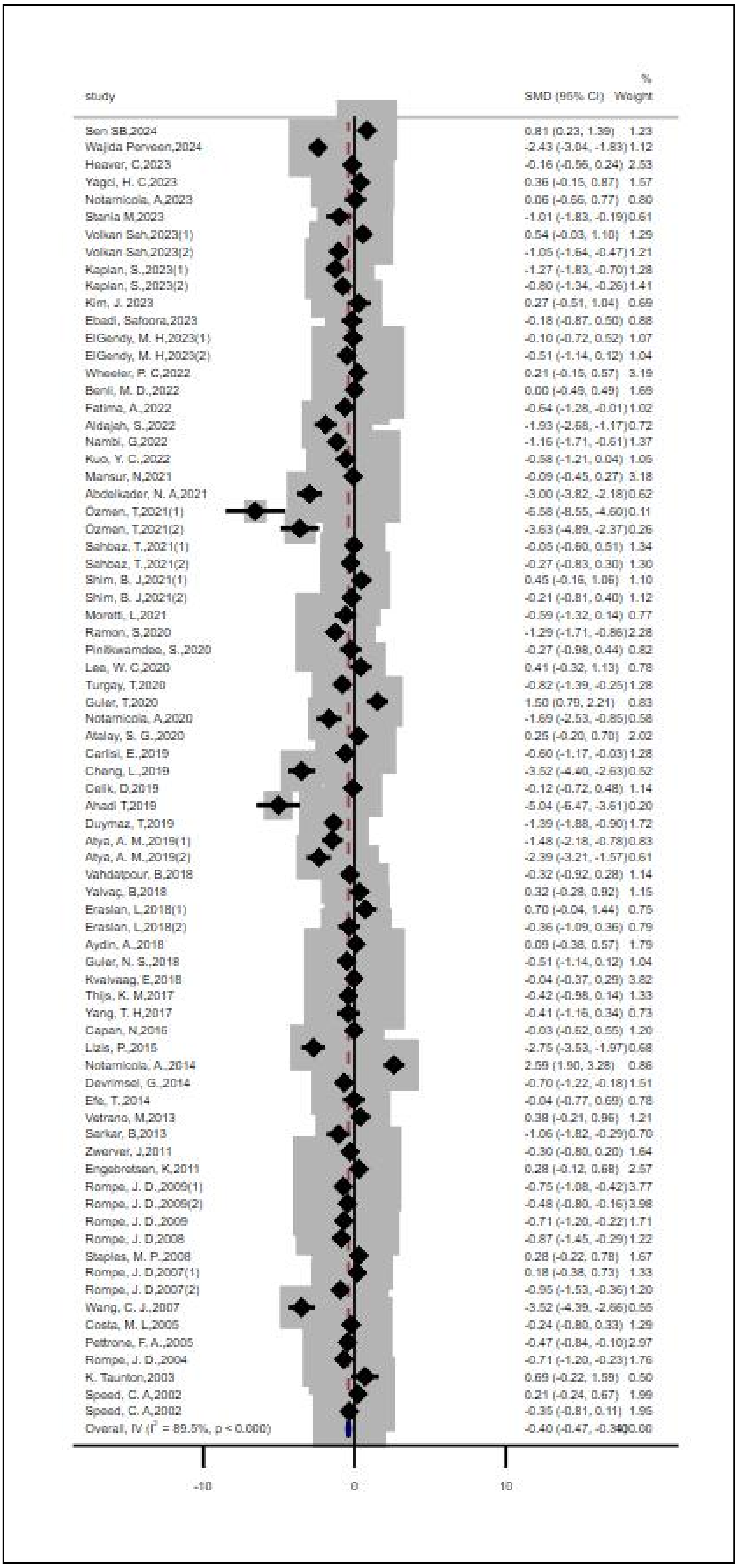

Supplemental Figure 3 Forest plot for pairwise meta-analysis of 45 studies with outcome measures on the 0-10 point VAS pain scale.

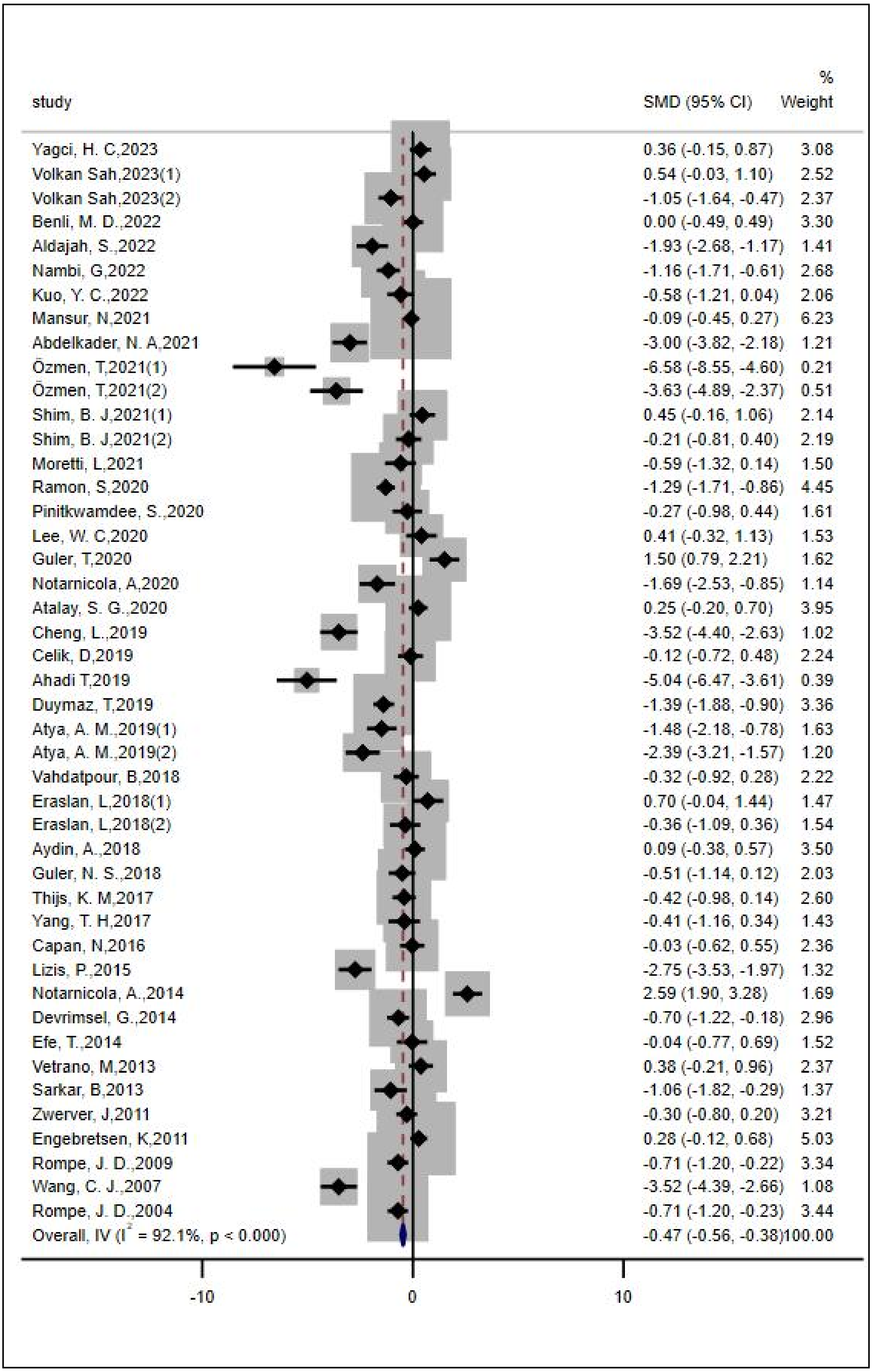

Supplemental Figure 4 A: Forest plot for pairwise meta-analysis of studies using the 0-10 point VAS scale, grouped by control intervention; B: Forest plot for pairwise meta-analysis of studies using the 0-10 point VAS scale, grouped by tendinopathy type (GTPS: greater trochanteric pain syndrome; PT: patellar tendinopathy; AT: Achilles tendinopathy; LE: lateral epicondylitis; RCT: rotator cuff tendinopathy).

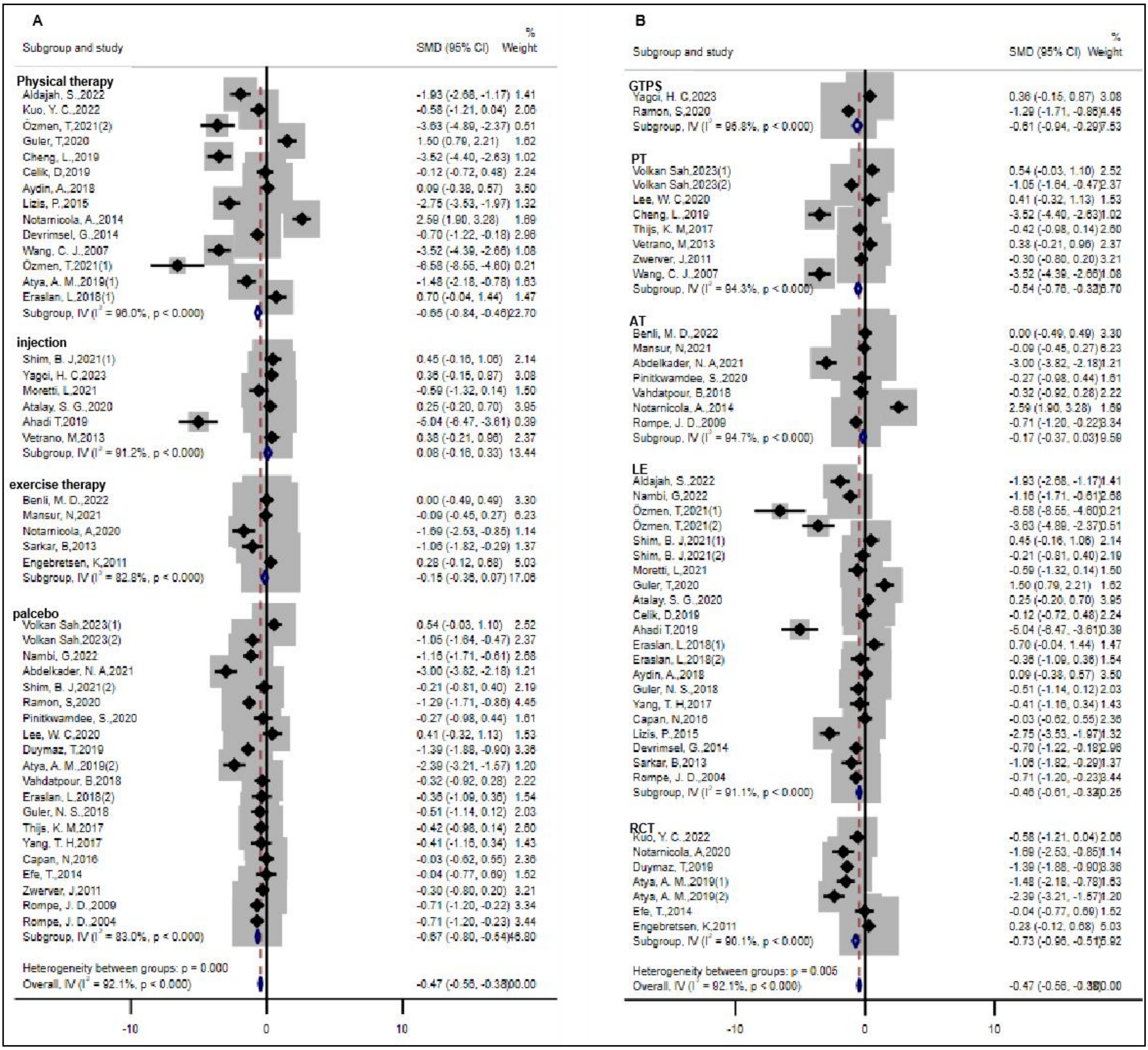

Supplemental Figure 5 Bias assessment for the pairwise meta-analysis of all 65 included studies: (A) Egger's test, (C) Funnel plot; Bias assessment for the pairwise meta-analysis of the 45 studies using the 0-10 point VAS pain scale: (B) Egger's test, (D) Funnel plot.

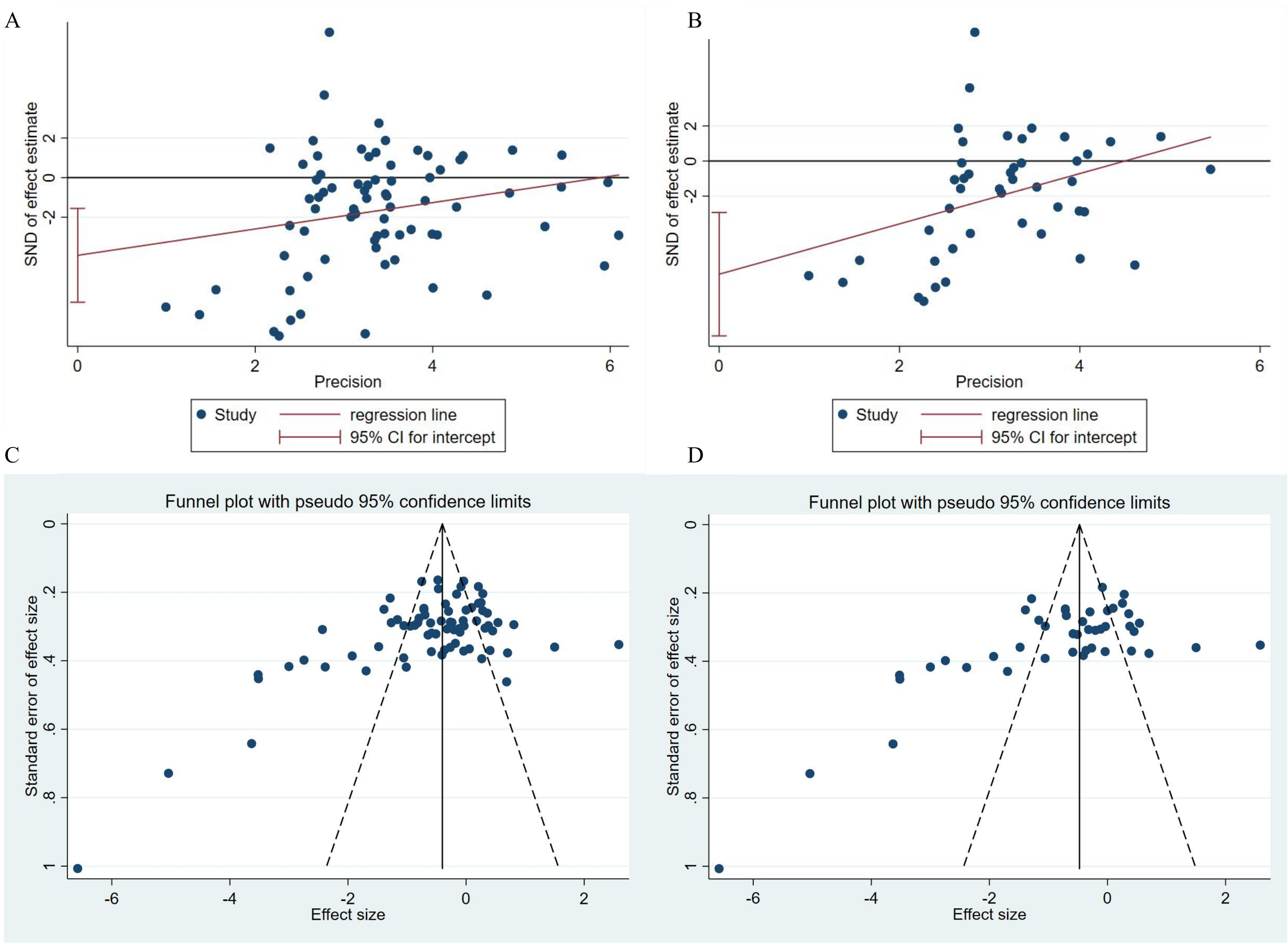

Supplemental Figure 6 Forest plots of eligible comparisons of tendinopathy.

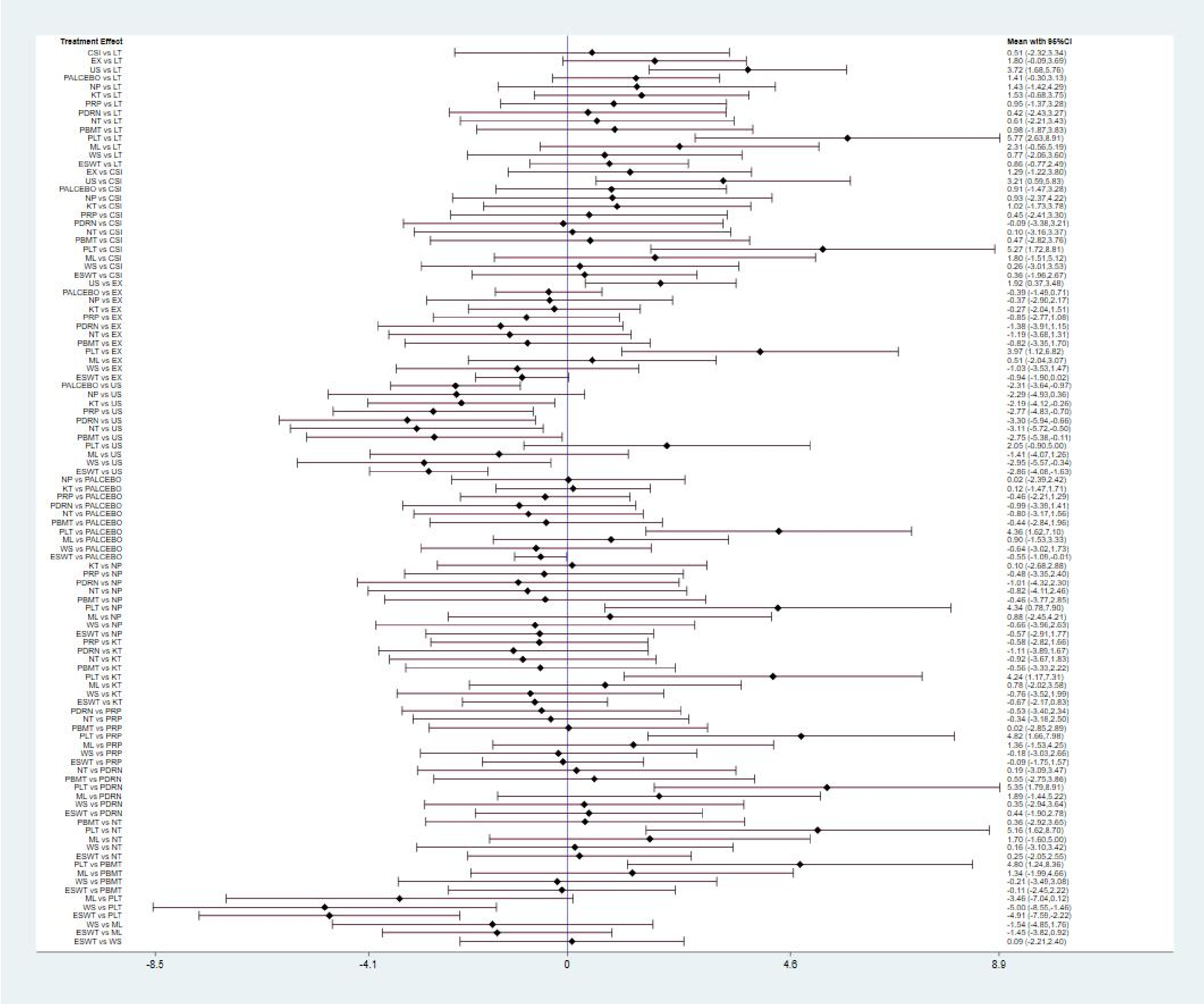

Supplemental Figure 6 -1 VAS in all types of tendinopathy

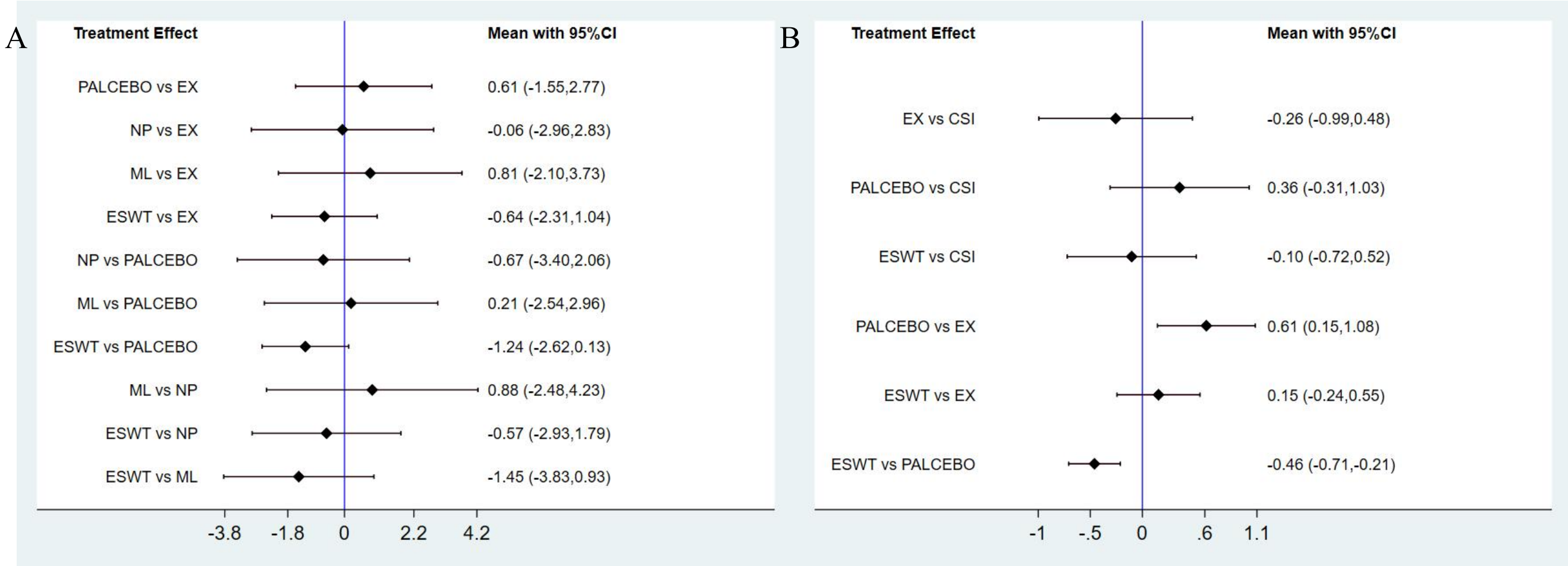

Supplemental Figure 6 -2 VAS(A) and SPADI(B) in rotator cuff tendinopathy

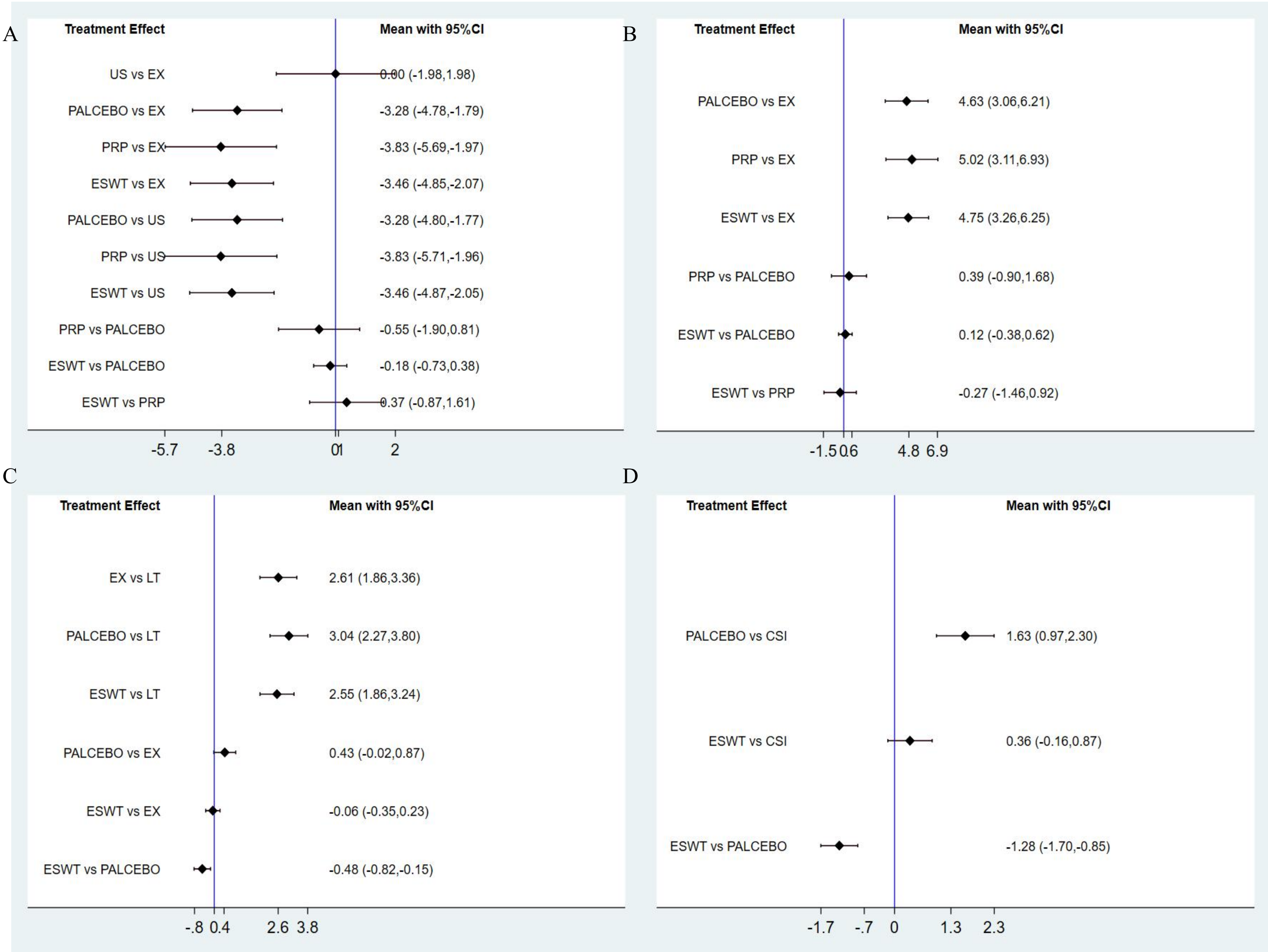

Supplemental Figure 6 -3 VAS (A) and PRTEE(B) in patellar tendinopathy; VAS in achilles tendinoapthy(C); NRS in GTPS(D)

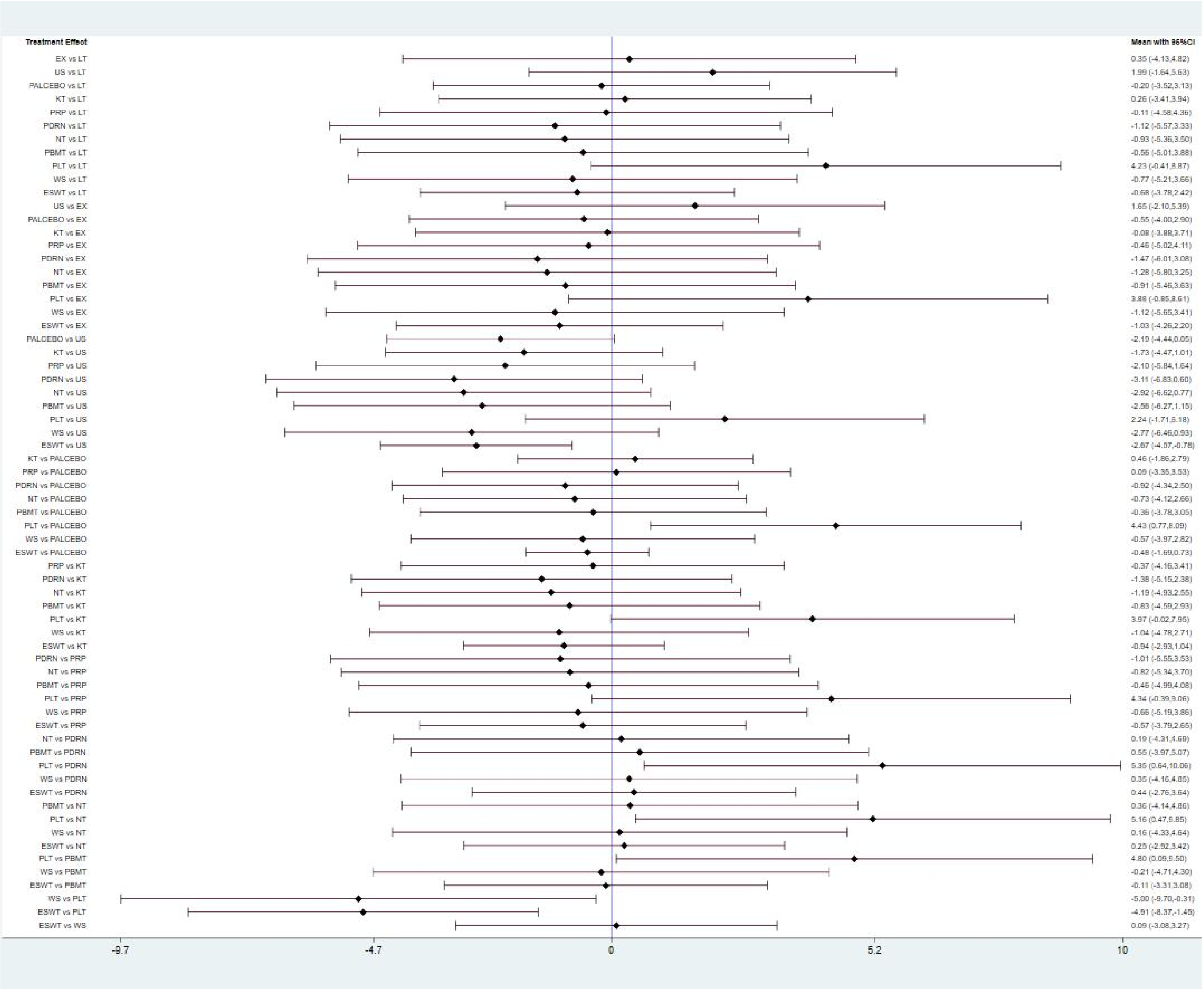

Supplemental Figure 6 -4 VAS in Lateral Epicondylitis

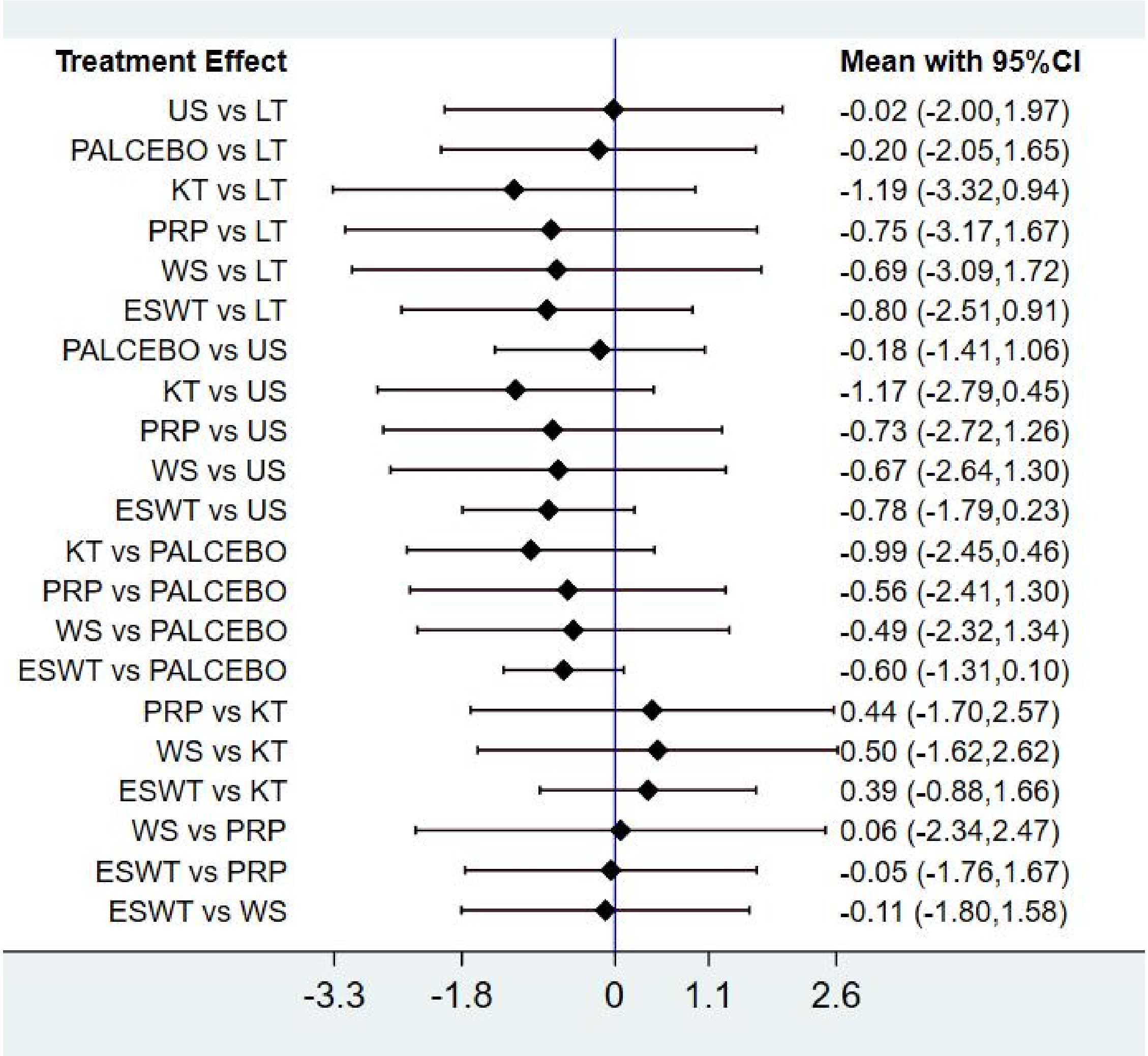

Supplemental Figure 6 -5 PRTEE in Lateral Epicondylitis

Supplemental Figure 7 League table of eligible comparisons of VAS in all kinds of tendinopathy(A), rotator cuff tendinopathy(B), Lateral Epicondylitis(D), achilles tendinopathy(F), patella tendinopathy(G), GTPS(I); SPADI in rotator cuff tendinopathy(C); PRTEE in Lateral Epicondylitis(E); VISA-P in patellar tendinopathy(H)

A

LT	0.51 (-2.32,3.34)	1.80 (-0.09,3.69)	3.72 (1.68,5.76)	1.41 (-0.30,3.13)	1.43 (-1.42,4.29)	1.53 (-0.68,3.75)	0.95 (-1.37,3.28)	0.42 (-2.43,3.27)	0.61 (-2.21,3.43)	0.98 (-1.87,3.83)	5.77 (2.63,8.91)	2.31 (-0.56,5.19)	0.77 (-2.06,3.60)	0.86 (-0.77,2.49)
-0.51 (-3.34,2.32)	CSI	1.29 (-1.22,3.80)	3.21 (0.59,5.83)	0.91 (-1.47,3.28)	0.93 (-2.37,4.22)	1.02 (-1.73,3.78)	0.45 (-2.41,3.30)	-0.09 (-3.38,3.21)	0.10 (-3.16,3.37)	0.47 (-2.82,3.76)	5.27 (1.72,8.81)	1.80 (-1.51,5.12)	0.26 (-3.01,3.53)	0.36 (-1.96,2.67)
-1.80 (-3.69,0.09)	-1.29 (-3.80,1.22)	EX	1.92 (0.37,3.48)	-0.39 (-1.49,0.71)	-0.37 (-2.90,2.17)	-0.27 (-2.04,1.51)	-0.85 (-2.77,1.08)	-1.38 (-3.91,1.15)	-1.19 (-3.68,1.31)	-0.82 (-3.35,1.70)	3.97 (1.12,6.82)	0.51 (-2.04,3.07)	-1.03 (-3.53,1.47)	-0.94 (-1.90,0.02)
-3.72 (-5.76,-1.68)	-3.21 (-5.83,-0.59)	-1.92 (-3.48,-0.37)	US	-2.31 (-3.64,-0.97)	-2.29 (-4.93,0.36)	-2.19 (-4.12,-0.26)	-2.77 (-4.83,-0.70)	-3.30 (-5.94,-0.66)	-3.11 (-5.72,-0.50)	-2.75 (-5.38,-0.11)	2.05 (-0.90,5.00)	-1.41 (-4.07,1.26)	-2.95 (-5.57,-0.34)	-2.86 (-4.08,-1.63)
-1.41 (-3.13,0.30)	-0.91 (-3.28,1.47)	0.39 (-0.71,1.49)	2.31 (0.97,3.64)	PALCEBO	0.02 (-2.39,2.42)	0.12 (-1.47,1.71)	-0.46 (-2.21,1.29)	-0.99 (-3.39,1.41)	-0.80 (-3.17,1.56)	-0.44 (-2.84,1.96)	4.36 (1.62,7.10)	0.90 (-1.53,3.33)	-0.64 (-3.02,1.73)	-0.55 (-1.09,-0.01)
-1.43 (-4.29,1.42)	-0.93 (-4.22,2.37)	0.37 (-2.17,2.90)	2.29 (-0.36,4.93)	-0.02 (-2.42,2.39)	NP	0.10 (-2.68,2.88)	-0.48 (-3.35,2.40)	-1.01 (-4.32,2.30)	-0.82 (-4.11,2.46)	-0.46 (-3.77,2.85)	4.34 (0.78,7.90)	0.88 (-2.45,4.21)	-0.66 (-3.96,2.63)	-0.57 (-2.91,1.77)
-1.53 (-3.75,0.68)	-1.02 (-3.78,1.73)	0.27 (-1.51,2.04)	2.19 (0.26,4.12)	-0.12 (-1.71,1.47)	-0.10 (-2.88,2.68)	KT	-0.58 (-2.82,1.66)	-1.11 (-3.89,1.67)	-0.92 (-3.67,1.83)	-0.56 (-3.33,2.22)	4.24 (1.17,7.31)	0.78 (-2.02,3.58)	-0.76 (-3.52,1.99)	-0.67 (-2.17,0.83)
-0.95 (-3.28,1.37)	-0.45 (-3.30,2.41)	0.85 (-1.08,2.77)	2.77 (0.70,4.83)	0.46 (-1.29,2.21)	0.48 (-2.40,3.35)	0.58 (-1.66,2.82)	PRP	-0.53 (-3.40,2.34)	-0.34 (-3.18,2.50)	0.02 (-2.85,2.89)	4.82 (1.66,7.98)	1.36 (-1.53,4.25)	-0.18 (-3.03,2.66)	-0.09 (-1.75,1.57)
-0.42 (-3.27,2.43)	0.09 (-3.21,3.38)	1.38 (-1.15,3.91)	3.30 (0.66,5.94)	0.99 (-1.41,3.39)	1.01 (-2.30,4.32)	1.11 (-1.67,3.89)	0.53 (-2.34,3.40)	PDRN	0.19 (-3.09,3.47)	0.55 (-2.75,3.86)	5.35 (1.79,8.91)	1.89 (-1.44,5.22)	0.35 (-2.94,3.64)	0.44 (-1.90,2.78)
-0.61 (-3.43,2.21)	-0.10 (-3.37,3.16)	1.19 (-1.31,3.68)	3.11 (0.50,5.72)	0.80 (-1.56,3.17)	0.82 (-2.46,4.11)	0.92 (-1.83,3.67)	0.34 (-2.50,3.18)	-0.19 (-3.47,3.09)	NT	0.36 (-2.92,3.65)	5.16 (1.62,8.70)	1.70 (-1.60,5.00)	0.16 (-3.10,3.42)	0.25 (-2.05,2.55)
-0.98 (-3.83,1.87)	-0.47 (-3.76,2.82)	0.82 (-1.70,3.35)	2.75 (0.11,5.38)	0.44 (-1.96,2.84)	0.46 (-2.85,3.77)	0.56 (-2.22,3.33)	-0.02 (-2.89,2.85)	-0.55 (-3.86,2.75)	-0.36 (-3.65,2.92)	PBMT	4.80 (1.24,8.36)	1.34 (-1.99,4.66)	-0.21 (-3.49,3.08)	-0.11 (-2.45,2.22)
-5.77 (-8.91,-2.63)	-5.27 (-8.81,-1.72)	-3.97 (-6.82,-1.12)	-2.05 (-5.00,0.90)	-4.36 (-7.10,-1.62)	-4.34 (-7.90,-0.78)	-4.24 (-7.31,-1.17)	-4.82 (-7.98,-1.66)	-5.35 (-8.91,-1.79)	-5.16 (-8.70,-1.62)	-4.80 (-8.36,-1.24)	PLT	-3.46 (-7.04,0.12)	-5.00 (-8.55,-1.46)	-4.91 (-7.59,-2.22)
-2.31 (-5.19,0.56)	-1.80 (-5.12,1.51)	-0.51 (-3.07,2.04)	1.41 (-1.26,4.07)	-0.90 (-3.33,1.53)	-0.88 (-4.21,2.45)	-0.78 (-3.58,2.02)	-1.36 (-4.25,1.53)	-1.89 (-5.22,1.44)	-1.70 (-5.00,1.60)	-1.34 (-4.66,1.99)	3.46 (-0.12,7.04)	ML	-1.54 (-4.85,1.76)	-1.45 (-3.82,0.92)
-0.77 (-3.60,2.06)	-0.26 (-3.53,3.01)	1.03 (-1.47,3.53)	2.95 (0.34,5.57)	0.64 (-1.73,3.02)	0.66 (-2.63,3.96)	0.76 (-1.99,3.52)	0.18 (-2.66,3.03)	-0.35 (-3.64,2.94)	-0.16 (-3.42,3.10)	0.21 (-3.08,3.49)	5.00 (1.46,8.55)	1.54 (-1.76,4.85)	WS	0.09 (-2.21,2.40)
-0.86 (-2.49,0.77)	-0.36 (-2.67,1.96)	0.94 (-0.02,1.90)	2.86 (1.63,4.08)	0.55 (0.01,1.09)	0.57 (-1.77,2.91)	0.67 (-0.83,2.17)	0.09 (-1.57,1.75)	-0.44 (-2.78,1.90)	-0.25 (-2.55,2.05)	0.11 (-2.22,2.45)	4.91 (2.22,7.59)	1.45 (-0.92,3.82)	-0.09 (-2.40,2.21)	ESWT

B

EX	0.61 (-1.55,2.77)	-0.06 (-2.96,2.83)	0.81 (-2.10,3.73)	-0.64 (-2.31,1.04)
-0.61 (-2.77,1.55)	PALCEBO	-0.67 (-3.40,2.06)	0.21 (-2.54,2.96)	-1.24 (-2.62,0.13)
0.06 (-2.83,2.96)	0.67 (-2.06,3.40)	NP	0.88 (-2.48,4.23)	-0.57 (-2.93,1.79)
-0.81 (-3.73,2.10)	-0.21 (-2.96,2.54)	-0.88 (-4.23,2.48)	ML	-1.45 (-3.83,0.93)
0.64 (-1.04,2.31)	1.24 (-0.13,2.62)	0.57 (-1.79,2.93)	1.45 (-0.93,3.83)	ESWT

C

CSI	-0.26 (-0.99,0.48)	0.36 (-0.31,1.03)	-0.10 (-0.72,0.52)
0.26 (-0.48,0.99)	EX	0.61 (0.15,1.08)	0.15 (-0.24,0.55)
-0.36 (-1.03,0.31)	-0.61 (-1.08,-0.15)	PALCEBO	-0.46 (-0.71,-0.21)
0.10 (-0.52,0.72)	-0.15 (-0.55,0.24)	0.46 (0.21,0.71)	ESWT

D

LT	0.35 (-4.13,4.82)	1.99 (-1.64,5.63)	-0.20 (-3.52,3.13)	0.26 (-3.41,3.94)	-0.11 (-4.58,4.36)	-1.12 (-5.57,3.33)	-0.93 (-5.36,3.50)	-0.56 (-5.01,3.88)	4.23 (-0.41,8.87)	-0.77 (-5.21,3.66)	-0.68 (-3.78,2.42)
-0.35 (-4.82,4.13)	EX	1.65 (-2.10,5.39)	-0.55 (-4.00,2.90)	-0.08 (-3.88,3.71)	-0.46 (-5.02,4.11)	-1.47 (-6.01,3.08)	-1.28 (-5.80,3.25)	-0.91 (-5.46,3.63)	3.88 (-0.85,8.61)	-1.12 (-5.65,3.41)	-1.03 (-4.26,2.20)
-1.99 (-5.63,1.64)	-1.65 (-5.39,2.10)	US	-2.19 (-4.44,0.05)	-1.73 (-4.47,1.01)	-2.10 (-5.84,1.64)	-3.11 (-6.83,0.60)	-2.92 (-6.62,0.77)	-2.56 (-6.27,1.15)	2.24 (-1.71,6.18)	-2.77 (-6.46,0.93)	-2.67 (-4.57,-0.78)
0.20 (-3.13,3.52)	0.55 (-2.90,4.00)	2.19 (-0.05,4.44)	PALCEBO	0.46 (-1.86,2.79)	0.09 (-3.35,3.53)	-0.92 (-4.34,2.50)	-0.73 (-4.12,2.66)	-0.36 (-3.78,3.05)	4.43 (0.77,8.09)	-0.57 (-3.97,2.82)	-0.48 (-1.69,0.73)
-0.26 (-3.94,3.41)	0.08 (-3.71,3.88)	1.73 (-1.01,4.47)	-0.46 (-2.79,1.86)	KT	-0.37 (-4.16,3.41)	-1.38 (-5.15,2.38)	-1.19 (-4.93,2.55)	-0.83 (-4.59,2.93)	3.97 (-0.02,7.95)	-1.04 (-4.78,2.71)	-0.94 (-2.93,1.04)
0.11 (-4.36,4.58)	0.46 (-4.11,5.02)	2.10 (-1.64,5.84)	-0.09 (-3.53,3.35)	0.37 (-3.41,4.16)	PRP	-1.01 (-5.55,3.53)	-0.82 (-5.34,3.70)	-0.46 (-4.99,4.08)	4.34 (-0.39,9.06)	-0.66 (-5.19,3.86)	-0.57 (-3.79,2.65)
1.12 (-3.33,5.57)	1.47 (-3.08,6.01)	3.11 (-0.60,6.83)	0.92 (-2.50,4.34)	1.38 (-2.38,5.15)	1.01 (-3.53,5.55)	PDRN	0.19 (-4.31,4.69)	0.55 (-3.97,5.07)	5.35 (0.64,10.06)	0.35 (-4.16,4.85)	0.44 (-2.76,3.64)
0.93 (-3.50,5.36)	1.28 (-3.25,5.80)	2.92 (-0.77,6.62)	0.73 (-2.66,4.12)	1.19 (-2.55,4.93)	0.82 (-3.70,5.34)	-0.19 (-4.69,4.31)	NT	0.36 (-4.14,4.86)	5.16 (0.47,9.85)	0.16 (-4.33,4.64)	0.25 (-2.92,3.42)
0.56 (-3.88,5.01)	0.91 (-3.63,5.46)	2.56 (-1.15,6.27)	0.36 (-3.05,3.78)	0.83 (-2.93,4.59)	0.46 (-4.08,4.99)	-0.55 (-5.07,3.97)	-0.36 (-4.86,4.14)	PBMT	4.80 (0.09,9.50)	-0.21 (-4.71,4.30)	-0.11 (-3.31,3.08)
-4.23 (-8.87,0.41)	-3.88 (-8.61,0.85)	-2.24 (-6.18,1.71)	-4.43 (-8.09,-0.77)	-3.97 (-7.95,0.02)	-4.34 (-9.06,0.39)	-5.35 (-10.06,-0.64)	-5.16 (-9.85,-0.47)	-4.80 (-9.50,-0.09)	PLT	-5.00 (-9.70,-0.31)	-4.91 (-8.37,-1.45)
0.77 (-3.66,5.21)	1.12 (-3.41,5.65)	2.77 (-0.93,6.46)	0.57 (-2.82,3.97)	1.04 (-2.71,4.78)	0.66 (-3.86,5.19)	-0.35 (-4.85,4.16)	-0.16 (-4.64,4.33)	0.21 (-4.30,4.71)	5.00 (0.31,9.70)	WS	0.09 (-3.08,3.27)
0.68 (-2.42,3.78)	1.03 (-2.20,4.26)	2.67 (0.78,4.57)	0.48 (-0.73,1.69)	0.94 (-1.04,2.93)	0.57 (-2.65,3.79)	-0.44 (-3.64,2.76)	-0.25 (-3.42,2.92)	0.11 (-3.08,3.31)	4.91 (1.45,8.37)	-0.09 (-3.27,3.08)	ESWT

E

LT	-0.02 (-2.00,1.97)	-0.20 (-2.05,1.65)	-1.19 (-3.32,0.94)	-0.75 (-3.17,1.67)	-0.69 (-3.09,1.72)	-0.80 (-2.51,0.91)
0.02 (-1.97,2.00)	US	-0.18 (-1.41,1.06)	-1.17 (-2.79,0.45)	-0.73 (-2.72,1.26)	-0.67 (-2.64,1.30)	-0.78 (-1.79,0.23)
0.20 (-1.65,2.05)	0.18 (-1.06,1.41)	PALCEBO	-0.99 (-2.45,0.46)	-0.56 (-2.41,1.30)	-0.49 (-2.32,1.34)	-0.60 (-1.31,0.10)
1.19 (-0.94,3.32)	1.17 (-0.45,2.79)	0.99 (-0.46,2.45)	KT	0.44 (-1.70,2.57)	0.50 (-1.62,2.62)	0.39 (-0.88,1.66)
0.75 (-1.67,3.17)	0.73 (-1.26,2.72)	0.56 (-1.30,2.41)	-0.44 (-2.57,1.70)	PRP	0.06 (-2.34,2.47)	-0.05 (-1.76,1.67)
0.69 (-1.72,3.09)	0.67 (-1.30,2.64)	0.49 (-1.34,2.32)	-0.50 (-2.62,1.62)	-0.06 (-2.47,2.34)	WS	-0.11 (-1.80,1.58)
0.80 (-0.91,2.51)	0.78 (-0.23,1.79)	0.60 (-0.10,1.31)	-0.39 (-1.66,0.88)	0.05 (-1.67,1.76)	0.11 (-1.58,1.80)	ESWT

F

LT	2.61 (1.86,3.36)	3.04 (2.27,3.80)	2.55 (1.86,3.24)
-2.61 (-3.36,-1.86)	EX	0.43 (-0.02,0.87)	-0.06 (-0.35,0.23)
-3.04 (-3.80,-2.27)	-0.43 (-0.87,0.02)	PALCEBO	-0.48 (-0.82,-0.15)
-2.55 (-3.24,-1.86)	0.06 (-0.23,0.35)	0.48 (0.15,0.82)	ESWT

G

EX	0.00 (-1.98,1.98)	-3.28 (-4.78,-1.79)	-3.83 (-5.69,-1.97)	-3.46 (-4.85,-2.07)
-0.00 (-1.98,1.98)	US	-3.28 (-4.80,-1.77)	-3.83 (-5.71,-1.96)	-3.46 (-4.87,-2.05)
3.28 (1.79,4.78)	3.28 (1.77,4.80)	PALCEBO	-0.55 (-1.90,0.81)	-0.18 (-0.73,0.38)
3.83 (1.97,5.69)	3.83 (1.96,5.71)	0.55 (-0.81,1.90)	PRP	0.37 (-0.87,1.61)
3.46 (2.07,4.85)	3.46 (2.05,4.87)	0.18 (-0.38,0.73)	-0.37 (-1.61,0.87)	ESWT

H

EX	4.63 (3.06,6.21)	5.02 (3.11,6.93)	4.75 (3.26,6.25)
-4.63 (-6.21,-3.06)	PALCEBO	0.39 (-0.90,1.68)	0.12 (-0.38,0.62)
-5.02 (-6.93,-3.11)	-0.39 (-1.68,0.90)	PRP	-0.27 (-1.46,0.92)
-4.75 (-6.25,-3.26)	-0.12 (-0.62,0.38)	0.27 (-0.92,1.46)	ESWT

I

CSI	1.63 (0.97,2.30)	0.36 (-0.16,0.87)
-1.63 (-2.30,-0.97)	PALCEBO	-1.28 (-1.70,-0.85)
-0.36 (-0.87,0.16)	1.28 (0.85,1.70)	ESWT

Supplemental Figure 8 Funnel plot of network meta analysis for VAS in all kinds of tendinopathy(A), rotator cuff tendinopathy(B), Lateral Epicondylitis(D), achilles tendinopathy(F), patella tendinopathy(G), GTPS(I); SPADI in rotator cuff tendinopathy(C); PRTEE in Lateral Epicondylitis(E); VISA-P in patellar tendinopathy(H)

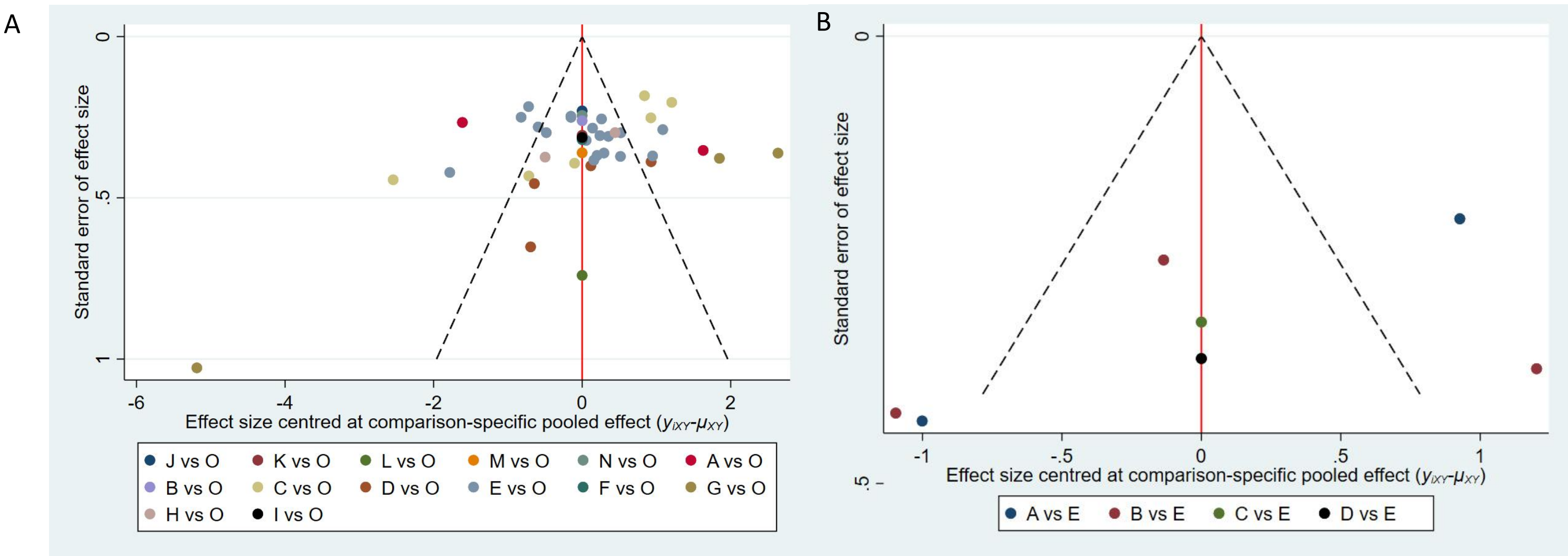

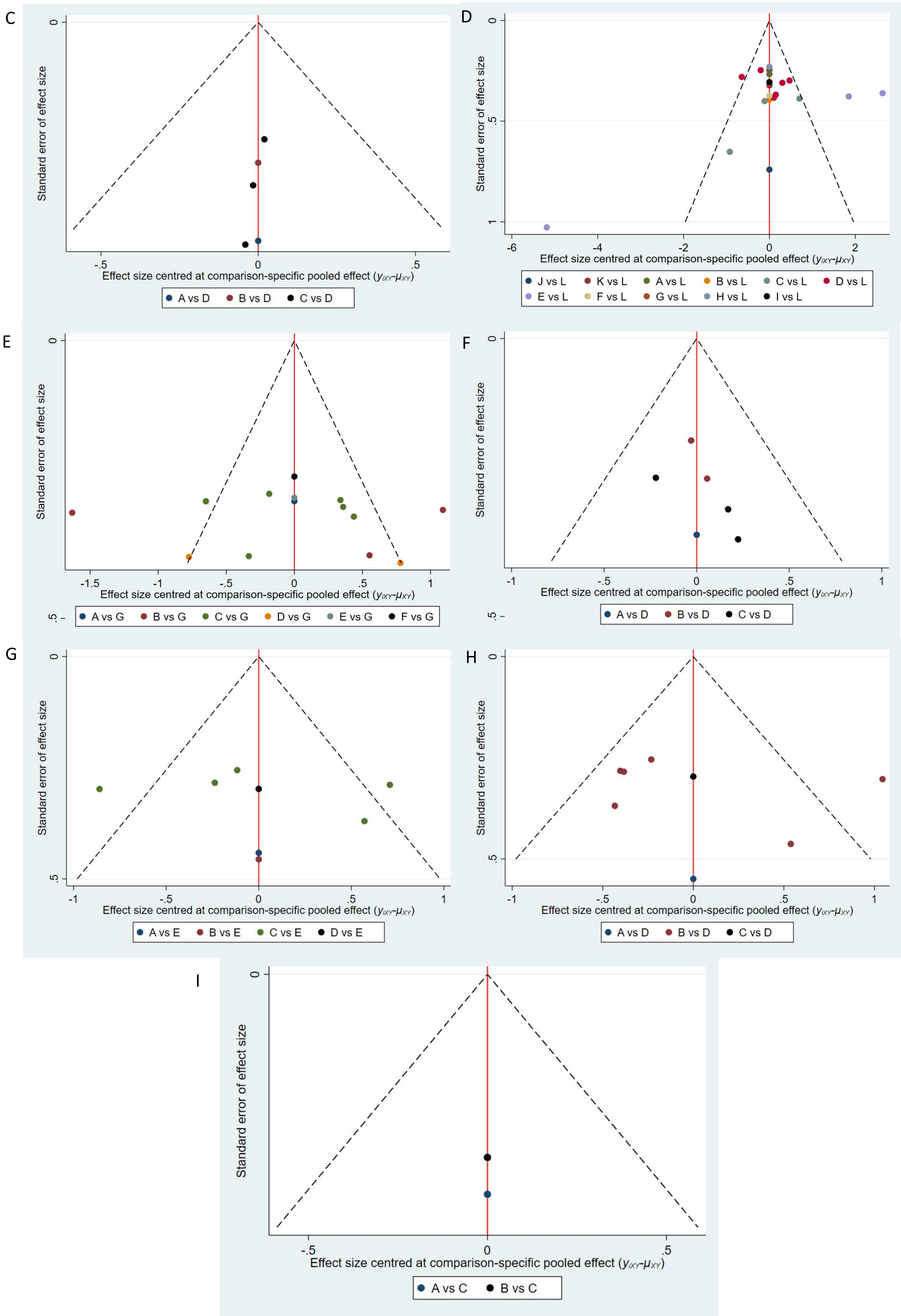

C
Standard error of effect size
Effect size centred at comparison-specific pooled effect ($y_{iXY}-\mu_{XY}$)
A vs D B vs D C vs D
D
Standard error of effect size
Effect size centred at comparison-specific pooled effect ($y_{iXY}-\mu_{XY}$)
J vs L K vs L A vs L B vs L C vs L D vs L
E vs L F vs L G vs L H vs L I vs L
E
Standard error of effect size
Effect size centred at comparison-specific pooled effect ($y_{iXY}-\mu_{XY}$)
A vs G B vs G C vs G D vs G E vs G F vs G
F
Standard error of effect size
Effect size centred at comparison-specific pooled effect ($y_{iXY}-\mu_{XY}$)
A vs D B vs D C vs D
G
Standard error of effect size
Effect size centred at comparison-specific pooled effect ($y_{iXY}-\mu_{XY}$)
A vs E B vs E C vs E D vs E
H
Standard error of effect size
Effect size centred at comparison-specific pooled effect ($y_{iXY}-\mu_{XY}$)
A vs D B vs D C vs D
I
Standard error of effect size
Effect size centred at comparison-specific pooled effect ($y_{iXY}-\mu_{XY}$)
A vs C B vs C

Supplemental Figure 9 Contributions of direct and indirect comparisons to NMA and the number of studies of each direct comparison of VAS in all kinds of tendinopathy(A), rotator cuff tendinopathy(B), Lateral Epicondylitis(D), achilles tendinopathy(F), patella tendinopathy(G), GTPS(I); SPADI in rotator cuff tendinopathy(C); PRTEE in Lateral Epicondylitis(E); VISA-P in patellar tendinopathy(H)

A

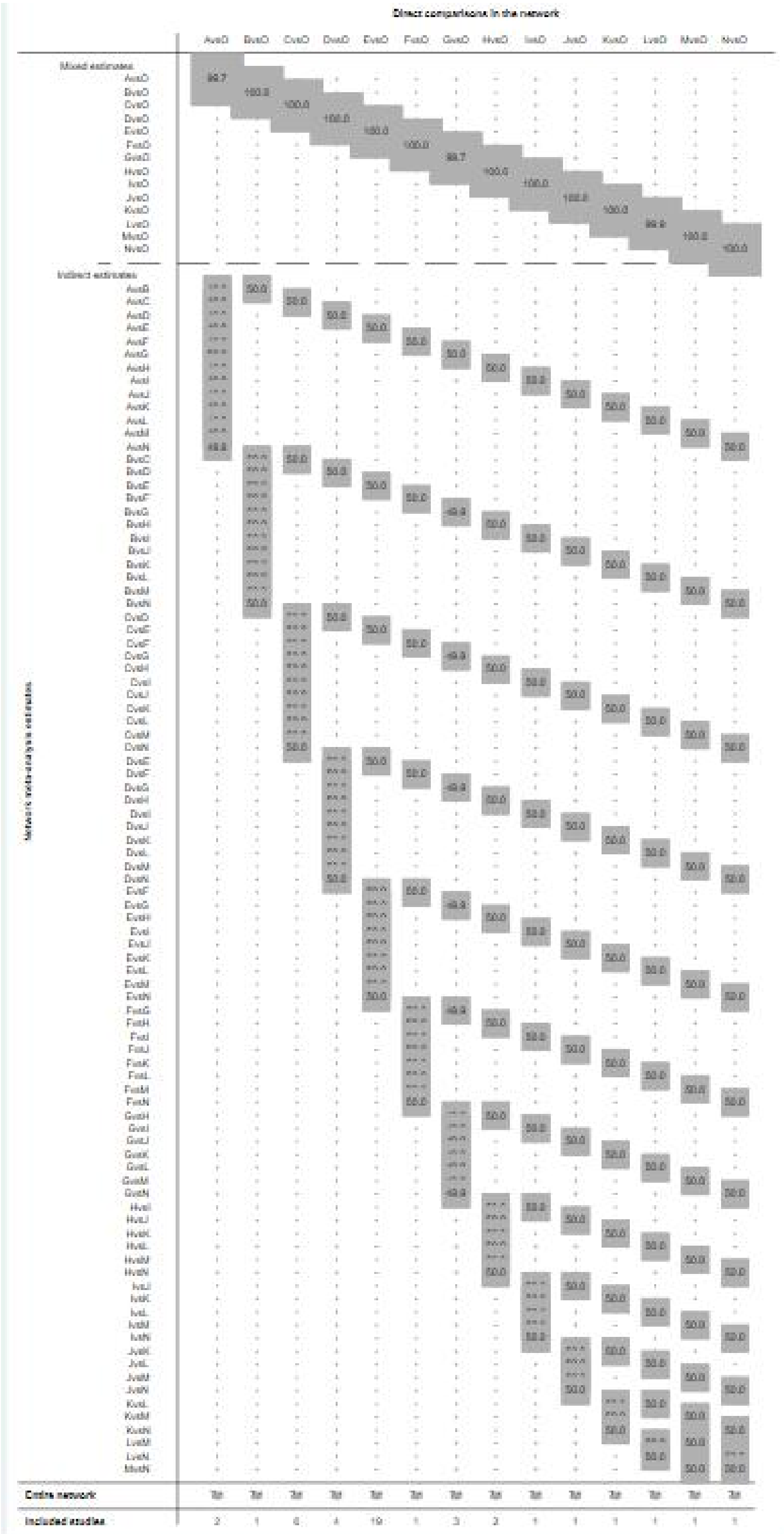

B

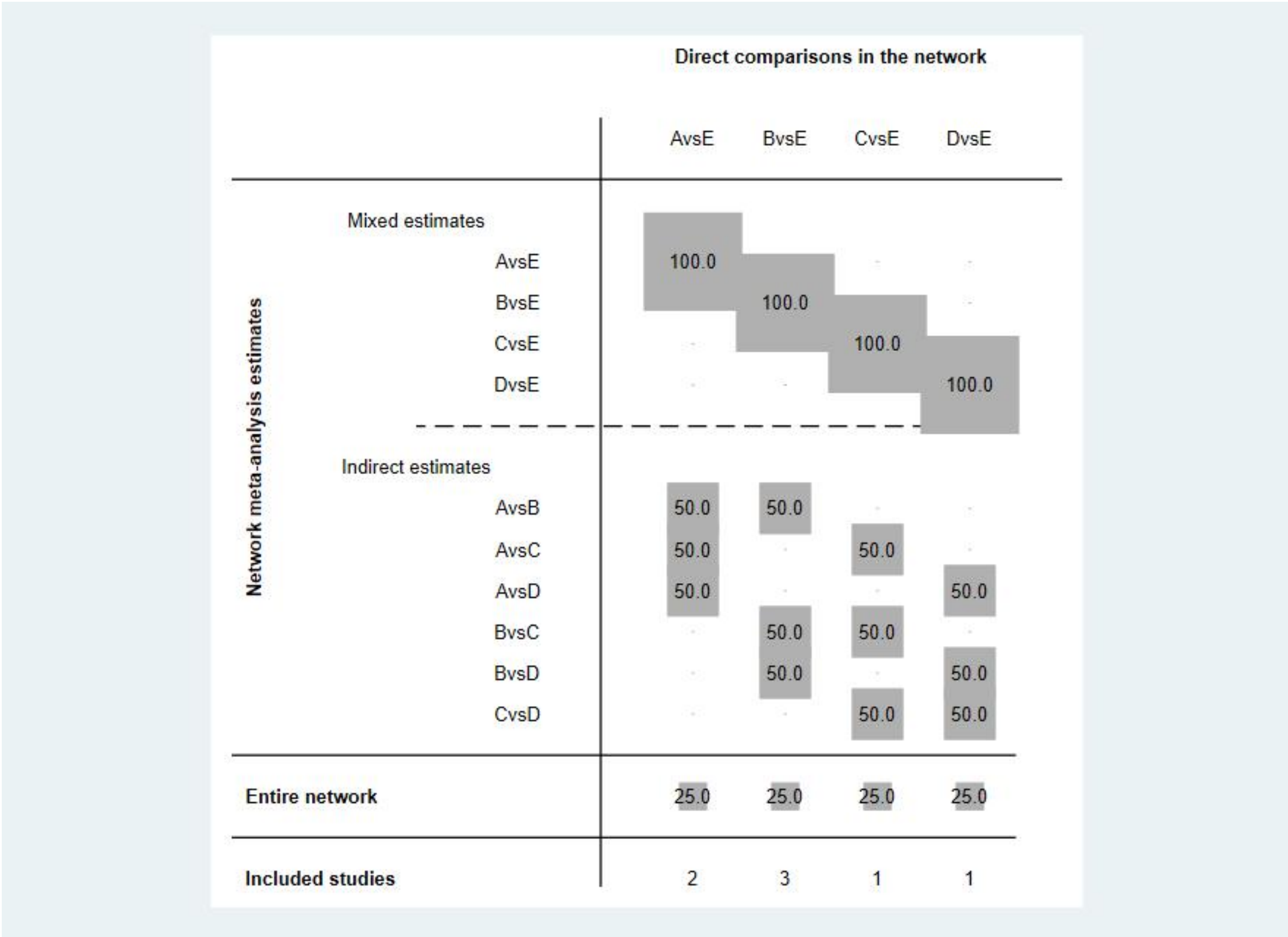
Direct comparisons in the network
AvsE
BvsE
CvsE
DvsE
Network meta-analysis estimates
Mixed estimates
AvsE
BvsE
CvsE
DvsE
100.0
100.0
100.0
100.0
Indirect estimates
AvsB
AvsC
AvsD
BvsC
BvsD
CvsD
50.0
50.0
50.0
50.0
50.0
50.0
50.0
50.0
50.0
50.0
50.0
50.0
Entire network
25.0
25.0
25.0
25.0
Included studies
2
3
1
1

C

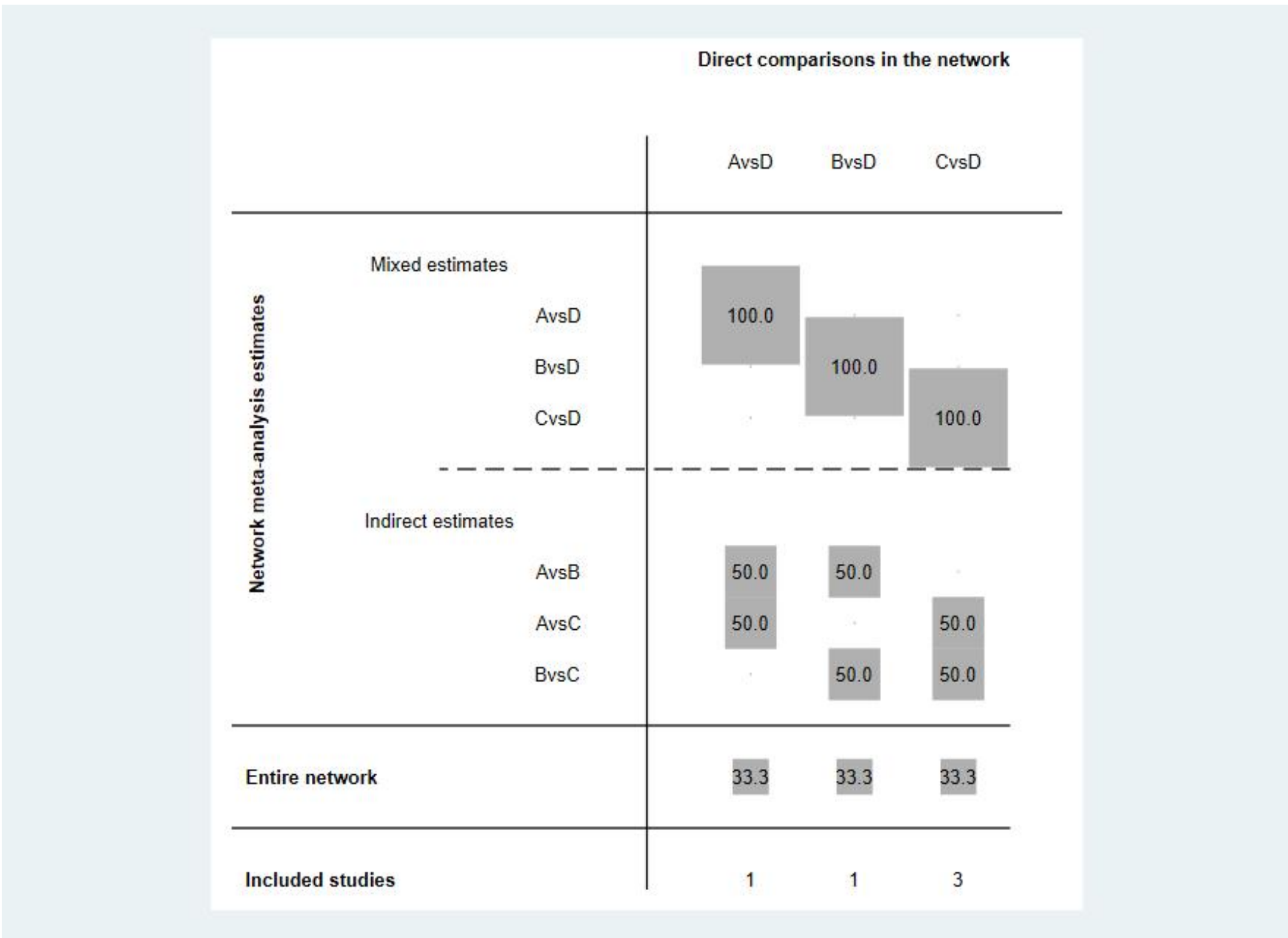
Direct comparisons in the network
AvsD
BvsD
CvsD
Network meta-analysis estimates
Mixed estimates
AvsD
BvsD
CvsD
100.0
100.0
100.0
Indirect estimates
AvsB
AvsC
BvsC
50.0
50.0
50.0
50.0
50.0
50.0
Entire network
33.3
33.3
33.3
Included studies
1
1
3

D

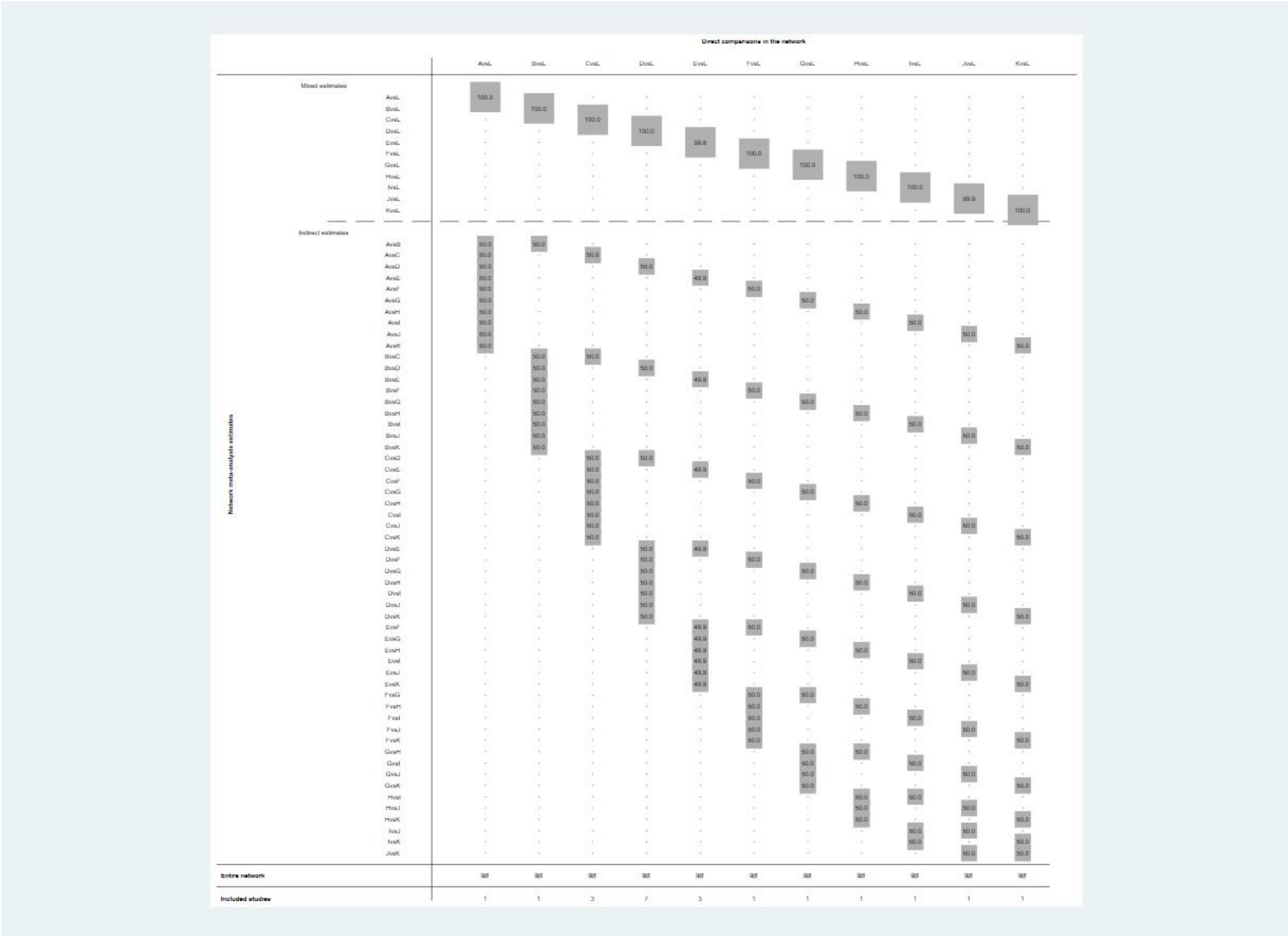

E

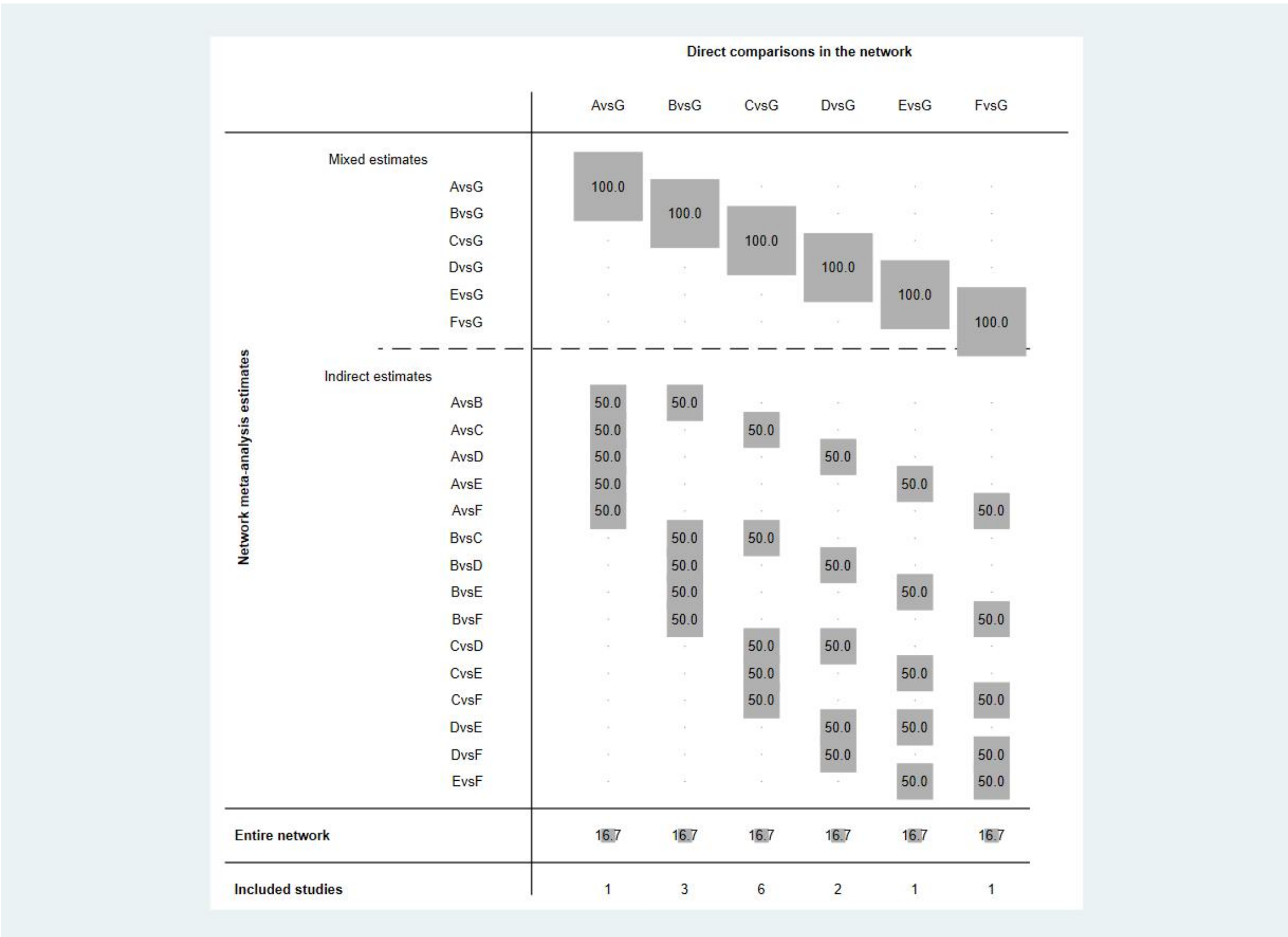
Direct comparisons in the network
AvsG
BvsG
CvsG
DvsG
EvsG
FvsG
Mixed estimates
AvsG
BvsG
CvsG
DvsG
EvsG
FvsG
Indirect estimates
AvsB
AvsC
AvsD
AvsE
AvsF
BvsC
BvsD
BvsE
BvsF
CvsD
CvsE
CvsF
DvsE
DvsF
EvsF
Network meta-analysis estimates
Entire network
Included studies

F

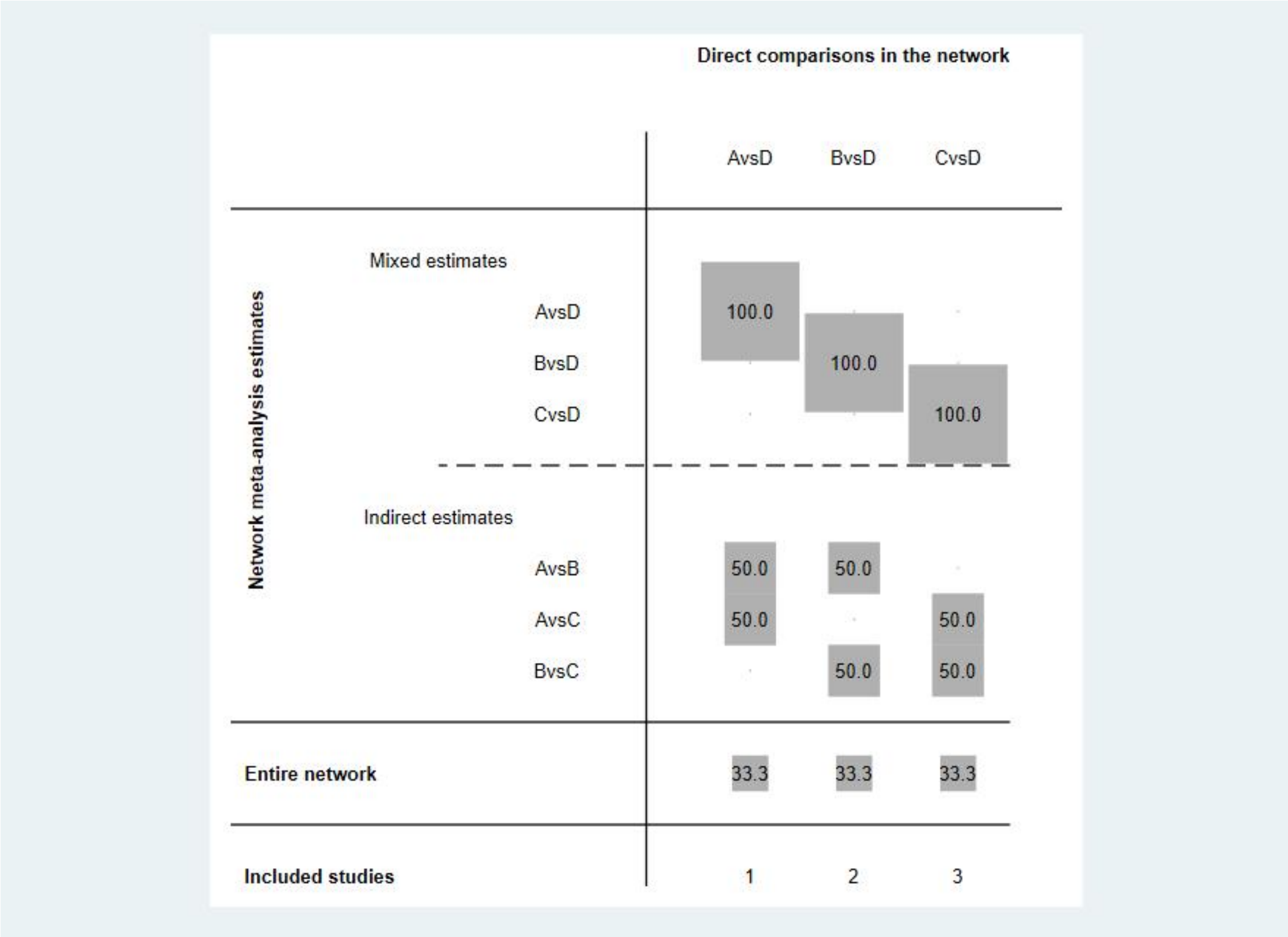
Direct comparisons in the network
AvsD
BvsD
CvsD
Network meta-analysis estimates
Mixed estimates
AvsD
BvsD
CvsD
100.0
100.0
100.0
Indirect estimates
AvsB
AvsC
BvsC
50.0
50.0
50.0
50.0
50.0
50.0
Entire network
33.3
33.3
33.3
Included studies
1
2
3

G

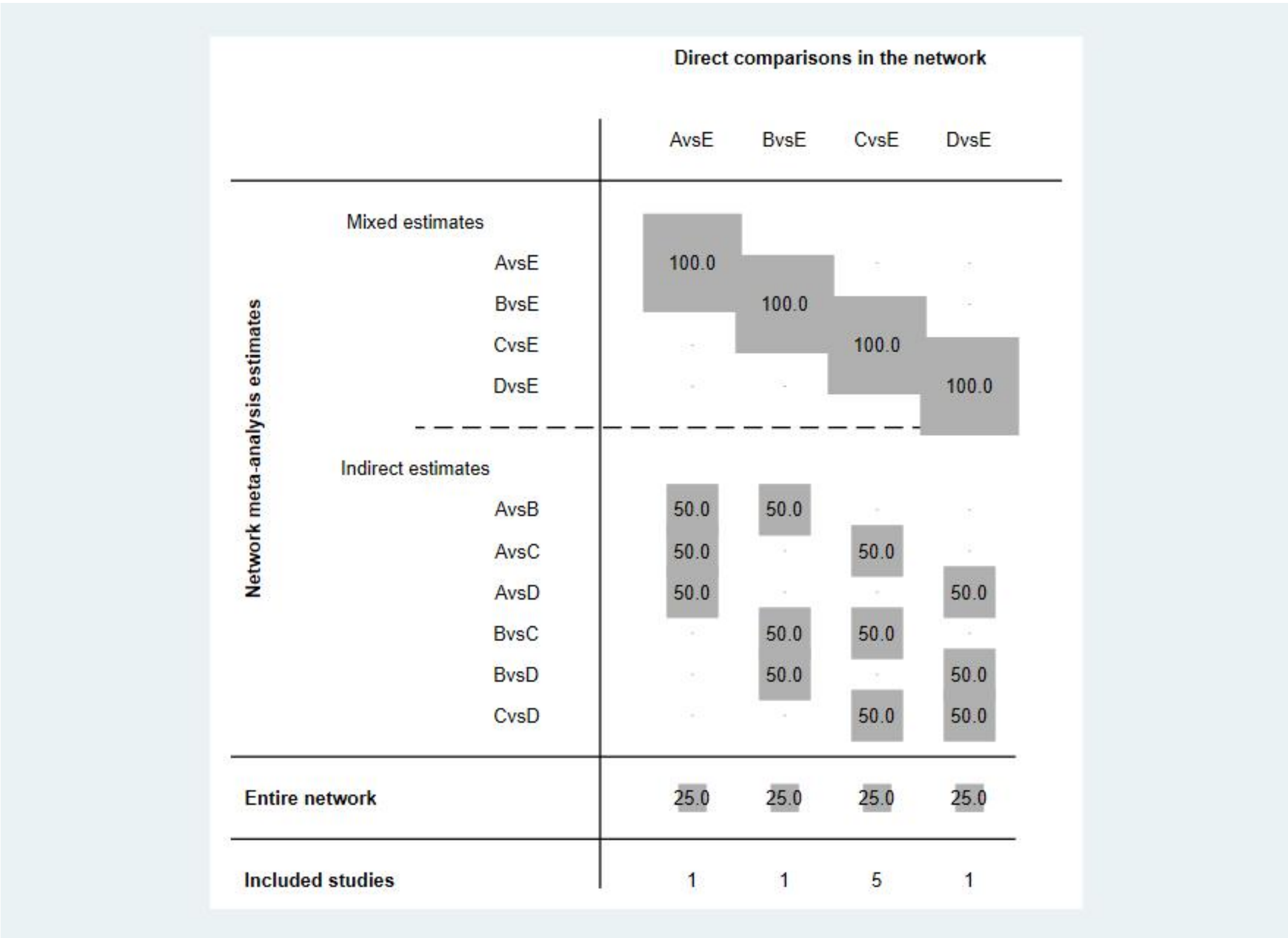
Direct comparisons in the network
AvsE
BvsE
CvsE
DvsE
Network meta-analysis estimates
Mixed estimates
AvsE
BvsE
CvsE
DvsE
100.0
100.0
100.0
100.0
Indirect estimates
AvsB
AvsC
AvsD
BvsC
BvsD
CvsD
50.0
50.0
50.0
50.0
50.0
50.0
50.0
50.0
50.0
50.0
50.0
50.0
Entire network
25.0
25.0
25.0
25.0
Included studies
1
1
5
1

H

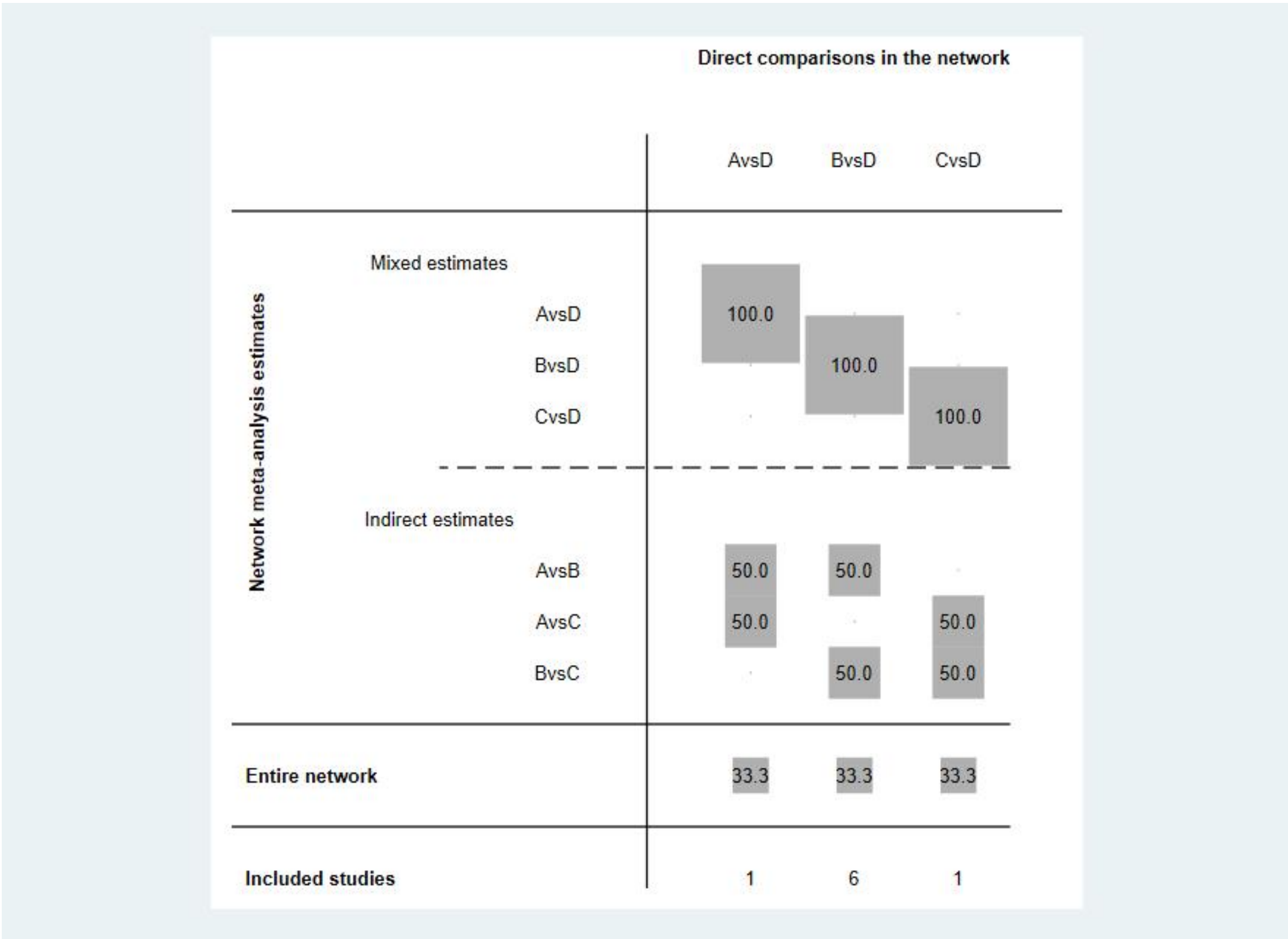
Direct comparisons in the network
AvsD
BvsD
CvsD
Network meta-analysis estimates
Mixed estimates
AvsD
BvsD
CvsD
100.0
100.0
100.0
Indirect estimates
AvsB
AvsC
BvsC
50.0
50.0
50.0
50.0
50.0
50.0
Entire network
33.3
33.3
33.3
Included studies
1
6
1

I

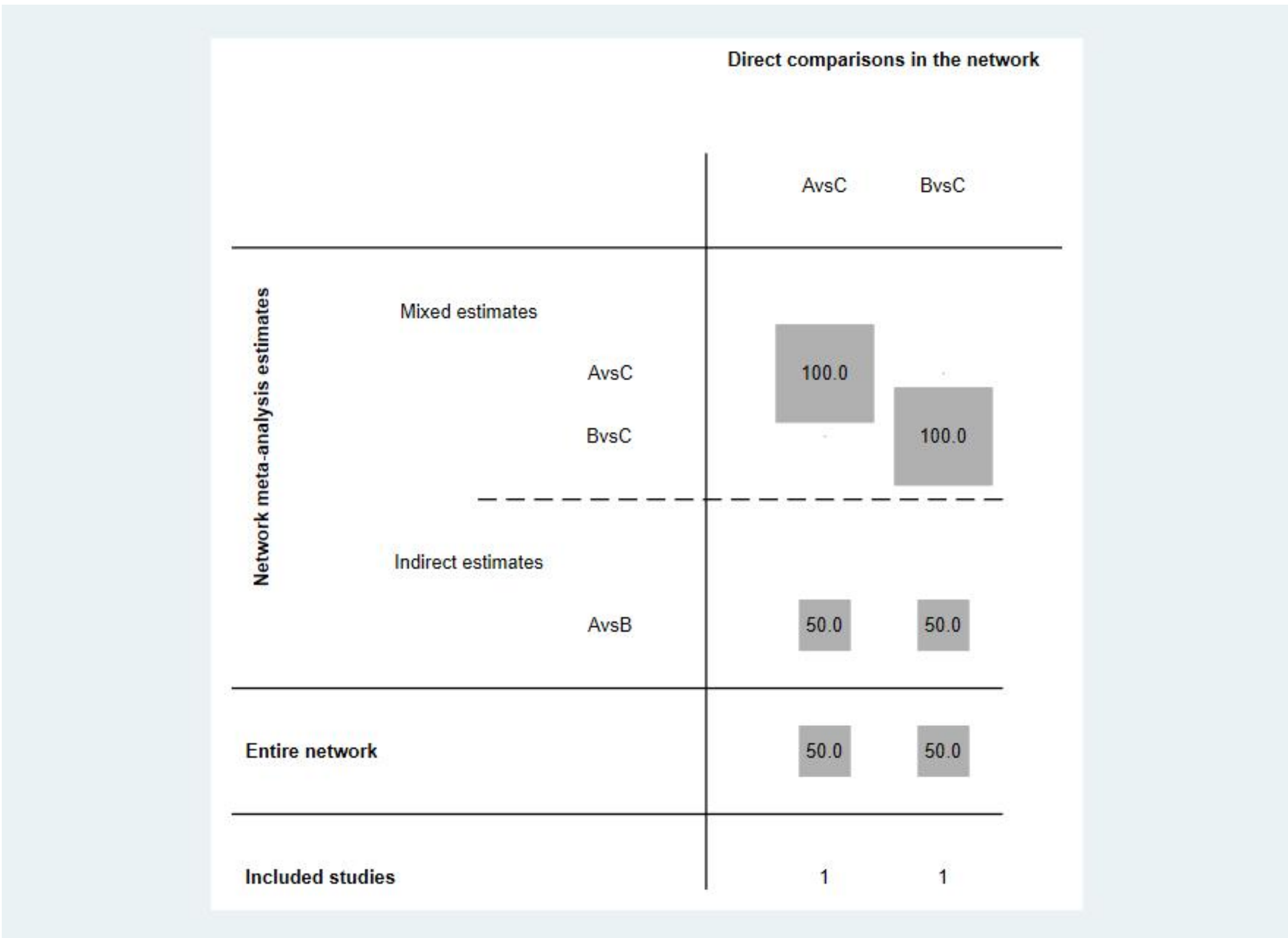
Direct comparisons in the network
AvsC
BvsC
Network meta-analysis estimates
Mixed estimates
AvsC
BvsC
100.0
100.0
Indirect estimates
AvsB
50.0
50.0
Entire network
50.0
50.0
Included studies
1
1
